# Galectin-9 recognizes and exhibits antimicrobial activity toward microbes expressing blood group–like antigens

**DOI:** 10.1016/j.jbc.2022.101704

**Published:** 2022-02-09

**Authors:** Anna V. Blenda, Nourine A. Kamili, Shang-Chuen Wu, William F. Abel, Diyoly Ayona, Christian Gerner-Smidt, Alex D. Ho, Guy M. Benian, Richard D. Cummings, Connie M. Arthur, Sean R. Stowell

**Affiliations:** 1 Department of Biomedical Sciences, University of South Carolina School of Medicine Greenville, Greenville, South Carolina, USA; 2 Department of Pathology and Laboratory Medicine, Emory University School of Medicine, Atlanta, Georgia, USA; 3 Joint Program in Transfusion Medicine, Department of Pathology, Brigham and Women’s Hospital, Harvard Medical School, Boston, Massachusetts, USA; 4 Department of Surgery, Beth Israel Deaconess Medical Center, Harvard Medical School, National Center for Functional Glycomics, Boston, Massachusetts, USA

**Keywords:** galectin-9, carbohydrate-binding protein, blood group antigen, glycan array, antimicrobial, β-ME, β-mercaptoethanol, BgB, blood group B, CFG, Consortium for Functional Glycomics, CRD, carbohydrate recognition domain, Gal-4, galectin-4, Gal-8, galectin-8, Gal-9, galectin-9, Gal-9C, C-terminal domain of Gal-9, Gal-9N, N-terminal domain of Gal-9, KPO1, *Klebsiella pneumoniae* O1, KPO4, *Klebsiella pneumoniae* O4, LacNAc, *N*-acetyl-lactosamine, LPS, lipopolysaccharide, MGM, microbial glycan microarray, PAO5, *Providencia alcalifaciens* O5, PAO19, *Providencia alcalifaciens* O19, PBS-T, PBS + Tween-20, polyLacNAc, polylactosamine, RBC, red blood cell, TDG, thiodigalactoside

## Abstract

While adaptive immunity recognizes a nearly infinite range of antigenic determinants, immune tolerance renders adaptive immunity vulnerable to microbes decorated in self-like antigens. Recent studies suggest that sugar-binding proteins galectin-4 and galectin-8 bind microbes expressing blood group antigens. However, the binding profile and potential antimicrobial activity of other galectins, particularly galectin-9 (Gal-9), has remained incompletely defined. Here, we demonstrate that while Gal-9 possesses strong binding preference for ABO(H) blood group antigens, each domain exhibits distinct binding patterns, with the C-terminal domain (Gal-9C) exhibiting higher binding to blood group B than the N-terminal domain (Gal-9N). Despite this binding preference, Gal-9 readily killed blood group B–positive *Escherichia coli*, whereas Gal-9N displayed higher killing activity against this microbe than Gal-9C. Utilization of microarrays populated with blood group O antigens from a diverse array of microbes revealed that Gal-9 can bind various microbial glycans, whereas Gal-9N and Gal-9C displayed distinct and overlapping binding preferences. Flow cytometric examination of intact microbes corroborated the microbial glycan microarray findings, demonstrating that Gal-9, Gal-9N, and Gal-9C also possess the capacity to recognize distinct strains of *Providencia alcalifaciens* and *Klebsiella pneumoniae* that express mammalian blood group–like antigens while failing to bind related strains that do not express mammalian-like glycans. In each case of microbial binding, Gal-9, Gal-9N, and Gal-9C induced microbial death. In contrast, while Gal-9, Gal-9N, and Gal-9C engaged red blood cells, each failed to induce hemolysis. These data suggest that Gal-9 recognition of distinct microbial strains may provide antimicrobial activity against molecular mimicry.

Adaptive immunity can recognize a nearly infinite range of antigens, allowing hosts to target an ever-evolving antigenic landscape (1). However, the ability of host immunity to target evolving antigenic determinants also increases the risk of self-reactivity (2, 3, 4, 5, 6). To reduce the probability of self-injury, a series of immune programs evolved that delete self-reactive cells or render them unresponsive (7, 8). When these programs fail, individuals lose tolerance to self, which can result in autoimmunity (3). In the vast majority of individuals, immune tolerance successfully limits self-reactivity, allowing host protection to occur while limiting injury to self. However, the development of immunological tolerance toward self may also create gaps in adaptive immunity. Removal of self-reactivity reduces the ability of adaptive immunity to recognize microbes that decorate themselves in self-like antigens. Indeed, the use of molecular mimicry by microbes may in part have evolved as a mechanism of avoiding adaptive immunity (9).

ABO blood group antigen polymorphisms provide one of the first recognized examples of immunological tolerance within adaptive immunity (10, 11). Individuals who express blood group B (BgB) do not generate anti-B antibodies, whereas blood group O individuals, who do not express the BgB antigen, readily form anti-B antibodies (10). While the failure of BgB individuals to generate anti-B antibodies likely protects BgB-expressing cells, such as red blood cells (RBCs), from immune-mediated destruction (10), this form of tolerance creates an important gap in adaptive immunity toward blood group–expressing microbes (12). Given the limitations in adaptive immunity against blood group–expressing microbes (8), innate immune factors may have evolved to fill this gap and therefore protect blood group–positive individuals from blood group–positive microbes. Consistent with this, recent results suggest that several galectins, which belong to a family of innate immune lectins, possess the capacity to specifically engage blood group–positive microbes (13). Galectin-4 (Gal-4) and galectin-8 (Gal-8), which are expressed along the gastrointestinal tract, have been previously shown to not only bind blood group antigens but also exhibit the ability to recognize blood group antigen–positive microbes (13). Gal-4 and Gal-8 recognition of blood group–positive microbes results in microbial death, providing a direct example of a unique form of innate immunity against molecular mimicry (13).

Despite recent studies demonstrating that Gal-4 and Gal-8 possess antimicrobial activity, the potential ability of other galectins to likewise recognize and kill microbes that express blood group antigens remains incompletely understood. Not all galectins possess antimicrobial activity. For example, galectin-1, one of the most well-studied members of the galectin family (14), fails to recognize or kill microbes expressing these antigenic determinants (13). However, the possible ability of other galectins to both recognize and impact the viability of blood group–positive microbes remains relatively unknown. In particular, galectin-9 (Gal-9), which has also been shown to be expressed along the intestinal mucosa (15, 16) and shares the same tandem repeat configuration as Gal-4 and Gal-8 (17), has not been examined in detail for its blood group binding specificity or its possible ability to kill microbes.

In this study, we used a series of glycan libraries to define the specificity of human Gal-9 toward these polymorphic blood group antigen carbohydrates. To accomplish this, we examined the binding specificity and antimicrobial activity of full-length Gal-9 and each of its individual domains, as Gal-9 is a tandem repeat galectin consisting of two distinct carbohydrate recognition domains (CRDs). Using this approach, our data demonstrate that while Gal-9 recognizes blood group antigens, the N-terminal domain of galectin-9 (Gal-9N) and the C-terminal domain of galectin-9 (Gal-9C) possess subtle yet distinct binding preferences toward subgroups of blood group A and B. These binding specificities accurately predict actual interactions with intact microbes. However, Gal-9, Gal-9N, and Gal-9C all recognize blood group–positive microbes, whereas only Gal-9 and Gal-9N readily induced microbial death of group B-positive *Escherichia coli*. However, expanded analysis of microbial glycan binding demonstrated that Gal-9, Gal-9N, and Gal-9C all possessed the ability to recognize distinct microbial glycans with blood group–related structures. When examined against intact microbes represented in this microbial glycan microarray (MGM) format, Gal-9 and each domain exhibited the ability to specifically bind and kill several microbial targets. Taken together, these studies demonstrate that Gal-9 and its individual domains possess the ability to bind and kill microbes that express self-like antigens and therefore may contribute to innate immunity against molecular mimicry.

## Results

### Gal-9 engages a wide variety of blood group antigens, whereas each domain displays distinct blood group subtype preferences

**Figure 1:**
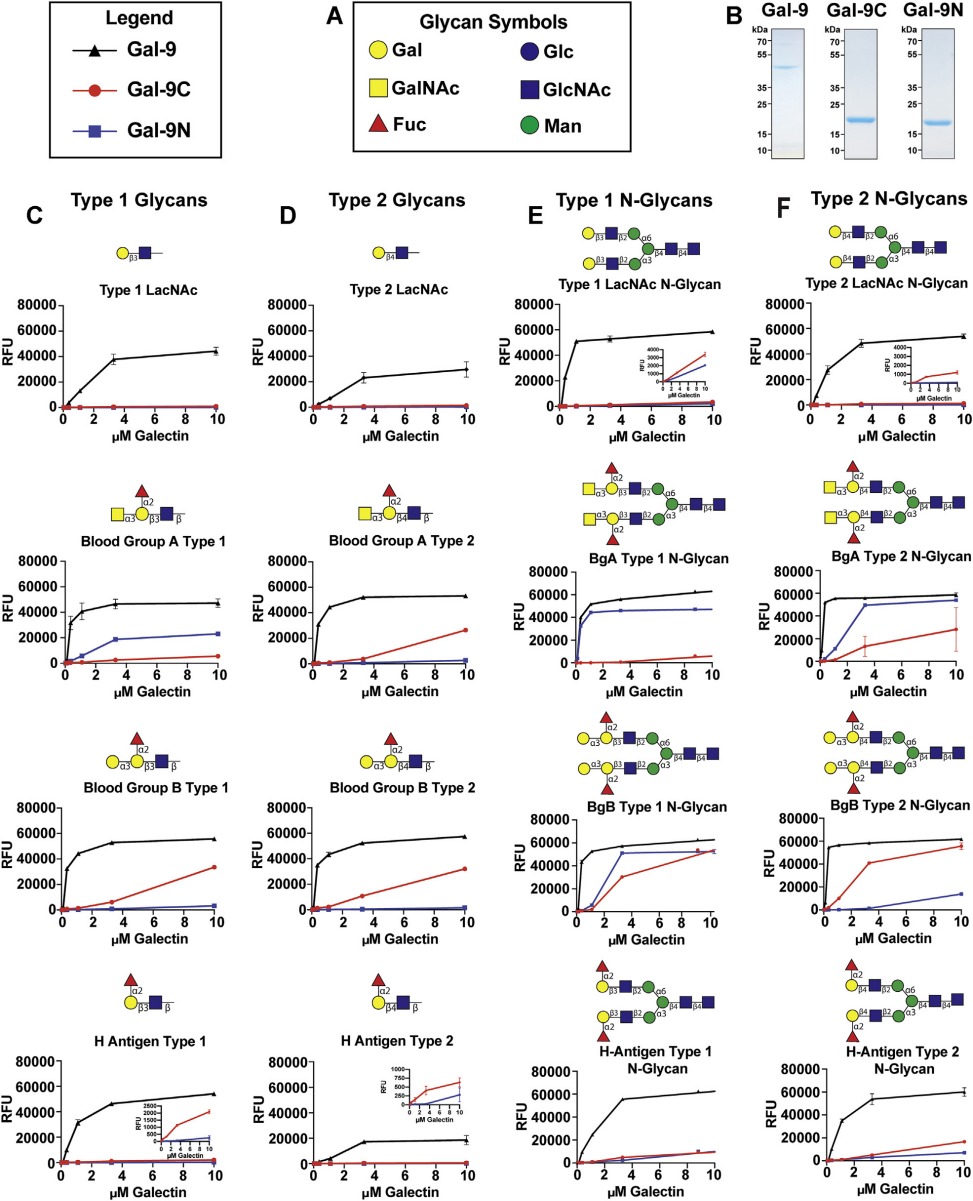
**Binding of galectin-9 (Gal-9) and its domains to LacNAc and blood group antigens on CFG glycan microarray.***A*, legend of glycan structures: Gal = galactose, GalNAc = *N*-acetylgalactosamine, Glc = glucose, GlcNAc = *N*-acetylglucosamine, Man = mannose, and Fuc = fucose. *B*, SDS-PAGE analysis of Gal-9, Gal-9C, and Gal-9N. *C*–*F*, recognition of each represented glycan by Gal-9 (*black*), Gal-9N (*blue*), and Gal-9C (*red*) of varying concentrations (0.04–10 μM) on CFG glycan microarray, expressed in relative fluorescence units (RFUs) is shown with each glycan structure followed by its trivial name above each graph (LacNAc = lactosamine, BgA = blood group A, and BgB = blood group B). *C*, type 1 LacNAc and blood group–containing glycans. *D*, type 2 LacNAc and blood group–containing glycans. *E*, type 1 LacNAc and blood group–containing *N*-glycans. *F*, type 2 LacNAc and blood group–containing *N*-glycans. For better appreciation of subtle differences in binding, insets are included with some plots. Error bars represent standard deviation among six replicates. CFG, Consortium for Functional Glycomics; Gal-9C, C-terminal domain of Gal-9; Gal-9N, N-terminal domain of Gal-9.

To define the binding specificity of Gal-9 and its domains toward polymorphic blood group antigens, we generated the full-length Gal-9 protein in addition to the individual Gal-9N and Gal-9C domains. Use of all three proteins allowed examination of the glycan binding specificity of the full-length Gal-9 protein and the potential contribution of the individual domains to the overall glycan-binding profile of Gal-9. To assess the glycan-binding profile of Gal-9, Gal-9N, and Gal-9C, we first employed the widely used glycan microarray from the Consortium for Functional Glycomics (CFG) array. Many prior studies, including our own, have primarily assessed galectin engagement of glycans in an array format using a single concentration (18, 19). Using this rank order approach, general differences in glycan-binding preferences have been observed. However, to examine in more detail the possibility of more subtle differences in glycan-binding activity, with a focus on distinct types of blood group antigens, we examined Gal-9, Gal-9N, and Gal-9C over a range of concentrations. Using this approach, binding isotherms can be obtained in an effort to ascertain the relative affinity of Gal-9 and each domain for glycan determinants, including blood group–containing glycans. While Gal-9 readily recognized *N*-acetyl-lactosamine (LacNAc) alone, N glycans, and other LacNAc-containing glycans, it exhibited some of the highest binding toward polylactosamine (polyLacNAc) and A or B blood group antigens (Figs. 1, *C*–*F* and S1; Tables S1 and S4). In contrast to the enhanced binding observed toward A or B blood group antigens, very little difference was observed between LacNAc and LacNAc bearing the H antigen (Fig. 1, *C*–*F*). While polyLacNAc glycans have been suggested as preferred glycans on leukocytes through which galectins may regulate their function (20, 21), less is known regarding the impact of subtle variations in blood group presentation on Gal-9 interactions. This is especially important when considering that the presentation of blood group–like antigens on microbes may vary.

As Gal-9 exists as a tandem repeat galectin consisting of two distinct domains, we explored the relative specificity of Gal-9 and each domain toward distinct types of blood group antigens on the CFG array. Unlike Gal-9, which readily binds to both blood group A and B antigens regardless of linkage and presentation (Fig. 1, *C*–*F*), each individual domain of Gal-9 demonstrated more distinct preferences for blood group antigens (Fig. 1, *C*–*F*). This can be especially appreciated when examining type 1 (Galβ1–3GlcNAc) *versus* type 2 (Galβ1–4GlcNAc) LacNAc containing blood group antigens. Though neither domain exhibited appreciable interactions with type 1 or type 2 LacNAc alone, as shown in Figure 1, *C* and *D*, LacNAc configurations clearly impacted blood group recognition by each domain, with Gal-9N possessing relatively high affinity for type 1 blood group A (Fig. 1*C*), whereas blood group A presented on a type 2 structure failed to support similar binding activity (Fig. 1*D*). The opposite was observed for Gal-9C. Little relative binding was detected by Gal-9C for type 1 blood group A (Fig. 1*C*), whereas high binding was apparent for type 2 blood group A (Fig. 1*D*). In contrast to the ability of type 1 and type 2 structures to influence blood group A recognition, Gal-9C exhibited higher binding than Gal-9N to BgB regardless of the penultimate GlcNAc linkage within LacNAc (Fig. 1, *C* and *D*). Unlike Gal-9 binding to blood groups A and B, which is unaffected by linkage, Gal-9 exhibits a preference for type 1 H antigen compared with type 2 H antigen (Fig. 1, *C* and *D*). When examining affinities of the individual domains, Gal-9C displayed higher binding to type 1 and type 2 H antigens when compared with that observed for Gal-9N (Fig. 1, *C* and *D*).

Given the apparent impact of blood group presentation on the ability of Gal-9N and Gal-9C to bind blood group A antigens and previous data suggesting that Galβ1-3,4GlcNAc presentation on the terminal structures of other core glycans may ultimately influence glycan recognition (20, 22, 23), we next examined the possible influence of N glycan presentation of blood group antigens on Gal-9, Gal-9N, and Gal-9C binding. Gal-9 readily bound N glycans in general, and the presence of blood group A or B antigens on N glycans appeared to enhance this interaction, with similar overall apparent affinity observed toward different blood group antigen presentations in this context (Fig. 1, *E* and *F*). In fact, when compared with the trisaccharide configuration, presentation of type 2 H antigen as a terminal modification of N-glycan enhances Gal-9 recognition (Fig. 1, *D* and *F*).

Gal-9N and Gal-9C displayed very little binding toward biantennary N glycans bearing type 1 LacNAc or type 2 LacNAc structures (Fig. 1, *E* and *F*), whereas expression of blood group A, B, or H antigens on these N glycans appeared to enhance recognition (Fig. 1, *E* and *F*). These findings correlate with previous reports that in addition to terminal glycan antigens, complex glycan architecture can influence binding (24). In contrast to the binding of Gal-9N and Gal-9C toward blood group A as type 1 or type 2 tetrasaccharides, in the context of a biantennary N glycan, Gal-9N displayed binding higher than that of Gal-9C to both structures in the context of a bianternnary N glycan (Fig. 1, *C*–*F*). N glycan presentation of BgB likewise influenced the binding of each domain, as Gal-9N now exhibited higher binding toward type 1 BgB presented on an N glycan structure, while continuing to display relatively lower binding toward type 2 BgB capping an N glycan (Fig. 1, *C*–*F*). Each Gal-9 domain exhibited a less pronounced enhancement in binding following H antigen modification of N glycans regardless of the terminal type 1 or type 2 linkage (Fig. 1, *C*–*F*). Taken together, these results suggest that while Gal-9 readily engages distinct blood group antigens on N glycans, each Gal-9 domain likewise possesses the ability to distinctly engage blood group antigens in this context, with the underlying linkage (*e.g.*, type 1 or type 2) and overall glycan presentation influencing these interactions.

### Gal-9 targets BgB-positive *E. coli* primarily through its N-terminal domain

**Figure 2:**
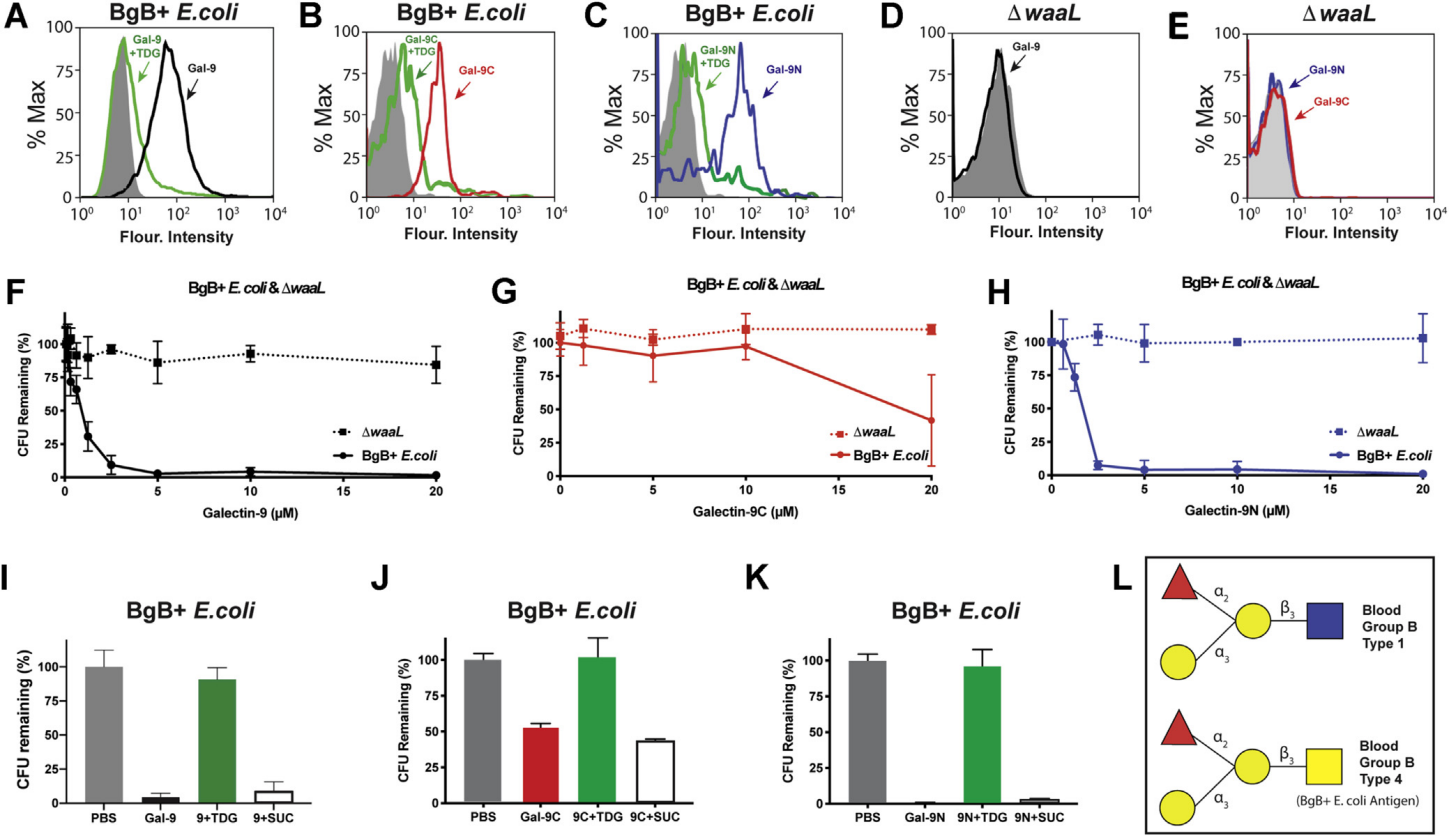
**Galectin-9 (Gal-9) recognizes and kills blood group B–expressing *Escherichia coli* (BgB+ *E. coli*).***A*–*C*, flow cytometric analysis of BgB+ *E. coli* binding after incubation with Gal-9 (*A*, *black*), Gal-9C (*B*, *red*), and Gal-9N (*C*, *blue*) with or without 20 mM TDG (*green*) and streptavidin control (*solid gray*). *D* and *E*, flow cytometric analysis of BgB− *E. coli* (Δ*waaL*) binding after incubation with Gal-9 (*D*, *black*), Gal-9C (*E*, *red*), Gal-9N (*E*, *blue*), and streptavidin control (*solid gray*). *F*–*H*, colony-forming units (CFUs) quantification of BgB+ *E. coli* or Δ*waaL* after incubation with varying concentrations of Gal-9 (*F*), Gal-9C (*G*), and Gal-9N (*H*). *I*–*K*, CFU quantification of BgB+ *E. coli* after incubation with PBS control, 10 μM Gal-9 (*I*), 20 μM Gal-9C (*J*), or 10 μM Gal-9N (*K*) with or without 20 mM TDG and 20 mM sucrose as controls. *L*, glycan structures of type 4 blood group B antigen expressed on BgB+ *E. coli* and the type 1 blood group B antigen. Error bars represent standard deviation among three experimental replicates. Results are representative of three independent experiments. Gal-9C, C-terminal domain of Gal-9; Gal-9N, N-terminal domain of Gal-9; TDG, thiodigalactoside.

Prior results suggest that galectin interactions on the CFG array can accurately predict actual interactions with BgB-expressing microbes (13). As Gal-9, *via* the C-terminal domain in particular, appeared to exhibit high binding to the BgB antigen on the CFG array, both as a tetrasaccharide and in the context of N glycan presentation, we next examined whether Gal-9 and its domains possess the ability to recognize BgB-expressing microbes (Fig. 2). The examination of individual domains is important as previous data suggested that while Gal-8 possesses antimicrobial activity, only the C-terminal domain of Gal-8 exhibits the ability to kill microbes (13). To accomplish this, we examined Gal-9, Gal-9C, and Gal-9N interactions with *E. coli* O86 (BgB+ *E. coli*), previously shown to express the BgB antigen (25). Flow cytometric analysis demonstrated that Gal-9 and Gal-9C bind to BgB+ *E. coli*, suggesting that CFG glycan microarray results can indeed predict actual interactions with intact microbes (Fig. 2, *A* and *B*). To determine whether this binding required carbohydrate interactions as opposed to possible carbohydrate-independent binding, thiodigalactoside (TDG), a carbohydrate inhibitor of galectin interactions (26), was coincubated with Gal-9 and Gal-9C. TDG inclusion significantly attenuated this binding, strongly suggesting that Gal-9 and Gal-9C engage BgB+ *E. coli* through recognition of cell surface carbohydrates (Fig. 2, *A* and *B*). To determine whether this interaction was specific to Gal-9C, we next examined whether similar binding occurs following incubation of BgB+ *E. coli* with Gal-9N. Like Gal-9C, Gal-9N bound to BgB+ *E. coli* with TDG inhibiting the interaction (Fig. 2*C*), suggesting that unlike binding toward BgB tetrasaccharides on the CFG array, both domains of Gal-9 appear to recognize BgB+ *E. coli*.

Given differences between array findings and our analysis of BgB+ *E. coli* interactions with Gal-9 domains, we next sought to determine whether binding of each domain to BgB+ *E. coli* relied on BgB expression. To accomplish this, we examined *via* flow cytometry possible interactions with the Δ*waaL* mutant of BgB+ *E. coli* that specifically fails to express the BgB antigen (25). Using this approach, we found that neither Gal-9 and Gal-9N nor Gal-9C exhibited binding toward Δ*waaL* (Fig. 2, *D* and *E*), strongly suggesting that the observed interactions require BgB expression. The binding of Gal-9 and Gal-9C toward BgB antigens on the CFG array coupled with its interaction with BgB+ *E. coli* suggest that Gal-9, and Gal-9C in particular, may possess antimicrobial activity, similar to that previously reported for Gal-8 and C-terminal domain of Gal-8 (13). To test this, we next examined the possible antimicrobial activity of Gal-9 and each of its domains. Gal-9 reduced the viability of BgB+ *E. coli* (Fig. 2*F*), whereas Gal-9C only impacted microbial viability at higher concentrations (Fig. 2*G*). In contrast to Gal-9C, Gal-9N exhibited more potent killing ability, reducing the viability of BgB+ *E. coli* at concentrations much lower than those observed for Gal-9C (Fig. 2*H*). Similar to binding, the antimicrobial impact of Gal-9, Gal-9C, and Gal-9N on BgB+ *E. coli* relied on blood group recognition, as Gal-9 and its domains failed to kill the Δ*waaL* mutant (Fig. 2, *F*–*H*). Furthermore, even at the higher galectin concentrations tested, TDG, but not sucrose, inhibited the killing activity (Fig. 2, *I*–*K*), providing additional evidence that binding and killing require carbohydrate recognition.

### Gal-9N exhibits higher binding to BgB glycans represented on group B-positive *E. coli*

**Figure 3:**
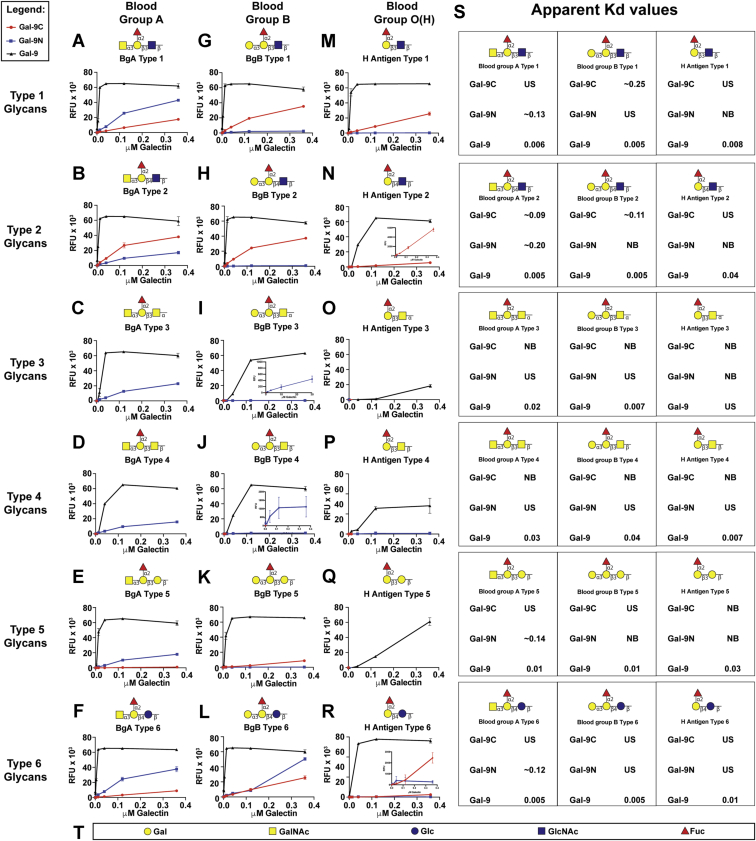
**Galectin-9 (Gal-9), N-terminal domain of Gal-9 (Gal-9N), and C-terminal domain of Gal-9 (Gal-9C) affinity for blood group antigens on the ABH glycan microarray.** Glycan structures followed by trivial names of each glycan tested on the ABH glycan microarray are shown above each plot. Recognition of each represented glycan by Gal-9 (*black*), Gal-9N (*blue*), and Gal-9C (*red*) of varying concentrations is shown in relative fluorescence units (RFUs) on plots. Gal-9, Gal-9N, and Gal-9C binding to blood group A antigens (*A*–*F*); Gal-9, Gal-9N, and Gal-9C binding to blood group B antigens (*G*–*L*); and Gal-9, Gal-9N, and Gal-9C binding to H-antigen structures (*M*–*R*). For better appreciation of subtle differences in relative binding, plots *I*, *J*, and *R* include magnified insets. *S*, table of apparent *K*_*d*_ values. US = *K*_*d*_ could not be calculated because of unsaturated galectin–glycan binding. NB = no binding (galectin–glycan binding was not detectable). *T*, legend of glycan structures: Gal = galactose, GalNAc = *N*-acetylgalactosamine, Glc = glucose, GlcNAc = *N*-acetylglucosamine, and Fuc = fucose. Error bars represent standard deviation among six replicates.

Given the apparent discrepancy between Gal-9C binding on the CFG glycan microarray and its killing activity toward BgB+ *E. coli*, coupled with the influence of subtle changes to BgB presentation on Gal-9N and Gal-9C binding, we next sought to explore Gal-9N and Gal-9C interactions with blood group antigens in more detail. To this end, we examined Gal-9, Gal-9N, and Gal-9C binding toward a broad series of blood group antigens presented on six core structures (types 1–6) assembled in an A, B, and H blood group–specific microarray (ABH array) (Fig. 3). Using this approach, we found that similar to the findings obtained following CFG array analysis, full-length Gal-9 exhibited high binding to the majority of the blood group antigens tested. In contrast, Gal-9C exhibited a preference for type 2 blood group A when compared with the same blood group antigen presented on type 1 structures (Fig. 3, *A* and *B*). However, Gal-9C largely failed to bind blood group A antigens presented on type 3, 4, 5, and 6 structures, whereas Gal-9N exhibited measurable interactions with each of these structures (Fig. 3, *C*–*F*). Consistent with CFG array results, Gal-9C preferred BgB presented on type 1 and type 2 structures (Fig. 3, *G* and *H*), whereas neither domain exhibited high-level binding toward type 3, 4, or 5 structures (Fig. 3, *I*–*K*) and displayed marginal binding toward type 6 BgB (Fig. 3*L*). Compared with Gal-9N, Gal-9C also displayed higher binding toward type 1 and type 2 H antigen structures (Fig. 3, *M* and *N*). It should be noted that upon closer analysis, though Gal-9C fails to bind, Gal-9N displayed some binding toward type 4 BgB (Fig. 3*J*, inset), which matches the linkage found in BgB+ *E. coli*. Taken together, these results suggest that the CFG array and the expanded ABH blood group antigen array outcomes are largely in agreement, and that distinct forms of blood group presentation can differentially impact blood group recognition by each Gal-9 domain. Furthermore, the ability of Gal-9N to bind type 4 blood group structures, albeit at a low level, may provide some insight into the increased killing activity of Gal-9N displayed toward BgB+ *E. coli*.

### Gal-9, Gal-9N, and Gal-9C specifically recognize a variety of microbial glycans with mammalian glycan features

**Figure 4:**
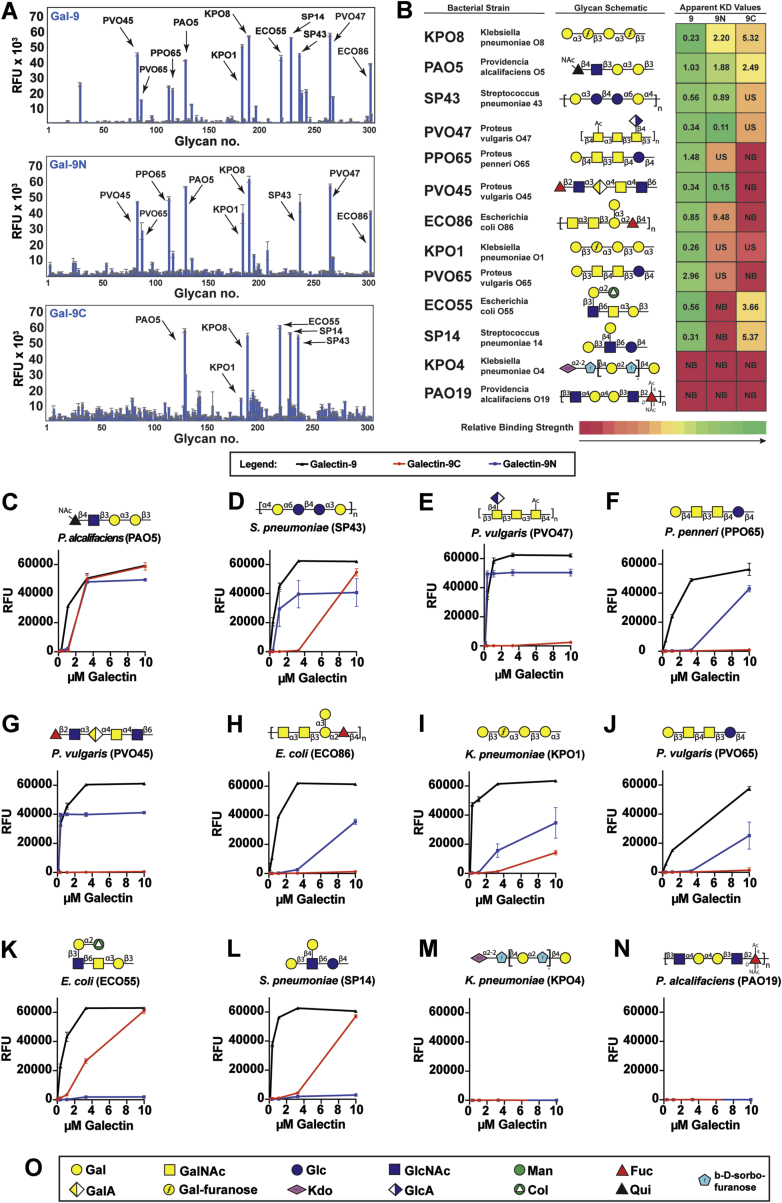
**Binding of galectin-9 (Gal-9) and its domains on microbial glycan microarray.** Gal-9, Gal-9N, and Gal-9C were incubated on MGM array, and binding strength of microbial glycans to each galectin was measured in relative fluorescence units (RFUs). *A*, representative MGM array data of Gal-9 (1.1 μM), Gal-9N (10 μM), and Gal-9C (10 μM). *B*, selected microbial glycan structures and names of the originating species are presented along with a table of apparent *K*_*d*_ values on a heat map depicting relative binding strength. US = *K*_*d*_ could not be calculated because of unsaturated galectin–glycan binding. NB = no binding (galectin–glycan binding was not detectable). *C*–*N*, microbial glycan structures with corresponding dose curves of glycan binding over a range of galectin concentrations of Gal-9 (*black curve*), Gal-9N (*blue curve*), and Gal-9C (*red curve*). *O*, legend of glycan structures used: Col = colitose, Fuc = fucose, Gal = galactose, GalA = d-galacturonic acid, Gal-furanose = galactofuranose, GalNac = *N*-acetylgalactosamine, Glc = glucose, GlcA = d-glucuronic acid, GlcNac = *N*-acetylglucosamine, Kdo = 2-deoxy-d-manno-octulosonic acid, Man = mannose, and Qui = d-quinvose. Error bars represent standard deviation among six replicates. Gal-9C, C-terminal domain of Gal-9; Gal-9N, N-terminal domain of Gal-9; MGM, microbial glycan microarray.

The reduced ability of the CFG and ABH blood group arrays to predict actual interactions with BgB+ *E. coli* may in part reflect the influence of the distinct nature of blood group antigens and other self-like antigen presentation within the context of microbial glycans on Gal-9, Gal-9N, and Gal-9C recognition. To examine this in more detail, we next turned to an MGM populated with lipopolysaccharides (LPSs) isolated from several previously characterized microbes, including those that express blood group and related mammalian-like antigens (Figs. 4 and S2; Tables S2, S3 and S5). Using this approach, Gal-9 readily engaged a variety of microbial glycan determinants, whereas Gal-9N and Gal-9C displayed overlapping and distinct glycan-binding preferences. For example, examination of both domains over a range of concentrations demonstrated that Gal-9N bound with much higher affinity than Gal-9C toward the LPS of BgB+ *E. coli* (Fig. 4, *A*, *B* and *H*). Gal-9 exhibited higher binding to BgB+ *E. coli* than either domain alone (Fig. 4, *A*, *B* and *H*).

In contrast to the differential binding of each Gal-9 domain that occurred toward the LPS of BgB+ *E. coli*, a similar binding profile was observed between Gal-9, Gal-9N, and Gal-9C toward the LPS isolated from *Providencia alcalifaciens* O5 (PAO5) (Fig. 4, *A*–*C*), which expresses the blood group–like αGal antigen. Gal-9, Gal-9N, and Gal-9C also bound *Klebsiella pneumoniae* O1 (KPO1) (Fig. 4, *A* and *I*), which expresses a very similar glycan determinant as that which occurs on the surface of PAO5. These interactions appeared to reflect unique strain-specific recognition of the O antigens of KPO1 and PAO5, as similar binding was not observed toward related strains that do not express these same antigen determinants (*K. pneumoniae* O4 [KPO4] and *P. alcalifaciens* O19 [PAO19]) (Fig. 4, *A*, *B*, *M*, and *N*).

**Figure 5:**
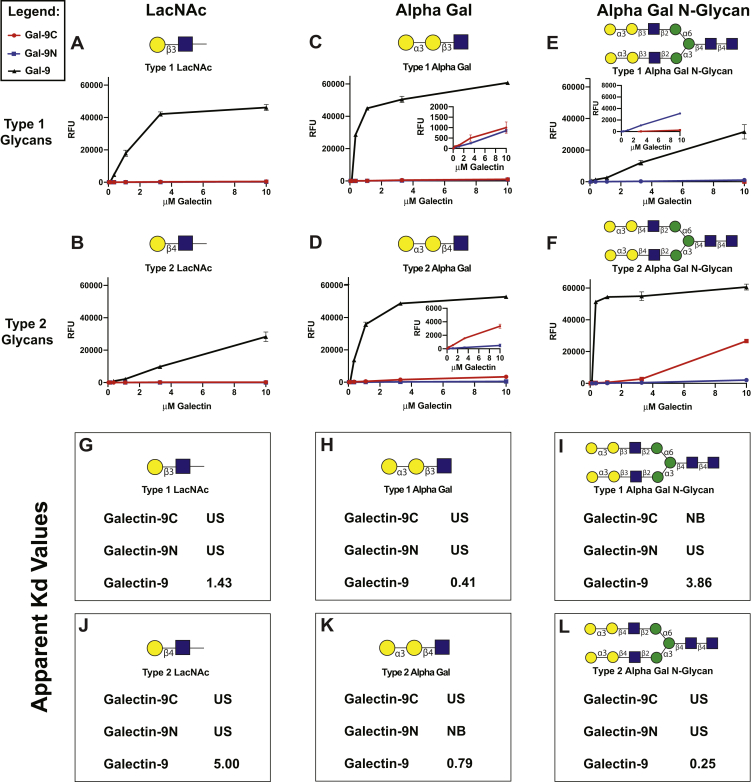
**Binding to LacNAc and αGal structures on CFG glycan microarray by galectin-9 (Gal-9) and its domains.***A* and *B*, structures of tested LacNAc glycans, (*C* and *D*) αGal glycans, and (*E* and *F*) αGal *N*-glycans are shown above dose responses of Gal-9 (*black*), Gal-9N (*blue*), and Gal-9C (*red*) binding to corresponding antigens at varying galectin concentrations. Binding strength of microbial glycans to each galectin was measured in relative fluorescence units (RFUs). *G*–*L*, table of apparent *K*_*d*_ values. US = *K*_*d*_ could not be calculated because of unsaturated galectin–glycan binding. NB = no binding (galectin–glycan binding was not detectable). Error bars represent standard deviation among six replicates. CFG, Consortium for Functional Glycomics; Gal-9C, C-terminal domain of Gal-9; Gal-9N, N-terminal domain of Gal-9; LacNAc, *N*-acetyl-lactosamine.

The binding of Gal-9, Gal-9N, and Gal-9C toward *K. pneumoniae* and *P. alcalifaciens* suggests that Gal-9 and its domains may possess a strong and perhaps similar affinity toward the αGal antigen. To test this, we examined the binding of Gal-9 and its domains toward a defined set of αGal antigen–containing glycans represented on the CFG array (Fig. 5). Similar to the binding observed toward A, B, and H blood group antigens, examination of αGal-containing glycans suggested that the substructures on which the αGal antigen is presented can influence overall glycan recognition. While Gal-9 readily recognizes all αGal antigens, it showed the highest affinity for type 2 αGal presented as an N glycan (Fig. 5, *F* and *L*). However, Gal-9 preferentially binds to type 1 αGal as a trisaccharide rather than as an N-glycan (Fig. 5, *C*, *E*, *H*, and *I*). The individual Gal-9 domains show similar discretion in binding preferences for αGal antigens based on substructure presentation. For example, Gal-9C appeared to exhibit higher binding toward type 2 αGal over a range of concentrations when presented in the context of an N glycan compared with when presented as a trisaccharide (Fig. 5, *D*, *F*, *K*, and *L*). These results once again suggest that terminal glycan presentation of blood group antigens and similar mammalian-like structures (αGal) can influence the overall binding by Gal-9 and each of its domains in a distinct manner.

### Gal-9, Gal-9N, and Gal-9C specifically recognize and kill distinct strains of *K. pneumoniae* and *P. alcalifaciens*

**Figure 6:**
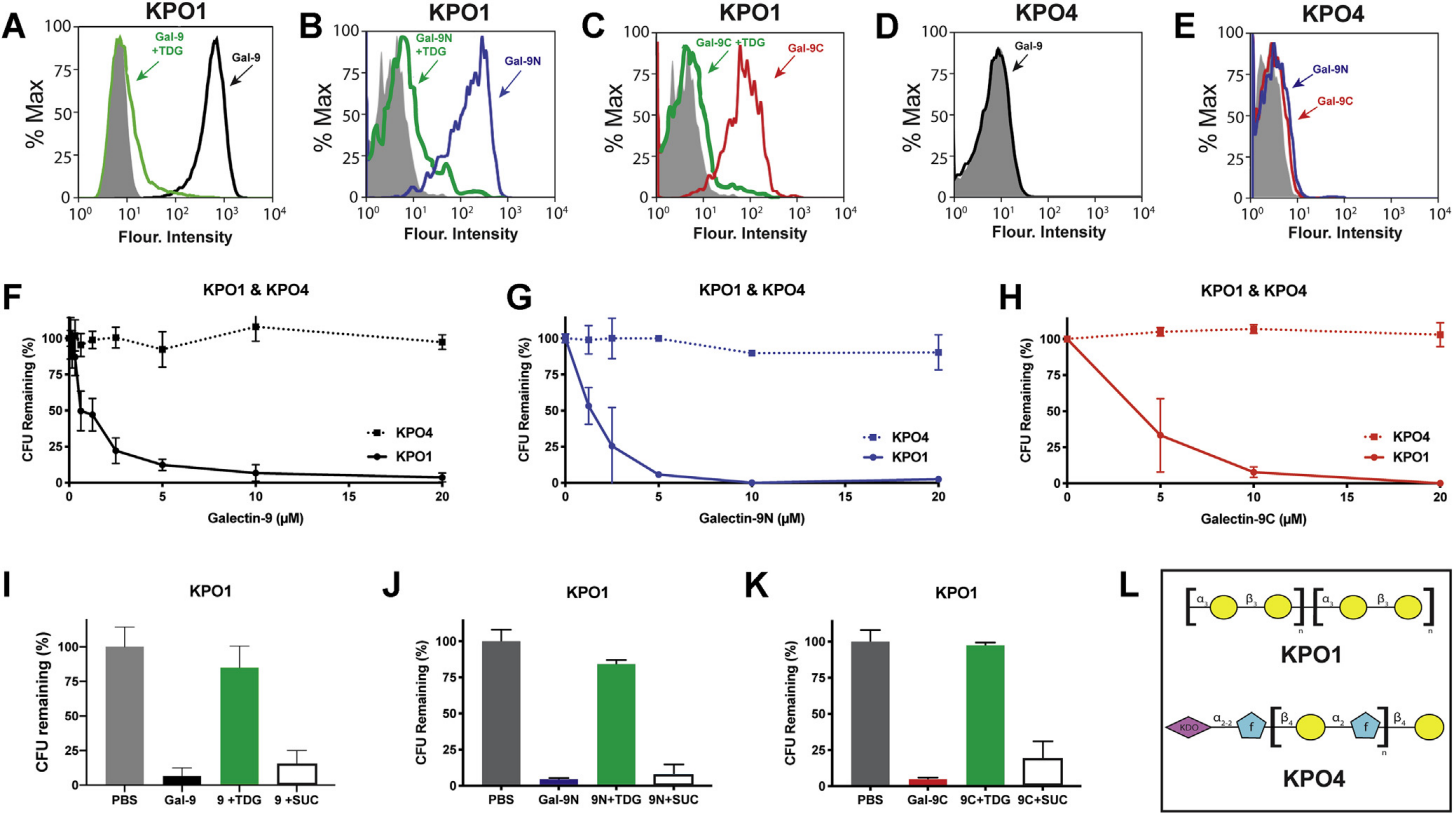
**Galectin-9 (Gal-9) recognizes and kills *Klebsiella pneumoniae* O1 (KPO1).***A*–*C*, flow cytometric analysis of KPO1 binding after incubation with Gal-9 (*A*, *black*), Gal-9N (*B*, *blue*), or Gal-9C (*C*, *red*) with or without 20 mM thiodigalactoside (TDG) (*A*–*C*, *green*) or streptavidin alone as a staining control (*A*–*C*, *solid gray*). *D* and *E*, flow cytometric analysis of *K. pneumoniae* O4 (KPO4) binding after incubation with Gal-9 (*D*, *black*), Gal-9N (*E*, *blue*), and Gal-9C (*E*, *red*). *F*–*H*, CFU quantification of KPO1 or KPO4 after incubation with varying concentrations of Gal-9 (*F*), Gal-9N (*G*), or Gal-9C (*H*). *I*–*K*, CFU quantification of KPO1 after incubation with PBS control, 10 μM Gal-9 (*I*), Gal-9N (*J*), or Gal-9C (*K*) with or without 20 mM TDG or 20 mM sucrose as controls. *L*, structures of KPO1 and KPO4 microbial glycans. Error bars represent standard deviation among three experimental replicates. Results are representative of three independent experiments. CFU, colony-forming unit; Gal-9C, C-terminal domain of Gal-9; Gal-9N, N-terminal domain of Gal-9.

The ability of the MGM to more accurately predict the potential of Gal-9, Gal-9N, and Gal-9C to kill BgB+ *E. coli* suggests that interactions observed on the MGM may likewise predict antimicrobial activity of Gal-9 and its domains toward other microbes. To examine this possibility, we first studied potential interactions between Gal-9 and its domains with KPO1 (Fig. 6). Incubation of Gal-9, Gal-9N, and Gal-9C with KPO1 resulted in detectable interactions as measured by flow cytometry (Fig. 6, *A*–*C*). To determine whether these interactions required carbohydrate recognition, Gal-9, Gal-9N, and Gal-9C were incubated with KPO1 in the presence of TDG. Similar to the ability of TDG to inhibit interactions between each domain and BgB+ *E. coli*, TDG likewise inhibited binding between Gal-9 and each domain to KPO1, strongly suggesting that these interactions required carbohydrate recognition (Fig. 6, *A*–*C*). Recognition of KPO1 by Gal-9, Gal-9N, and Gal-9C also appeared to be specific to the unique O antigen determinant of KPO1, as similar interactions failed to occur following incubation of Gal-9, Gal-9N, and Gal-9C with KPO4 (Fig. 6, *D* and *E*). To determine whether Gal-9, Gal-9N, or Gal-9C possess antimicrobial activity, especially given the relative inability of Gal-9N to kill BgB+ *E. coli*, we next examined the antimicrobial activity of Gal-9 and each of its domains toward KPO1 (Fig. 6, *F*–*H*). Similar to the general binding patterns observed on the MGM, Gal-9 was the most potent, with an EC_50_ around 0.75 μM (Fig. 6*F*). In contrast, the EC_50_ for Gal-9N was around 1.25 μM (Fig. 6*G*). Gal-9C, which exhibited the lowest binding toward the isolated glycan of KPO1, likewise displayed the lowest EC_50_ (5 μM) (Fig. 6*H*), suggesting the MGM array analysis can in general provide a relative prediction of antimicrobial potency. Consistent with the lack of binding observed toward KPO4, Gal-9, Gal-9N, and Gal-9C failed to kill KPO4 (Fig. 6, *F*–*H*), strongly suggesting that binding and killing of KPO1 required the unique glycans expressed on this strain of *K. pneumoniae*. Similar to binding, the killing activity observed by Gal-9 and each domain was also inhibited by TDG inclusion even when examined at the higher concentrations tested for each of the three proteins (Fig. 6, *I*–*K*), once again suggesting that binding and killing of this strain requires strain-specific glycan recognition. Taken together, these results demonstrate that Gal-9 possesses antimicrobial activity toward KPO1 through both domains.

**Figure 7:**
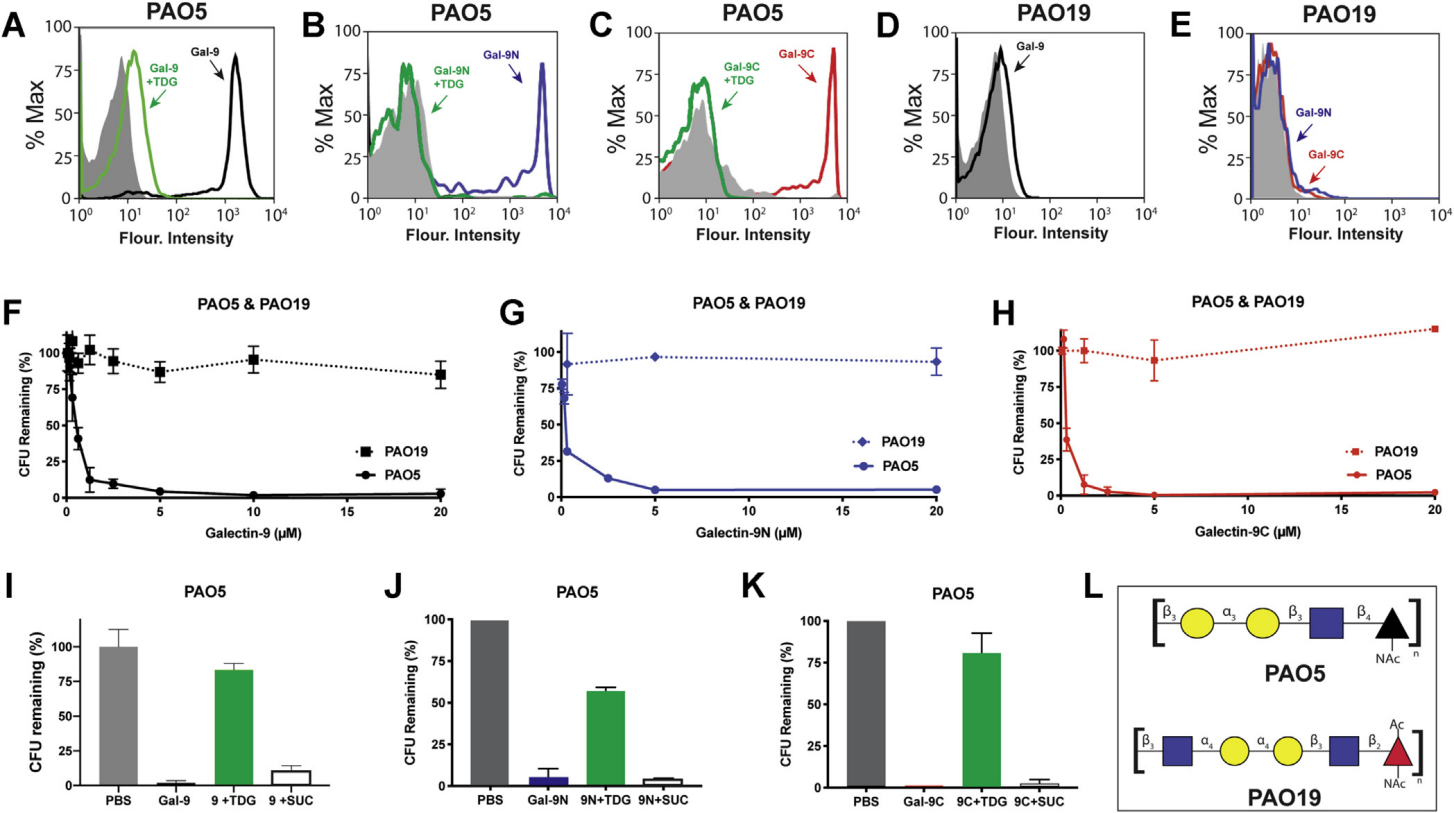
**Galectin-9 (Gal-9) recognizes and kills *Providencia alcalifaciens* O5 (PAO5).***A*–*C*, flow cytometric analysis of PAO5 binding after incubation with Gal-9 (*A*, *black*), Gal-9N (*B*, *blue*), or Gal-9C (*C*, *red*) with or without 20 mM thiodigalactoside (TDG) (*A*–*C*, *green*) or streptavidin as a staining control (*A*–*C*, *solid gray*). *D* and *E*, flow cytometric analysis of *P. alcalifaciens* O19 (PAO19) binding after incubation with Gal-9 (*D*, *black*), Gal-9N (*E*, *blue*), and Gal-9C (*E*, *red*). *F*–*H*, CFU quantification of PAO5 or PAO19 after incubation with varying concentrations of Gal-9 (*F*), Gal-9N (*G*), or Gal-9C (*H*). *I*–*K*, CFU quantification of PAO5 after incubation with PBS control, 10 μM Gal-9 (*I*), Gal-9N (*J*), or Gal-9C (*K*) with or without 20 mM TDG and 20 mM sucrose as controls. *L*, structures of PAO5 and PAO19 microbial glycans. Error bars represent standard deviation among three experimental replicates. Results are representative of three independent experiments. CFU, colony-forming unit; Gal-9C, C-terminal domain of Gal-9; Gal-9N, N-terminal domain of Gal-9.

Given the ability of the MGM to accurately predict actual interactions with KPO1, we next sought to determine whether results obtained from the MGM could similarly predict actual interactions with *P. alcalifaciens* (Fig. 7). Similar to the binding observed toward PAO5 on the array, Gal-9, Gal-9N, and Gal-9C each bound PAO5 (Fig. 7, *A*–*C*). Inclusion of TDG inhibited these interactions, strongly suggesting that like binding to BgB+ *E. coli* and KPO1, these interactions require carbohydrate recognition (Fig. 7, *A*–*C*). Engagement of PAO5 also appeared to rely on the unique O antigen determinant expressed on this strain, as similar interactions were not detected following incubation with PAO19 (Fig. 7, *D* and *E*). To determine whether binding actually results in microbial killing, we next incubated Gal-9, Gal-9N, or Gal-9C with PAO5 (Fig. 7, *F*–*H*). Unlike the differences in apparent affinity of Gal-9, Gal-9N, and Gal-9C for KPO1 observed on the MGM, Gal-9 and each domain bound isolated glycans of PAO5 on the MGM with similar potency. To determine whether the similarity in binding predicts antimicrobial potential, the killing activity of Gal-9 and each domain was assessed over several concentrations toward PAO5. Similar to the binding observed on the array, Gal-9 and each domain exhibited similar EC_50_ killing activity toward PAO5 (Fig. 7, *F*–*H*). In contrast, Gal-9, Gal-9N, and Gal-9C failed to kill the related stain of *P. alcalifaciens* that does not express the αGal antigen, PAO19 (Fig. 7, *F*–*H*). In addition, the killing activity displayed by Gal-9 and each domain examined at the higher concentrations tested was inhibited by TDG (Fig. 7, *I*–*K*), strongly suggesting that binding and killing require recognition of strain-specific mammalian-like microbial glycans. These results strongly suggest that Gal-9, Gal-9N, and Gal-9C possess the ability to bind and kill multiple microbes that express the αGal antigen.

### Gal-9, Gal-9N, and Gal-9C bind RBCs without inducing loss of membrane integrity

**Figure 8:**
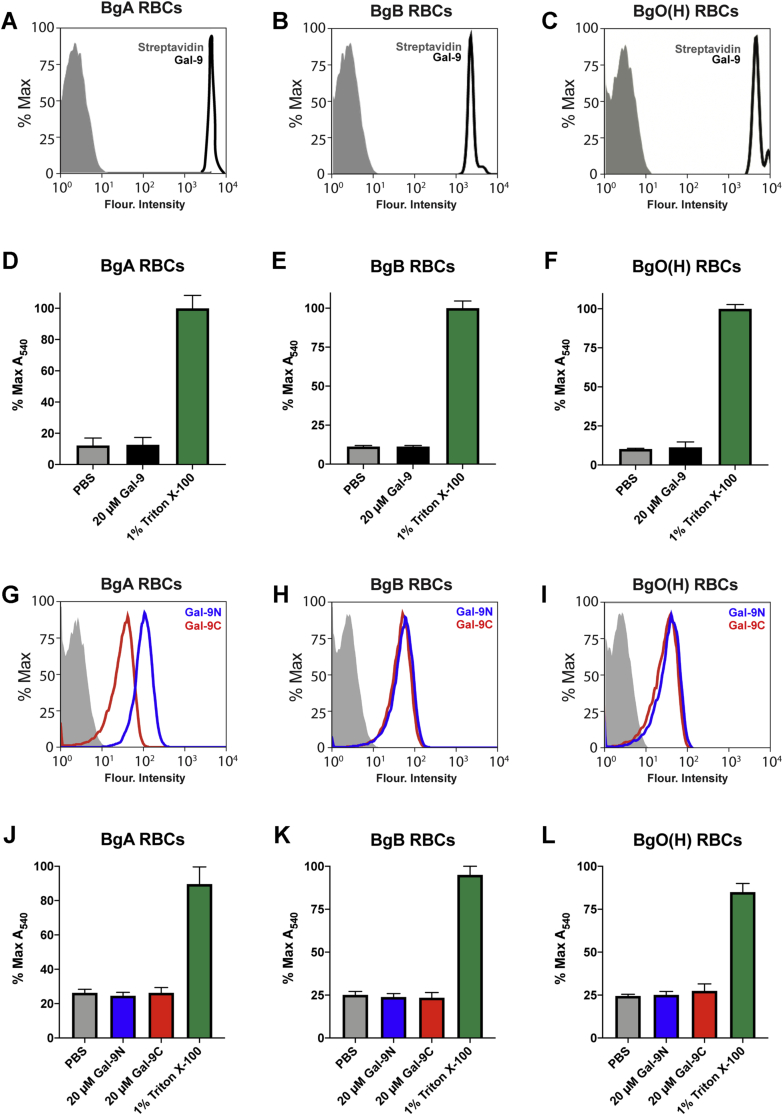
**Galectin-9 (Gal-9) binds red blood cells (RBCs) without inducing cell lysis.***A*–*C*, flow cytometric analysis of Gal-9 (*black*) or streptavidin control (*solid gray*) binding to RBCs isolated from blood group A (BgA; *A*), blood group B (BgB; *B*), or blood group O(H) (BgO(H); *C*) individuals. *D*–*F*, relative hemolysis of BgA RBCs (*D*), BgB RBCs (*E*), and BgO(H) RBCs (*F*) treated with Gal-9, PBS, or 1% Triton X-100. *G*–*I*, flow cytometric analysis of Gal-9N (*blue*), Gal-9C (*red*), or streptavidin control (*solid gray*) binding to RBCs isolated from BgA (*G*), BgB (*H*), or BgO(H) (*I*) individuals. *J*–*L*, relative hemolysis of BgA RBCs (*J*), BgB RBCs (*K*), and BgO(H) RBCs (*L*) treated with Gal-9N, Gal-9C, PBS, or 1% Triton X-100. Error bars represent standard deviation among three biological replicates. Results are representative of three independent experiments.

The ability to bind and kill microbes that decorate themselves in self-like antigens reflects a unique activity shared by several galectin family members that stands in stark contrast to most innate and adaptive immune factors, which target microbes by recognizing molecular motifs that are unique to potential pathogens (27). To determine the overall ability of Gal-9, Gal-9N, and Gal-9C to bind host cells in addition to the consequences of these potential interactions, we next examined binding activity of Gal-9 and each domain toward RBCs (Fig. 8). Incubation of Gal-9, Gal-9N, and Gal-9C with RBCs demonstrated that though Gal-9 readily bound RBCs of blood types A, B, and O(H) (Fig. 8, *A*–*C*), individual domains exhibited clear binding preferences. Gal-9N displayed slightly higher binding activity than Gal-9C toward RBCs isolated from blood group A individuals (Fig. 8*G*). In contrast, each domain exhibited similar binding preferences toward RBCs from BgB or O individuals (Fig. 8, *H* and *I*). While Gal-9, Gal-9N, and Gal-9C readily killed blood group–expressing BgB+ *E. coli*, KPO1, and PAO5, no detectable change in membrane integrity was observed following incubation of RBCs isolated from all blood group types (Fig. 8, *D*–*F* and *J*–*L*). Taken together, these results suggest that Gal-9 and its domains possess the ability to recognize mammalian and microbial surface antigens but only induce death of microbes.

## Discussion

While Gal-9 bound a variety of glycans when analyzed on the CFG glycan microarray, blood group antigens appeared to be among the highest affinity glycans not only for the full-length protein but likewise for the individual domains. However, the subtle impact of ABO(H) substructure on glycan recognition demonstrates that minor glycan modifications can influence carbohydrate recognition in a domain-specific manner, providing important insight into unique glycan-binding profiles of Gal-9 and each of its domains. The distinct binding preferences observed toward unique ABO(H) and related glycan determinants were not only observed on glycan arrays but also when examined toward intact microbes, illustrating the utility of a combined approach of glycan microarray analysis and investigation of intact microbes when seeking to define the binding specificity of galectins. This approach uncovered specific interactions between Gal-9 and its individual domains with microbes. In doing so, each domain was found to possess antimicrobial activity against distinct microbial strains. These results demonstrate that in addition to the full-length protein, the individual domains of Gal-9 also possess the ability to specifically recognize and kill microbes expressing self-like antigens and in so doing may contribute to innate immunity against molecular mimicry.

Previous studies have employed a variety of approaches when seeking to define the binding specificity of lectins in general, and galectins in particular, toward carbohydrate ligands. Owing to the synthetic complexity of glycan biosynthesis, early analysis of protein–carbohydrate interactions was often limited in the number of glycan determinants against which possible interactions could be ascertained (28, 29, 30). Even when larger glycan libraries materialized (31, 32, 33), many approaches required larger amounts of lectins or carbohydrates to establish possible interactions, often prohibiting a higher throughput approach when seeking to define galectin–glycan specificity (31, 32, 33). Notwithstanding these challenges, previous studies provided important understanding regarding the binding specificity of galectins (23, 34, 35, 36). However, the use of glycan microarray technology in particular has greatly expanded the ability to assess lectin–glycan interactions as this approach has allowed for large libraries of immobilized glycans to be interrogated with small amounts of lectins (37, 38, 39, 40).

Glycans immobilized in a microarray format may not only facilitate lectin analysis but also this approach may orient this interaction in a way that allows probing of bound glycans as occurs on a cell surface, where the limitations in glycan flexibility, when the reducing end of the sugar is immobilized, may not be as apparent when glycans are free in solution (21, 22). The ability of the MGM to accurately predict actual interactions with intact microbes likewise suggests the potential utility of this approach, as MGM data appeared to predict general features of galectin interactions with microbes not previously recognized as possible Gal-9 targets. However, it is important to note that glycan presentation in an array format may influence the overall apparent affinity for a given glycan. For example, while the relative binding patterns observed for Gal-9, Gal-9N, and Gal-9C toward blood group antigens on the CFG glycan microarray and the ABH array were relatively similar, the apparent affinity significantly differed. Unlike the CFG array, the ABH array was assembled using neoglycoproteins as opposed to direct glycan printing in an array format. Unique features of glycan presentation on each array, including the density of the presented glycan or other features altogether, may account for some of these differences in apparent affinity. However, as glycan arrays are primarily screening tools used to define the relative affinities of carbohydrate-binding proteins for a library of glycans, the relative differences in binding, as opposed to the apparent affinity, are likely more important. In this way, glycan microarrays can be tremendously informative regarding glycan features that may increase or inhibit carbohydrate recognition. Such findings can then inform cell-based studies that ultimately seek to define how changes in cell surface glycosylation may impact glycan recognition (41).

While fewer studies have examined the fine carbohydrate-binding specificity of Gal-9 toward glycans (42, 43, 44), in particular toward blood group antigens, prior studies using frontal affinity chromatography demonstrated that Gal-9 has affinity for branched N-glycans, repeated oligolactosamines, and glycolipid-type glycans (36). Examination of glycolipids in the context of cell surface glycans also demonstrated that in contrast to Gal-4 and Gal-8, which appeared to prefer blood group A glycans, Gal-9 displayed higher binding toward BgB glycans (44). Subgroup analysis did reveal higher binding toward type 2 BgB glycans, than type 1 or type 3 (44). Additional studies suggested that similar to Gal-4 and Gal-8, blood group presentation on LacNAc enhances Gal-9 recognition, with blood group A or BgB modifications producing a similar increase in overall Gal-9 binding (42). Gal-9N displayed similar and distinct binding profiles toward blood group antigens when compared with other galectins (42). Additional crystallographic and NMR spectroscopy data of Gal-9N with lactose and Forsmann disaccharide (GalNAcα1–3GalNAc) provided fine detailed analysis of glycan interactions with the CRD of Gal-9N and glycan ligands (43). Taken together, these prior studies demonstrated that Gal-9 can recognize blood group and blood group–like glycans.

Consistent with previous findings (22, 35, 42, 44), the data presented here suggest that simple additions of blood group and blood group–like determinants to LacNAc can impact Gal-9 glycan interactions. While full-length Gal-9 exhibited high binding to blood group antigens in general, the addition of α1–2 fucose and α1–3 GalNAc (blood group A) or Gal (BgB) favorably modulated this interaction within each domain. Previous studies suggested that the Gal–GlcNAc linkage can influence blood group recognition, despite very little, if any, binding detected toward type 1 or two LacNAc alone when examined in the same format (22, 44). Several previous studies also demonstrated that Gal-9 type 1 or 2 LacNAc can influence glycan recognition (19). Similar to the present studies, general preferences of Gal-9 for polyLacNAc containing glycans have also been reported (24). Alterations in specific interactions with the core glycan structure, including GlcNAc, GalNAc, or Glc, may be particularly apparent once additional interactions with the α1–2 fucose and either α1–3 GalNAc or α1–3Gal are realized. However, it should be noted that not all galectin–blood group antigen interactions appear to be influenced in this way, as the impact of LacNAc linkages or the presentation of blood group antigens on different core structures does not appear to be a common feature shared among all galectin family members (21). The distinct influence of additional binding pockets on the CRD surface may be responsible for conveying some level of specificity toward core structures in the context of ABO(H) glycan recognition, as suggested for galectin recognition of other glycans (35). This fine tuning of glycan specificity may allow individual domains of galectins to recognize distinct biological targets, including specific microbes. It should be noted that while previous studies using microarrays populated with microbial glycans failed to identify blood group–positive microbial targets of Gal-9 (18), the results here suggest that Gal-9 is capable of recognizing and killing BgB+ *E. coli*. While the underlying reason for these differences remains unknown, possible differences in LPS isolation, array construction, or other differences altogether may account for some degree of variation between the present findings and previous studies.

Previous glycan array studies indicate that extended LacNAc structures are preferred glycans recognized by Gal-9, and that Gal-9 binding can be enhanced with terminal LacNAc modifications (19, 24). Consistent with these reports, we observed that extension of LacNAc structures enhances Gal-9 binding when compared with LacNAc alone. Using Gal-9 binding to LacNAc as a baseline for comparison, we also found that the addition of blood group A or BgB modifications to LacNAc significantly enhances Gal-9 binding. Among all glycans tested on the CFG array, N-glycans with terminal blood group or extended LacNAc structures were some of the highest affinity Gal-9 ligands. At the same time, Gal-9 bound to type 2 H antigen with less affinity when compared with related blood group structures, consistent with the findings from previous glycan array studies, demonstrating that H-antigen terminal modifications can decrease Gal-9 recognition (24).

While Gal-9 likewise bound with high affinity toward blood group antigen terminating N glycans (similar to prior studies demonstrating the influence of N glycans on galectin interactions (43)), presentation of the blood group antigen on N glycans also appears to modulate the overall binding affinity of each domain toward blood group antigens. Given our observations that confirm previous reports of the enhanced binding of each domain toward LacNAc when presented on N glycans when compared with LacNAc alone (19, 24), differences in blood group recognition when presented on N glycans may reflect a slight preference for N glycans in general irrespective of LacNAc modifications. Galectins likely evolved the ability to recognize LacNAc in the context of N glycans, O glycans, glycolipids, and perhaps other cell surface glycans, such as microbial glycans (45). Recognition of LacNAc-bearing motifs in this manner is thought to mediate many galectin activities, including the induction and regulation of signaling events that regulate cell behavior (20, 23, 46, 47). Although the concentration of galectins can vary depending on the tissue type, activation state, and overall level of galectin secreted in a given environment, the ability of Gal-9 to achieve appreciable binding toward common leukocyte ligands, such as polyLacNAc, in addition to blood group antigens, over a similar concentration range may reflect a dual role for Gal-9 and perhaps other galectins in directly targeting microbes while also providing a galectin-mediated regulation of leukocyte function. Indeed, the effective concentration required for Gal-9-mediated antimicrobial activity in the present study is similar to range of concentrations used to evaluate Gal-9 activity in other settings (48, 49, 50, 51), suggesting that Gal-9 (and likely other galectins) possess the unique ability to engage a variety of glycan motifs with distinct outcomes depending on the cell type engaged. The higher binding of Gal-9N and Gal-9C toward ABO(H) antigens when presented on N glycans therefore likely reflects a general underlying preference of Gal-9N and Gal-9C for N glycans that can be influenced by ABO(H) antigen expression. However, given the polymorphic nature of ABO(H) expression, it is unlikely that specific interactions between Gal-9 and ABO(H) structures as presented on N glycans are responsible for fundamental regulation of cellular processes. Instead, this binding outcome likely reflects separate ABO(H) and N glycan–binding preferences, which when presented in the same context enhance recognition.

The selective pressures that drove blood group recognition by galectins and the expression of ABO(H) antigens themselves within the human population remain relatively unknown. Although Gal-9 recognized polyLacNAc structures with similar overall apparent affinity on the CFG glycan microarray, blood group antigens were some of the most prominent glycans recognized by Gal-9, Gal-9N, and Gal-9C. These results suggest that similar to Gal-4 and Gal-8, Gal-9 exhibits high-affinity interactions with blood group antigens. Several hypotheses exist regarding the evolution of ABO(H) antigens, including their involvement in infectious disease modulation (52, 53, 54, 55). The relative inability of blood group–positive individuals to generate antiblood group antibodies would also be predicted to come at a fitness cost with respect to an individual's ability to kill blood group–positive microbes. The ability of Gal-9 and perhaps other galectins to recognize and kill blood group–positive microbes suggests that innate immune factors may fill this gap in adaptive immunity by directly killing microbes that utilize molecular mimicry. However, whether galectins possessed the ability to recognize blood group antigens prior to the evolution of ABO(H) polymorphisms and therefore possibly facilitate the selection of these polymorphisms remains unknown.

The ability of Gal-9, Gal-9N, and Gal-9C to bind RBCs and microbes, but only kill microbes, is unique among immune factors, which primarily engage microbes through recognition of microbe-specific molecular motifs (27). RBCs and other host cells express carbohydrate antigens, which like microbes, decorate the cell surface. However, unlike microbes, RBCs and other host cells express a variety of carbohydrate antigens, which can include not only blood group antigens but also many other glycan determinants that may serve as ligands for physiological responses induced by galectins (56, 57). As a result, assessing overall binding profiles on a host cell surface and attributing the binding strength to any one class of glycan can be challenging in the absence of glycoengineering approaches designed to produce more uniform and predictable cell surface glycosylation (41). Thus, possible differences in overall binding by each domain on the surface of RBCs may be influenced by a variety of glycoforms, including blood group antigens, possibly recognized by individual Gal-9 domains. Prokaryotes often produce repeating units of similar glycan structures (58), creating situations where binding may be more easily predicted by results obtained from the use of microarrays populated with prokaryote-derived microbial glycans. The distinct outcome of cell surface recognition observed following incubation of Gal-9, Gal-9N, or Gal-9C with mammalian cells or microbes suggests that Gal-9 possesses the unique ability to engage the surface of two distinct cells with very different outcomes. Such an ability would be predicted to be a key trait of an immune factor that seeks to specifically identify and kill microbes that appear to decorate themselves with self-like glycans as a form of molecular mimicry.

Collective binding by individual domains may result in the enhanced affinity observed for the full-length protein; increases in the effective concentration of one domain may facilitate additional interactions by the other domain, thus enhancing overall interactions with target glycans. Despite this, the individual domains did possess considerable antimicrobial activity, even when assessed at concentrations considered to be physiologic and therefore commonly employed to examine galectin activity. The ability of each domain to intrinsically bind and kill microbes may be especially important when considering that several proteases have been shown to cleave the linker protein responsible for tethering individual CRDs (59). While this strategy may have evolved as a feedback loop to modulate galectin-regulated immune circuits that require the full-length protein, the ability of each independent domain to retain antimicrobial activity suggests that despite possible cleavage events, individual domains retain the ability to exert antimicrobial activity. In this way, galectin regulation of leukocyte function and antimicrobial activity may be separately influenced.

These collective results suggest that several members of the galectin family appear to engage in distinct interactions with microbial glycans that possess blood group antigens and related mammalian-like features. The binding preferences observed for Gal-9, Gal-9N, and Gal-9C toward microbial glycans, while exhibiting similarities and distinct differences, suggest that as a whole, the collective binding profile of individual galectins may target a broad range of microbes. Consistent with this, Gal-9N and Gal-9C displayed overlapping and unique binding characteristics toward the microbial antigens represented on the MGM. Unfortunately, many of the microbes represented on the MGM are not readily available, precluding additional confirmatory studies with intact microbes that would be predicted to be bound and possibly killed based on Gal-9-binding, Gal-9N-binding, or Gal-9C-binding outcomes. However, analysis of available microbes, including BgB+ *E. coli*, KPO1, PAO5, KPO4, and PAO19, suggested that at least for these microbial strains, array results accurately predicted antimicrobial activity. These results suggest that individual galectin family members and even domains within a galectin may target a variety of microbes, each of which may share general features of mammalian glycans.

## Experimental procedures

### Galectin growth and purification

Gal-9, Gal-9N, and Gal-9C were expressed using the following sequences:

#### Gal-9

MAFSGSQAPYLSPAVPFSGTIQGGLQDGLQITVNGTVLSSSGTRFAVNFQTGFSGNDIAFHFNPRFEDGGYVVCNTRQNGSWGPEERKTHMPFQKGMPFDLCFLVQSSDFKVMVNGILFVQYFHRVPFHRVDTISVNGSVQLSYISFQTPAIPPMMYPHPAYPMPFITTILGGLYPSKSILLSGTVLPSAQRFHINLCSGNHIAFHLNPRFDENAVVRNTQIDNSWGSEERSLPRKMPFVRGQSFSVWILCEAHCLKVAVDGQHLFEYYHRLRNLPTINRLEVGGDIQLTHVQT

#### Gal-9N

MAFSGSQAPYLSPAVPFSGTIQGGLQDGLQITVNGTVLSSSGTRFAVNFQTGFSGNDIAFHFNPRFEDGGYVVCNTRQNGSWGPEERKTHMPFQKGMPFDLCFLVQSSDFKVMVNGILFVQYFHRVPFHRVDTISVNGSVQLSYISFQ

#### Gal-9C

TPAIPPMMYPHPAYPMPFITTILGGLYPSKSILLSGTVLPSAQRFHINLCSGNHIAFHLNPRFDENAVVRNTQIDNSWGSEERSLPRKMPFVRGQSFSVWILCEAHCLKVAVDGQHLFEYYHRLRNLPTINRLEVGGDIQLTHVQT

##### Gal-9 growth and purification

Expression plasmid encoding human full-length Gal-9 was transformed into *E. coli* BL21 (DE3) and was then expressed as outlined previously (60). Briefly, transformed bacteria were cultured in LB broth containing 100 μg/ml ampicillin with shaking at 250 rpm and incubated at 37 °C. When bacteria were grown to the midlogarithmic phase, protein expression was induced by addition of 0.1 M IPTG. After 20 h induction at 16 °C, 6 l of cultured bacteria were pelleted and harvested by centrifugation and then resuspended in 60 ml bacterial lysis buffer (PBS with 14 mM β-mercaptoethanol (β-ME), 60 μl ribonuclease A, 60 μl DNase I, 60 μl lysozyme, and four protease inhibitor cocktail tablets). An additional sonication step was performed for Gal-9, and final concentration with 1% Triton X-100 was added into the solution. A magnetic stir bar was added to the bacterial lysate and stirred for 30 min on a stir plate at 150 rpm at 4 °C. The lysate was centrifuged at 20,000*g* for 30 min at 4 °C twice. Supernatant was applied to lactosyl-sepharose affinity chromatography column. For elution, the elution buffer (PBS with 14 mM 2-ME and 200 mM lactose) was added. The desired fractions were pooled and stained with Coomassie blue on SDS-PAGE gel to test protein purity (Fig. 1*B*). Before derivatization, lactose and β-ME were removed from galectin solution using a PD-10 gel filtration column (GE Healthcare). Average yield of Gal-9 was about 0.5 mg per liter of transformed bacteria.

##### Gal-9N and Gal-9C growth and purification

Gal-9N and Gal-9C were expressed in *E. coli* strain Rosetta 2 (Merck Millipore). A starter culture of the transformed *E. coli* was created by inoculating 40 ml of LB media containing ampicillin (100 μg/ml) and incubating overnight at 37 °C and shaking at 250 rpm as outlined previously (20, 22, 61). About 10 ml of the culture was then added to 1 L flasks of LB media. Flasks were then incubated at 37 °C while shaking at 250 rpm. Once midlogarithmic phase was achieved (absorbance at 600 nm = 0.45–0.50), IPTG was added to each flask of Gal-9N at a final concentration of 0.1 mM and to each flask of Gal-9C at a final concentration of 1 mM. The flasks were then incubated while shaking at 250 rpm. Gal-9C flasks were incubated at 37 °C for 4 to 5 h, whereas Gal-9N was incubated for 20 h at 15 °C. The cultures were subsequently centrifuged at 4200*g* for 30 min at 4 °C. The supernatant was removed, and pellets were stored in −80 °C. Pellets were thawed on ice the following day and resuspended in 10 ml of lysis buffer per liter of starting LB volume (for 40 ml of lysis buffer: 35.3 ml Cell Lytic B-II Bacterial Cell Lysis/Extraction reagent [Sigma], 44.8 μl β-ME, four Complete Mini EDTA-free Protease Inhibitor cocktail tablets, 4 ml lysozyme, 40 μl RNase, and 40 μl DNase). The suspension was incubated for 15 min at 37 °C, while shaking at 250 rpm. The lysate was then centrifuged at 13,000*g* for 30 min at 4 °C, and the supernatant was collected. Average yield of Gal-9N and Gal-9C was about 10 to 30 mg per liter of transformed bacteria.

Recombinant galectin was then purified from the supernatant by affinity chromatography on lactosyl-sepharose. The protein was eluted with 100 mM lactose plus 14 mM β-ME in PBS to apparent homogeneity as evident on SDS-PAGE. Before derivatization, lactose and β-ME were removed from galectin solution with a PD-10 gel filtration column.

### Galectin labeling for flow cytometry and microarray analysis

Galectins were prepared for biotinylation and labeling by first adding 100 mM lactose to preserve structural integrity and activity during the reaction. Galectin biotinylation was achieved by incubating 3 to 5 mg/ml of galectin with 2 mM EZ-link Sulfo-NHS-LC-Biotin (sulfosuccinimidyl-6-(biotin-amido) hexanoate) (Pierce) for 2 h at 4 ^o^C. Any unconjugated EZ-link Sulfo-NHS-LC-Biotin and free lactose were removed from derivatized protein with a PD-10 gel filtration column. Biotinylated galectin was then stored at 4 ^o^C in 14 mM β-ME in PBS. Alexa Fluor 647 (Molecular Probes, Thermo Fisher Scientific)-labeled galectins were prepared using Alexa Fluor 647 carboxylic acid, succinimidyl ester, dilithium salt-reactive dyes (Molecular Probes, Thermo Fisher Scientific) as described previously (22). Alexa Fluor 647-labeled galectins were also stored at 4 ^o^C in 14 mM β-ME in PBS.

### Glycan microarrays

#### CFG array

Glycan microarrays were obtained from the National Institutes of Health/National Institute of General Medical Sciences–funded CFG array (version 3.0). Galectins and slides were prepared as described previously (22). Slides were incubated for 1 h in a dark humidity chamber at room temperature with varying concentrations of Gal-9, Gal-9C, or Gal-9N directly labeled with Alexa Fluor 647. Varying concentrations of galectin were prepared by dilution in 1× PBS + 0.05% Tween-20 (PBS-T). Slides were then washed four times in each PBS-T, 1 time in PBS, and distilled water. Fluorescence images were obtained using a microarray scanner (Scan Array Express; PerkinElmer Life Sciences), and integrated spot intensities were measured using Metamorph software (Universal Imaging) (31). Raw fluorescence data at each concentration were then plotted together to study binding isotherms (22, 62). Apparent *K*_*d*_ values were calculated using GraphPad Prism version 9.0.2 (GraphPad Software, LLC).

#### ABH array

ABH arrays were generated as outlined previously (63). For galectin recognition of glycans on the printed glycan microarray, slides were incubated with varying concentrations of Gal-9, Gal-9N, or Gal-9C directly labeled with Alexa Fluor 647 in PBS-T for 1 h at room temperature in a dark and humid chamber. The slide was washed by successive immersion in PBS-T (four times), 1× PBS (four times), and distilled water (four times). The slide was dried by microcentrifugation, and an image of bound fluorescence was obtained using a microarray scanner (Scan Array Express). Integrated spot intensities were determined using Imagene software (BioDiscovery). Apparent *K*_*d*_ values were calculated using GraphPad Prism version 9.0.2 (GraphPad Software, LLC).

#### MGM array

MGMs (MGM array v5.2) were obtained from the CFG. Slides were prepared as described previously (39) and incubated with varying concentrations of Gal-9, Gal-9N and Gal-9C directly labeled with Alexa Fluor 647 and diluted in PBS-T. Slides were incubated at room temperature in a dark humidity chamber. After 1 h, slides were washed by successive immersion four times in each PBS-T, 1x PBS and distilled water. An image of bound fluorescence was obtained using a microarray scanner (Scan Array Express). Integrated spot intensities were determined using Imagene software (BioDiscovery). Raw data were also color coded by magnitude to create a heat map using Microsoft Excel. Apparent *K*_*d*_ values were calculated using GraphPad Prism version 9.0.2 (GraphPad Software, LLC).

### Whole-cell galectin-binding assay by flow cytometry

To assess galectin binding to whole-cell microbes, bacteria were grown to midlogarithmic phase in LB media at 37 ^o^C. Cells were then resuspended at a concentration of 10^8^ cells/ml in PBS with ∼0.1 μM biotinylated galectin at 4 ^o^C for 30 min. Control samples also included 20 mM of TDG. Following incubation, bacteria were washed three times in PBS and incubated with Alexa Fluor 488 streptavidin (Molecular Probes, Thermo Fisher Scientific) at 4 ^o^C for 30 min. Bacteria were washed twice more and resuspended in 300 μl of 1× PBS for analysis by flow cytometry using a FACSCalibur flow cytometer (BD Biosciences). Results were analyzed using CellQuest software (BD Biosciences) (64).

### Galectin antimicrobial assay

All bacteria were grown at 37 ^o^C in LB media (Fisher) to midlogarithmic phase (absorbance at 600 nm = 0.45–0.5). To assess dose response, varying concentrations of galectins indicated (0.03–20 μM) were incubated with 10^8^ bacteria/ml for 2 h at 37 ^o^C while shaking at 250 rpm. Control samples also included 20 mM TDG or sucrose as indicated. Viable bacteria were measured by dilution plating and colony-forming unit (CFU) quantification (13, 61).

### Galectin binding to RBCs and hemolysis assay

Binding of Gal-9, Gal-9N, and Gal-9C to RBCs was assessed by flow cytometry as outlined previously (65, 66, 67). Biotinylated galectins or streptavidin control were incubated with RBCs isolated from blood group A, BgB, or blood group O individuals. RBC hemolysis was assessed following incubation with 20 μM Gal-9, Gal-9N, Gal-9C, 1× PBS (negative control), or 1% Triton X-100 (positive control). Hemolysis was measured by spectrometer absorbance at 540 nm. Relative hemolysis of blood group A RBCs, BgB RBCs, and blood group O RBCs under each condition is reported as percentage of maximum absorbance, defined as the average absorbance of positive control (68).

## Data availability

All data are contained within the article.

## Supporting information

This article contains supporting information.

## Conflict of interest

The authors declare that they have no conflicts of interest with the contents of this article.
