# Supplementary material for: Galectin-9 recognizes and exhibits antimicrobial activity toward microbes expressing blood group–like antigens
Source: J Biol Chem. 2022 Feb 9;298(4):101704. doi: 10.1016/j.jbc.2022.101704 (PMC9019251; doi:10.1016/j.jbc.2022.101704)
Supplement: Supplemental Figures S1–S3 and Tables S1–S5 Legends [file mmc1.docx]

###### **Title:** Galectin-9 recognizes and exhibits antimicrobial activity toward microbes expressing blood group-like antigens

**Authors:** Anna V. Blenda^1,2^*†, Nourine A. Kamili^2^†, Shang-Chuen Wu^3^, William F. Abel^1^, Diyoly Ayona^3^, Christian Gerner-Smidt^2^, Alex Ho, Guy M. Benian^2^, Richard D. Cummings^4^, Connie M. Arthur^2,3^, and Sean R. Stowell^2,3^*

**List of Supporting Information:**

##### Figure S1: Comparison of Gal-9, Gal-9C and Gal-9N binding affinities on the CFG microarray

##### Figure S2: Comparison of Gal-9, Gal-9C and Gal-9N binding affinities on the microbial glycan microarray (MGM)

##### Figure S3: Apparent Kd values of Gal-9, Gal-9C and Gal-9N binding to lactosamine and blood group antigens on CFG array

##### Table S1: Table of apparent Kd values of Gal-9, Gal-9C and Gal-9N binding to glycans on CFG array (PDF)

##### Table S2: Table of apparent Kd values of Gal-9, Gal-9C and Gal-9N binding to glycans on MGM array (PDF)

##### Table S3: MGM array legend of microbial glycans (PDF)

##### Table S4: CFG Raw Data (Excel)

##### Table S5: MGM Raw Data (Excel)

#####

##### **Figure S1. Comparison of Gal-9, Gal-9C and Gal-9N binding affinities on the CFG microarray**. (A) Schematic of fluorescently labeled galectin incubated with microarray to identify positive binders. (B) Example plot of galectin binding to glycan over a range of galectin concentrations tested, generating a dose curve that is used to calculate Kd of galectin-glycan affinity. (C) Selected N-glycans containing blood group, lactosamine, Lewis or sialylated antigens on CFG array and apparent Kd values of Gal-9, Gal-9N and Gal-9C binding. (D) Selected glycans with terminal blood group, lactosamine or Lewis antigens on CFG array and apparent Kd values of Gal-9, Gal-9N and Gal-9C binding.

#####

##### **Figure S2. Comparison of Gal-9, Gal-9C and Gal-9N binding affinities on the microbial glycan microarray (MGM)**. Selected *Klebsiella*, *Providencia*, *Streptococcus* and *Proteus* microbial glycans on the MGM and associated Kd values of Gal-9, Gal-9N and Gal-9C binding to microbial glycans.

**Figure S3. Apparent Kd values of Gal-9, Gal-9C and Gal-9N binding to lactosamine and blood group antigens on CFG array**. Each glycan structure followed by its trivial name is shown above listed apparent Kd values of Gal-9, Gal-9C and Gal-9N binding to indicated lactosamine or blood group glycan structure on CFG array. Gal = galactose, GalNAc= *N*-acetylgalactosamine, Glc = glucose, GlcNAc = *N*- acetylglucosamine, Fuc = fucose, Man = mannose.
