# Supplementary material for: Galectin-9 recognizes and exhibits antimicrobial activity toward microbes expressing blood group–like antigens
Source: J Biol Chem. 2022 Feb 9;298(4):101704. doi: 10.1016/j.jbc.2022.101704 (PMC9019251; doi:10.1016/j.jbc.2022.101704)
Supplement: Supplemental Table S1 [file mmc2.pdf]

Table S1. Table of apparent Kd values of Gal-9, Gal-9C and Gal-9N binding to glycans on CFG array

NB = no binding unsat. = unsaturated binding (Kd could not be calculated)

Glycan #	Structure_on_Masterlist	Gal-9 Kd	Gal-9C Kd	Gal-9N Kd
1	Gala-Sp8	NB	NB	NB
2	Glca-Sp8	NB	NB	NB
3	Mana-Sp8	NB	NB	NB
4	GalNAca-Sp8	NB	NB	NB
5	Fuca-Sp8	NB	NB	NB
6	Fuca-Sp9	NB	NB	NB
7	Rhaa-Sp8	NB	NB	NB
8	Neu5Aca-Sp11	NB	NB	NB
9	Neu5Aca-Sp8	NB	NB	NB
10	Neu5Acb-Sp8	NB	NB	NB
11	Galb-Sp8	NB	NB	NB
12	Glcb-Sp8	NB	NB	NB
13	Manb-Sp8	NB	NB	NB
14	GalNAcb-Sp8	NB	NB	NB
15	GlcNAcb-Sp0	NB	NB	NB
16	GlcNAcb-Sp8	NB	NB	NB
17	GlcN(Gc)b-Sp8	NB	NB	NB
18	(3S)Galb1-4(6S)Glcb-Sp0	**0.67**	unsat.	unsat.
19	(3S)Galb1-4(6S)Glcb-Sp8	**0.55**	unsat.	unsat.
20	Galb1-4GlcNAcb1-6(Galb1-4GlcNAcb1-3)GalNAc-Sp14	**2.47**	NB	NB
21	Galb1-4GlcNAcb1-6(Galb1-4GlcNAcb1-3)GalNAca-Sp8	**0.84**	unsat.	NB
22	GlcNAcb1-6(GlcNAcb1-4)(GlcNAcb1-3)GlcNAc-Sp8	NB	NB	NB
23	(3S)Galb1-4(Fuca1-3)(6S)Glc-Sp0	unsat.	NB	NB
24	(3S)Galb1-4Glcb-Sp8	**0.77**	NB	unsat.
25	6S(3S)Galb1-4(6S)GlcNAcb-Sp0	NB	NB	NB
26	6S(3S)Galb1-4GlcNAcb-Sp0	NB	NB	NB
27	(3S)Galb1-3(Fuca1-4)GlcNAcb-Sp8	NB	NB	NB
28	(3S)Galb1-3GalNAca-Sp8	**0.53**	NB	unsat.
29	(3S)Galb1-3GlcNAcb-Sp0	**0.56**	unsat.	unsat.
30	(3S)Galb1-3GlcNAcb-Sp8	**0.48**	unsat.	unsat.
31	(3S)Galb1-4(Fuca1-3)GlcNAc-Sp0	NB	NB	NB
32	(3S)Galb1-4(Fuca1-3)GlcNAc-Sp8	NB	NB	NB
33	(3S)Galb1-4(6S)GlcNAcb-Sp0	**0.5**	unsat.	unsat.
34	(3S)Galb1-4(6S)GlcNAcb-Sp8	**0.46**	unsat.	unsat.
35	(3S)Galb1-4GlcNAcb-Sp0	**1.11**	unsat.	NB
36	(3S)Galb1-4GlcNAcb-Sp8	**0.84**	unsat.	NB
37	(3S)Galb-Sp8	NB	NB	NB
38	(6S)(4S)Galb1-4GlcNAcb-Sp0	NB	NB	NB
39	(4S)Galb1-4GlcNAcb-Sp8	NB	NB	NB
40	(6P)Mana-Sp8	NB	NB	NB
41	(6S)Galb1-4Glcb-Sp0	NB	NB	NB

**42**	(6S)Galb1-4Glcb-Sp8	NB	NB	NB
**43**	(6S)Galb1-4GlcNAcb-Sp8	unsat.	NB	NB
**44**	(6S)Galb1-4(6S)Glcb-Sp8	NB	NB	NB
**45**	Neu5Aca2-3(6S)Galb1-4GlcNAcb-Sp8	NB	NB	NB
**46**	(6S)GlcNAcb-Sp8	NB	NB	NB
**47**	Neu5,9Ac2a-Sp8	NB	NB	NB
**48**	Neu5,9Ac2a2-6Galb1-4GlcNAcb-Sp8	NB	NB	NB
**49**	Mana1-6(Mana1-3)Manb1-4GlcNAcb1-4GlcNAcb-Sp12	NB	NB	NB
**50**	Mana1-6(Mana1-3)Manb1-4GlcNAcb1-4GlcNAcb-Sp13	NB	NB	NB
**51**	GlcNAcb1-2Mana1-6(GlcNAcb1-2Mana1-3)Manb1-4GlcNAcb1-4GlcNAcb-Sp12	NB	NB	NB
**52**	GlcNAcb1-2Mana1-6(GlcNAcb1-2Mana1-3)Manb1-4GlcNAcb1-4GlcNAcb-Sp13	unsat.	NB	NB
**53**	Galb1-4GlcNAcb1-2Mana1-6(Galb1-4GlcNAcb1-2Mana1-3)Manb1-4GlcNAcb1-4GlcNAcb-Sp12	**0.62**	unsat.	NB
**54**	Neu5Aca2-6Galb1-4GlcNAcb1-2Mana1-6(Neu5Aca2-6Galb1-4GlcNAcb1-2Mana1-3)Manb1-4GlcNAcb1-4GlcNAcb-Sp12	unsat.	NB	NB
**55**	Neu5Aca2-6Galb1-4GlcNAcb1-2Mana1-6(Neu5Aca2-6Galb1-4GlcNAcb1-2Man-a1-3)Manb1-4GlcNAcb1-4GlcNAcb-Sp21	unsat.	NB	NB
**56**	Neu5Aca2-6Galb1-4GlcNAcb1-2Mana1-6(Neu5Aca2-6Galb1-4GlcNAcb1-2Mana1-3)Manb1-4GlcNAcb1-4GlcNAcb-Sp24	unsat.	NB	NB
**57**	Fuca1-2Galb1-3GalNAcb1-3Gala-Sp9	unsat.	NB	NB
**58**	Fuca1-2Galb1-3GalNAcb1-3Gala1-4Galb1-4Glcb-Sp9	NB	NB	NB
**59**	Fuca1-2Galb1-3(Fuca1-4)GlcNAcb-Sp8	NB	NB	NB
**60**	Fuca1-2Galb1-3GalNAca-Sp14	unsat.	NB	NB
**61**	Fuca1-2Galb1-3GalNAca-Sp8	unsat.	NB	NB
**62**	Fuca1-2Galb1-3GalNAcb1-4(Neu5Aca2-3)Galb1-4Glcb-Sp0	NB	NB	NB
**63**	Fuca1-2Galb1-3GalNAcb1-4(Neu5Aca2-3)Galb1-4Glcb-Sp9	NB	NB	NB
**64**	Fuca1-2Galb1-3GlcNAcb1-3Galb1-4Glcb-Sp10	**0.28**	unsat.	**2.56**
**65**	Fuca1-2Galb1-3GlcNAcb1-3Galb1-4Glcb-Sp8	**0.28**	unsat.	**3.11**
**66**	Fuca1-2Galb1-3GlcNAcb-Sp0	**1.03**	unsat.	NB
**67**	Fuca1-2Galb1-3GlcNAcb-Sp8	**0.91**	unsat.	NB
**68**	Fuca1-2Galb1-4(Fuca1-3)GlcNAcb1-3Galb1-4(Fuca1-3)GlcNAcb-Sp0	NB	NB	NB
**69**	Fuca1-2Galb1-4(Fuca1-3)GlcNAcb1-3Galb1-4(Fuca1-3)GlcNAcb1-3Galb1-4(Fuca1-3)GlcNAcb-Sp0	NB	NB	NB
**70**	Fuca1-2Galb1-4(Fuca1-3)GlcNAcb-Sp0	unsat.	NB	NB
**71**	Fuca1-2Galb1-4(Fuca1-3)GlcNAcb-Sp8	NB	NB	NB
**72**	Fuca1-2Galb1-4GlcNAcb1-3Galb1-4GlcNAcb-Sp0	**0.58**	unsat.	NB
**73**	Fuca1-2Galb1-4GlcNAcb1-3Galb1-4GlcNAcb1-3Galb1-4GlcNAcb-Sp0	NB	NB	NB
**74**	Fuca1-2Galb1-4GlcNAcb-Sp0	**1.83**	NB	NB

75	Fuca1-2Galb1-4GlcNAcb-Sp8	1.7	unsat.	NB
76	Fuca1-2Galb1-4Glcb-Sp0	2.25	NB	NB
77	Fuca1-2Galb-Sp8	NB	NB	NB
78	Fuca1-3GlcNAcb-Sp8	NB	NB	NB
79	Fuca1-4GlcNAcb-Sp8	NB	NB	NB
80	Fucb1-3GlcNAcb-Sp8	NB	NB	NB
81	GalNAca1-3(Fuca1-2)Galb1-3GlcNAcb-Sp0	0.28	unsat.	unsat.
82	GalNAca1-3(Fuca1-2)Galb1-4(Fuca1-3)GlcNAcb-Sp0	NB	NB	NB
83	(3S)Galb1-4(Fuca1-3)Glcb-Sp0	unsat.	NB	NB
84	GalNAca1-3(Fuca1-2)Galb1-4GlcNAcb-Sp0	0.28	unsat.	unsat.
85	GalNAca1-3(Fuca1-2)Galb1-4GlcNAcb-Sp8	0.31	unsat.	unsat.
86	GalNAca1-3(Fuca1-2)Galb1-4Glcb-Sp0	0.29	unsat.	3.08
87	GlcNAcb1-3Galb1-3GalNAca-Sp8	2.15	NB	unsat.
88	GalNAca1-3(Fuca1-2)Galb-Sp18	NB	NB	NB
89	GalNAca1-3(Fuca1-2)Galb-Sp8	NB	NB	NB
90	GalNAca1-3GalNAcb-Sp8	NB	NB	NB
91	GalNAca1-3Galb-Sp8	NB	NB	NB
92	GalNAca1-4(Fuca1-2)Galb1-4GlcNAcb-Sp8	unsat.	NB	NB
93	GalNAcb1-3GalNAca-Sp8	NB	NB	NB
94	GalNAcb1-3(Fuca1-2)Galb-Sp8	unsat.	NB	NB
95	GalNAcb1-3Gala1-4Galb1-4GlcNAcb-Sp0	unsat.	NB	NB
96	GalNAcb1-4(Fuca1-3)GlcNAcb-Sp0	NB	NB	NB
97	GalNAcb1-4GlcNAcb-Sp0	unsat.	NB	NB
98	GalNAcb1-4GlcNAcb-Sp8	unsat.	NB	NB
99	Gala1-2Galb-Sp8	NB	NB	NB
100	Gala1-3(Fuca1-2)Galb1-3GlcNAcb-Sp0	0.35	9.33	unsat.
101	Gala1-3(Fuca1-2)Galb1-3GlcNAcb-Sp8	0.31	unsat.	unsat.
102	Gala1-3(Fuca1-2)Galb1-4(Fuca1-3)GlcNAcb-Sp0	NB	NB	NB
103	Gala1-3(Fuca1-2)Galb1-4(Fuca1-3)GlcNAcb-Sp8	NB	NB	NB
104	Gala1-3(Fuca1-2)Galb1-4GlcNAc-Sp0	0.29	6.55	unsat.
105	Gala1-3(Fuca1-2)Galb1-4Glcb-Sp0	0.3	unsat.	unsat.
106	Gala1-3(Fuca1-2)Galb-Sp18	NB	NB	NB
107	Gala1-3(Fuca1-2)Galb-Sp8	unsat.	NB	NB
108	Gala1-4(Gala1-3)Galb1-4GlcNAcb-Sp8	NB	NB	NB
109	Gala1-3GalNAca-Sp16	NB	NB	NB
110	Gala1-3GalNAca-Sp8	unsat.	NB	NB
111	Gala1-3GalNAcb-Sp8	NB	NB	NB
112	Gala1-3Galb1-4(Fuca1-3)GlcNAcb-Sp8	NB	NB	NB
113	Gala1-3Galb1-3GlcNAcb-Sp0	0.38	unsat.	NB
114	Gala1-3Galb1-4GlcNAcb-Sp8	0.68	unsat.	NB
115	Gala1-3Galb1-4Glcb-Sp0	0.64	NB	unsat.
116	Gala1-3Galb1-4Glc-Sp10	0.59	NB	unsat.

**117**	Gala1-3Galb-Sp8	NB	NB	NB
**118**	Gala1-4(Fuca1-2)Galb1-4GlcNAcb-Sp8	NB	NB	NB
**119**	Gala1-4Galb1-4GlcNAcb-Sp0	NB	NB	NB
**120**	Gala1-4Galb1-4GlcNAcb-Sp8	NB	NB	NB
**121**	Gala1-4Galb1-4Glcb-Sp0	NB	NB	NB
**122**	GalNAca-Sp15	NB	NB	NB
**123**	Gala1-4GlcNAcb-Sp8	NB	NB	NB
**124**	Gala1-6Glcb-Sp8	NB	NB	NB
**125**	Galb1-2Galb-Sp8	NB	NB	NB
**126**	Galb1-3(Fuca1-4)GlcNAcb1-3Galb1-4(Fuca1-3)GlcNAcb-Sp0	NB	NB	NB
**127**	Galb1-3GlcNAcb1-3Galb1-4(Fuca1-3)GlcNAcb-Sp0	**1.17**	unsat.	NB
**128**	Galb1-3(Fuca1-4)GlcNAc-Sp0	NB	NB	NB
**129**	Galb1-3(Fuca1-4)GlcNAc-Sp8	NB	NB	NB
**130**	Fuca1-4(Galb1-3)GlcNAcb-Sp8	NB	NB	NB
**131**	Galb1-4GlcNAcb1-6GalNAc-Sp14	unsat.	NB	NB
**132**	Galb1-4GlcNAcb1-6GalNAca-Sp8	unsat.	NB	NB
**133**	GlcNAcb1-6(Galb1-3)GalNAca-Sp8	**3.1**	NB	NB
**134**	Neu5Aca2-6(Galb1-3)GalNAca-Sp14	NB	NB	NB
**135**	Neu5Aca2-6(Galb1-3)GalNAca-Sp8	unsat.	NB	NB
**136**	Neu5Acb2-6(Galb1-3)GalNAca-Sp8	NB	NB	NB
**137**	Neu5Aca2-6(Galb1-3)GlcNAcb1-4Galb1-4Glcb-Sp10	**2.63**	NB	NB
**138**	Galb1-3GalNAca-Sp14	NB	NB	NB
**139**	Galb1-3GalNAca-Sp16	NB	NB	NB
**140**	Galb1-3GalNAca-Sp8	NB	NB	NB
**141**	Galb1-3GalNAcb-Sp8	**2.79**	NB	unsat.
**142**	Galb1-3GalNAcb1-3Gala1-4Galb1-4Glcb-Sp0	**0.74**	NB	NB
**143**	Galb1-3GalNAcb1-4(Neu5Aca2-3)Galb1-4Glcb-Sp0	**3.14**	NB	NB
**144**	Galb1-3GalNAcb1-4Galb1-4Glcb-Sp8	**1.86**	NB	NB
**145**	Galb1-3Galb-Sp8	**2.09**	NB	NB
**146**	Galb1-3GlcNAcb1-3Galb1-4GlcNAcb-Sp0	**0.41**	unsat.	unsat.
**147**	Galb1-3GlcNAcb1-3Galb1-4Glcb-Sp10	**0.35**	unsat.	**9.64**
**148**	Galb1-3GlcNAcb-Sp0	**1.37**	NB	NB
**149**	Galb1-3GlcNAcb-Sp8	**1.65**	NB	NB
**150**	Galb1-4(Fuca1-3)GlcNAcb-Sp0	NB	NB	NB
**151**	Galb1-4(Fuca1-3)GlcNAcb-Sp8	NB	NB	NB
**152**	Galb1-4(Fuca1-3)GlcNAcb1-3Galb1-4(Fuca1-3)GlcNAcb-Sp0	NB	NB	NB
**153**	Galb1-4(Fuca1-3)GlcNAcb1-3Galb1-4(Fuca1-3)GlcNAcb1-3Galb1-4(Fuca1-3)GlcNAcb-Sp0	unsat.	NB	NB
**154**	Galb1-4(6S)Glcb-Sp0	**2.22**	NB	NB
**155**	Galb1-4(6S)Glcb-Sp8	**2.39**	NB	NB
**156**	Galb1-4GalNAca1-3(Fuca1-2)Galb1-4GlcNAcb-Sp8	**0.3**	unsat.	**5.55**
**157**	Galb1-4GalNAcb1-3(Fuca1-2)Galb1-4GlcNAcb-Sp8	**2.92**	NB	NB

**158**	Galb1-4GlcNAcb1-3GalNAc-Sp14	**2.78**	NB	NB
**159**	Galb1-4GlcNAcb1-3GalNAca-Sp8	**1.48**	NB	NB
**160**	Galb1-4GlcNAcb1-3Galb1-4(Fuca1-3)GlcNAcb1-3Galb1-4(Fuca1-3)GlcNAcb-Sp0	**1.06**	unsat.	NB
**161**	Galb1-4GlcNAcb1-3Galb1-4GlcNAcb1-3Galb1-4GlcNAcb-Sp0	**0.25**	**6.25**	unsat.
**162**	Galb1-4GlcNAcb1-3Galb1-4GlcNAcb-Sp0	**0.33**	unsat.	unsat.
**163**	Galb1-4GlcNAcb1-3Galb1-4Glcb-Sp0	**0.33**	unsat.	**2.52**
**164**	Galb1-4GlcNAcb1-3Galb1-4Glcb-Sp8	**0.32**	unsat.	**2.24**
**165**	Galb1-4GlcNAcb1-6(Galb1-3)GalNAc-Sp14	**1.99**	NB	NB
**166**	Galb1-4GlcNAcb1-6(Galb1-3)GalNAca-Sp8	**1.41**	NB	NB
**167**	Galb1-4GlcNAcb-Sp0	unsat.	NB	NB
**168**	Galb1-4GlcNAcb-Sp8	**1.98**	NB	NB
**169**	Galb1-4GlcNAcb-Sp23	unsat.	NB	NB
**170**	Galb1-4Glcb-Sp0	unsat.	NB	NB
**171**	Galb1-4Glcb-Sp8	unsat.	NB	NB
**172**	GlcNAca1-3Galb1-4GlcNAcb-Sp8	**1.26**	NB	unsat.
**173**	GlcNAca1-6Galb1-4GlcNAcb-Sp8	NB	NB	NB
**174**	GlcNAcb1-2Galb1-3GalNAca-Sp8	unsat.	NB	NB
**175**	GlcNAcb1-6(GlcNAcb1-3)GalNAca-Sp14	NB	NB	NB
**176**	GlcNAcb1-6(GlcNAcb1-3)GalNAca-Sp8	NB	NB	NB
**177**	GlcNAcb1-6(GlcNAcb1-3)Galb1-4GlcNAcb-Sp8	unsat.	NB	NB
**178**	GlcNAcb1-3GalNAca-Sp14	NB	NB	NB
**179**	GlcNAcb1-3GalNAca-Sp8	unsat.	NB	NB
**180**	GlcNAcb1-3Galb-Sp8	NB	NB	NB
**181**	GlcNAcb1-3Galb1-4GlcNAcb-Sp0	**0.62**	unsat.	unsat.
**182**	GlcNAcb1-3Galb1-4GlcNAcb-Sp8	**0.56**	unsat.	unsat.
**183**	GlcNAcb1-3Galb1-4GlcNAcb1-3Galb1-4GlcNAcb-Sp0	**0.27**	**9.24**	unsat.
**184**	GlcNAcb1-3Galb1-4Glcb-Sp0	**0.67**	NB	unsat.
**185**	GlcNAcb1-4-MDPLys	NB	NB	NB
**186**	GlcNAcb1-6(GlcNAcb1-4)GalNAca-Sp8	NB	NB	NB
**187**	GlcNAcb1-4Galb1-4GlcNAcb-Sp8	NB	NB	NB
**188**	GlcNAcb1-4GlcNAcb1-4GlcNAcb1-4GlcNAcb1-4GlcNAcb1-4GlcNAcb1-Sp8	NB	NB	NB
**189**	GlcNAcb1-4GlcNAcb1-4GlcNAcb1-4GlcNAcb1-4GlcNAcb1-Sp8	NB	NB	NB
**190**	GlcNAcb1-4GlcNAcb1-4GlcNAcb-Sp8	NB	NB	NB
**191**	GlcNAcb1-6GalNAca-Sp14	NB	NB	NB
**192**	GlcNAcb1-6GalNAca-Sp8	unsat.	NB	NB
**193**	GlcNAcb1-6Galb1-4GlcNAcb-Sp8	unsat.	NB	NB
**194**	Glca1-4Glcb-Sp8	unsat.	NB	NB
**195**	Glca1-4Glca-Sp8	NB	NB	NB

**196**	Glca1-6Glca1-6Glcb-Sp8	NB	NB	NB
**197**	Glcb1-4Glcb-Sp8	NB	NB	NB
**198**	Glcb1-6Glcb-Sp8	NB	NB	NB
**199**	G-ol-Sp8	NB	NB	NB
**200**	GlcAa-Sp8	NB	NB	NB
**201**	GlcAb-Sp8	NB	NB	NB
**202**	GlcAb1-3Galb-Sp8	NB	NB	NB
**203**	GlcAb1-6Galb-Sp8	NB	NB	NB
**204**	KDNa2-3Galb1-3GlcNAcb-Sp0	unsat.	NB	NB
**205**	KDNa2-3Galb1-4GlcNAcb-Sp0	unsat.	NB	NB
**206**	Mana1-2Mana1-2Mana1-3Mana-Sp9	NB	NB	NB
**207**	Mana1-2Mana1-6(Mana1-2Mana1-3)Mana-Sp9	NB	NB	NB
**208**	Mana1-2Mana1-3Mana-Sp9	NB	NB	NB
**209**	Mana1-6(Mana1-3)Mana-Sp9	NB	NB	NB
**210**	Mana1-2Mana1-2Mana1-6(Mana1-3)Mana-Sp9	NB	NB	NB
**211**	Mana1-6(Mana1-3)Mana1-6(Mana1-2Mana1-3)Manb1-4GlcNAcb1-4GlcNAcb-Sp12	unsat.	unsat.	NB
**212**	Mana1-6(Mana1-3)Mana1-6(Mana1-3)Manb1-4GlcNAcb1-4GlcNAcb-Sp12	NB	NB	NB
**213**	Manb1-4GlcNAcb-Sp0	NB	NB	NB
**214**	Neu5Aca2-3Galb1-4GlcNAcb1-3Galb1-4(Fuca1-3)GlcNAcb-Sp0	NB	NB	NB
**215**	(3S)Galb1-4(Fuca1-3)(6S)GlcNAcb-Sp8	**1.51**	unsat.	NB
**216**	Fuca1-2(6S)Galb1-4GlcNAcb-Sp0	NB	NB	NB
**217**	Fuca1-2Galb1-4(6S)GlcNAcb-Sp8	**1.69**	unsat.	NB
**218**	Fuca1-2(6S)Galb1-4(6S)Glcb-Sp0	NB	NB	NB
**219**	Neu5Aca2-3Galb1-3GalNAca-Sp14	unsat.	NB	NB
**220**	Neu5Aca2-3Galb1-3GalNAca-Sp8	unsat.	NB	NB
**221**	GalNAcb1-4(Neu5Aca2-8Neu5Aca2-8Neu5Aca2-8Neu5Aca2-3)Galb1-4Glcb-Sp0	NB	NB	NB
**222**	GalNAcb1-4(Neu5Aca2-8Neu5Aca2-8Neu5Aca2-3)Galb1-4Glcb-Sp0	NB	NB	NB
**223**	Neu5Aca2-8Neu5Aca2-8Neu5Aca2-3Galb1-4Glcb-Sp0	unsat.	NB	NB
**224**	GalNAcb1-4(Neu5Aca2-8Neu5Aca2-3)Galb1-4Glcb-Sp0	NB	NB	NB
**225**	Neu5Aca2-8Neu5Aca2-8Neu5Aca-Sp8	NB	NB	NB
**226**	GalNAcb1-4(Neu5Aca2-3)Galb1-4GlcNAcb-Sp0	NB	NB	NB
**227**	GalNAcb1-4(Neu5Aca2-3)Galb1-4GlcNAcb-Sp8	**2.68**	**4.55**	**5.15**
**228**	GalNAcb1-4(Neu5Aca2-3)Galb1-4Glcb-Sp0	NB	NB	NB
**229**	Neu5Aca2-3Galb1-3GalNAcb1-4(Neu5Aca2-3)Galb1-4Glcb-Sp0	unsat.	NB	NB
**230**	Neu5Aca2-6(Neu5Aca2-3)GalNAca-Sp8	NB	NB	NB
**231**	Neu5Aca2-3GalNAca-Sp8	unsat.	NB	NB

**232**	Neu5Aca2-3GalNAcb1-4GlcNAcb-Sp0	unsat.	NB	NB
**233**	Neu5Aca2-3Galb1-3(6S)GlcNAc-Sp8	unsat.	NB	NB
**234**	Neu5Aca2-3Galb1-3(Fuca1-4)GlcNAcb-Sp8	NB	NB	NB
**235**	Neu5Aca2-3Galb1-3(Fuca1-4)GlcNAcb1-3Galb1-4(Fuca1-3)GlcNAcb-Sp0	NB	NB	NB
**236**	Neu5Aca2-3Galb1-4(Neu5Aca2-3Galb1-3)GlcNAcb-Sp8	NB	NB	NB
**237**	Neu5Aca2-3Galb1-3(6S)GalNAca-Sp8	**2.31**	NB	NB
**238**	Neu5Aca2-6(Neu5Aca2-3Galb1-3)GalNAca-Sp14	NB	NB	unsat.
**239**	Neu5Aca2-6(Neu5Aca2-3Galb1-3)GalNAca-Sp8	NB	NB	NB
**240**	Neu5Aca2-3Galb-Sp8	NB	NB	NB
**241**	Neu5Aca2-3Galb1-3GalNAcb1-3Gala1-4Galb1-4Glcb-Sp0	**0.71**	unsat.	unsat.
**242**	Neu5Aca2-3Galb1-3GlcNAcb1-3Galb1-4GlcNAcb-Sp0	**0.33**	unsat.	unsat.
**243**	Fuca1-2(6S)Galb1-4Glcb-Sp0	NB	NB	NB
**244**	Neu5Aca2-3Galb1-3GlcNAcb-Sp0	unsat.	NB	NB
**245**	Neu5Aca2-3Galb1-4(6S)GlcNAcb-Sp8	unsat.	NB	NB
**246**	Neu5Aca2-3Galb1-4(Fuca1-3)(6S)GlcNAcb-Sp8	NB	NB	NB
**247**	Neu5Aca2-3Galb1-4(Fuca1-3)GlcNAcb1-3Galb1-4(Fuca1-3)GlcNAcb1-3Galb1-4(Fuca1-3)GlcNAcb-Sp0	NB	NB	NB
**248**	Neu5Aca2-3Galb1-4(Fuca1-3)GlcNAcb-Sp0	NB	NB	NB
**249**	Neu5Aca2-3Galb1-4(Fuca1-3)GlcNAcb-Sp8	NB	NB	NB
**250**	Neu5Aca2-3Galb1-4(Fuca1-3)GlcNAcb1-3Galb-Sp8	NB	NB	NB
**251**	Neu5Aca2-3Galb1-4(Fuca1-3)GlcNAcb1-3Galb1-4GlcNAcb-Sp8	unsat.	NB	NB
**252**	Neu5Aca2-3Galb1-4GlcNAcb1-3Galb1-4GlcNAcb1-3Galb1-4GlcNAcb-Sp0	**0.42**	unsat.	unsat.
**253**	Neu5Aca2-3Galb1-4GlcNAcb-Sp0	unsat.	NB	NB
**254**	Neu5Aca2-3Galb1-4GlcNAcb-Sp8	unsat.	NB	NB
**255**	Neu5Aca2-3Galb1-4GlcNAcb1-3Galb1-4GlcNAcb-Sp0	unsat.	NB	NB
**256**	Fuca1-2Galb1-4(6S)Glcb-Sp0	**2.31**	NB	NB
**257**	Neu5Aca2-3Galb1-4Glcb-Sp0	unsat.	NB	NB
**258**	Neu5Aca2-3Galb1-4Glcb-Sp8	unsat.	NB	NB
**259**	Neu5Aca2-6GalNAca-Sp8	NB	NB	NB
**260**	Neu5Aca2-6GalNAcb1-4GlcNAcb-Sp0	NB	NB	NB
**261**	Neu5Aca2-6Galb1-4(6S)GlcNAcb-Sp8	NB	NB	NB
**262**	Neu5Aca2-6Galb1-4GlcNAcb-Sp8	NB	NB	NB
**263**	Neu5Aca2-6Galb1-4GlcNAcb1-3Galb1-4(Fuca1-3)GlcNAcb1-3Galb1-4(Fuca1-3)GlcNAcb-Sp0	unsat.	NB	NB
**264**	Neu5Aca2-6Galb1-4GlcNAcb1-3Galb1-4GlcNAcb-Sp0	**0.36**	unsat.	**6.21**
**265**	Neu5Aca2-6Galb1-4Glcb-Sp0	unsat.	NB	NB
**266**	Neu5Aca2-6Galb1-4Glcb-Sp8	NB	NB	NB
**267**	Neu5Aca2-6Galb-Sp8	NB	NB	NB
**268**	Neu5Aca2-8Neu5Aca-Sp8	NB	NB	NB

**269**	Neu5Aca2-8Neu5Aca2-3Galb1-4Glcb-Sp0	unsat.	NB	NB
**270**	Galb1-3(Fuca1-4)GlcNAcb1-3Galb1-3(Fuca1-4)GlcNAcb-Sp0	NB	NB	NB
**271**	Neu5Acb2-6GalNAca-Sp8	NB	NB	NB
**272**	Neu5Acb2-6Galb1-4GlcNAcb-Sp8	NB	NB	NB
**273**	Neu5Gca2-3Galb1-3(Fuca1-4)GlcNAcb-Sp0	NB	NB	NB
**274**	Neu5Gca2-3Galb1-3GlcNAcb-Sp0	unsat.	NB	NB
**275**	Neu5Gca2-3Galb1-4(Fuca1-3)GlcNAcb-Sp0	NB	NB	NB
**276**	Neu5Gca2-3Galb1-4GlcNAcb-Sp0	unsat.	NB	NB
**277**	Neu5Gca2-3Galb1-4Glcb-Sp0	unsat.	NB	NB
**278**	Neu5Gca2-6GalNAca-Sp0	NB	NB	NB
**279**	Neu5Gca2-6Galb1-4GlcNAcb-Sp0	NB	NB	NB
**280**	Neu5Gca-Sp8	NB	NB	NB
**281**	Neu5Aca2-3Galb1-4GlcNAcb1-6(Galb1-3)GalNAca-Sp14	unsat.	NB	NB
**282**	Galb1-3GlcNAcb1-3Galb1-3GlcNAcb-Sp0	**0.24**	unsat.	**7.06**
**283**	Galb1-4(Fuca1-3)(6S)GlcNAcb-Sp0	unsat.	NB	NB
**284**	Galb1-4(Fuca1-3)(6S)Glcb-Sp0	NB	NB	NB
**285**	Galb1-4(Fuca1-3)GlcNAcb1-3Galb1-3(Fuca1-4)GlcNAcb-Sp0	NB	NB	NB
**286**	Galb1-4GlcNAcb1-3Galb1-3GlcNAcb-Sp0	**0.27**	unsat.	**7.24**
**287**	Neu5Aca2-3Galb1-3GlcNAcb1-3Galb1-3GlcNAcb-Sp0	**0.28**	unsat.	unsat.
**288**	Neu5Aca2-3Galb1-4GlcNAcb1-3Galb1-3GlcNAcb-Sp0	**1.61**	NB	NB
**289**	4S(3S)Galb1-4GlcNAcb-Sp0	NB	NB	NB
**290**	(6S)Galb1-4(6S)GlcNAcb-Sp0	unsat.	NB	NB
**291**	(6P)Glcb-Sp10	NB	NB	NB
**292**	Galb1-3Galb1-4GlcNAcb-Sp8	**0.27**	unsat.	NB
**293**	Neu5Aca2-6Galb1-4GlcNAcb1-2Mana1-6(Galb1-4GlcNAcb1-2Mana1-3)Manb1-4GlcNAcb1-4GlcNAcb-Sp12	**2.72**	NB	NB
**294**	Galb1-4GlcNAcb1-6(Galb1-4GlcNAcb1-3)Galb1-4GlcNAc-Sp0	**0.93**	unsat.	NB
**295**	GlcNAcb1-6(Galb1-4GlcNAcb1-3)Galb1-4GlcNAc-Sp0	**1.26**	unsat.	NB
**296**	Galb1-4GlcNAca1-6Galb1-4GlcNAcb-Sp0	**1.59**	NB	NB
**297**	Galb1-4GlcNAcb1-6Galb1-4GlcNAcb-Sp0	**2.38**	NB	NB
**298**	GalNAcb1-3Galb-Sp8	**1.54**	NB	NB
**299**	GlcAb1-3GlcNAcb-Sp8	NB	NB	NB
**300**	Neu5Aca2-6Galb1-4GlcNAcb1-2Mana1-6(GlcNAcb1-2Mana1-3)Manb1-4GlcNAcb1-4GlcNAcb-Sp12	NB	NB	NB
**301**	GlcNAcb1-3Man-Sp10	NB	NB	NB
**302**	GlcNAcb1-4GlcNAcb-Sp10	NB	NB	NB
**303**	GlcNAcb1-4GlcNAcb-Sp12	NB	NB	NB
**304**	MurNAcb1-4GlcNAcb-Sp10	NB	NB	NB
**305**	Mana1-6Manb-Sp10	NB	NB	NB
**306**	Mana1-6(Mana1-3)Mana1-6(Mana1-3)Manb-Sp10	NB	NB	NB

**307**	Mana1-2Mana1-6(Mana1-3)Mana1-6(Mana1-2Mana1-2Mana1-3)Mana-Sp9	NB	NB	NB
**308**	Mana1-2Mana1-6(Mana1-2Mana1-3)Mana1-6(Mana1-2Mana1-2Mana1-3)Mana-Sp9	unsat.	NB	NB
**309**	Neu5Aca2-3Galb1-4GlcNAcb1-6(Neu5Aca2-3Galb1-3)GalNAca-Sp14	unsat.	NB	NB
**310**	Neu5Aca2-6Galb1-4GlcNAcb1-2Mana1-6(Neu5Aca2-3Galb1-4GlcNAcb1-2Mana1-3)Manb1-4GlcNAcb1-4GlcNAcb-Sp12	unsat.	NB	NB
**311**	Galb1-4GlcNAcb1-2Mana1-6(Neu5Aca2-6Galb1-4GlcNAcb1-2Mana1-3)Manb1-4GlcNAcb1-4GlcNAcb-Sp12	unsat.	NB	NB
**312**	Neu5Aca2-8Neu5Aca2-8Neu5Acb-Sp8	NB	NB	NB
**313**	Neu5Gcb2-6Galb1-4GlcNAc-Sp8	NB	NB	NB
**314**	Galb1-3GlcNAcb1-2Mana1-6(Galb1-3GlcNAcb1-2Mana1-3)Manb1-4GlcNAcb1-4GlcNAcb-Sp19	**0.42**	unsat.	unsat.
**315**	Neu5Aca2-3Galb1-4GlcNAcb1-2Mana1-6(Neu5Aca2-3Galb1-4GlcNAcb1-2Mana1-3)Manb1-4GlcNAcb1-4GlcNAcb-Sp12	unsat.	NB	NB
**316**	Neu5Aca2-3Galb1-4GlcNAcb1-2Mana1-6(Neu5Aca2-6Galb1-4GlcNAcb1-2Mana1-3)Manb1-4GlcNAcb1-4GlcNAcb-Sp12	**2.4**	NB	NB
**317**	Galb1-4(Fuca1-3)GlcNAcb1-2Mana1-6(Galb1-4(Fuca1-3)GlcNAcb1-2Mana1-3)Manb1-4GlcNAcb1-4GlcNAcb-Sp20	NB	NB	NB
**318**	Neu5,9Ac2a2-3Galb1-3GlcNAcb-Sp0	unsat.	NB	NB
**319**	Neu5Aca2-6Galb1-4GlcNAcb1-3Galb1-3GlcNAcb-Sp0	**0.28**	**8.1**	**0.51**
**320**	Neu5Aca2-3Galb1-3(Fuca1-4)GlcNAcb1-3Galb1-3(Fuca1-4)GlcNAcb-Sp0	NB	NB	NB
**321**	Neu5Aca2-6Galb1-4GlcNAcb1-3Galb1-4GlcNAcb1-3Galb1-4GlcNAcb-Sp0	**0.26**	**7.68**	**2.51**
**322**	Gala1-4Galb1-4GlcNAcb1-3Galb1-4Glcb-Sp0	**0.3**	unsat.	**3.61**
**323**	GalNAcb1-3Gala1-4Galb1-4GlcNAcb1-3Galb1-4Glcb-Sp0	**0.27**	unsat.	**5.92**
**324**	GalNAca1-3(Fuca1-2)Galb1-4GlcNAcb1-3Galb1-4GlcNAcb-Sp0	**0.23**	**8.12**	**4.48**
**325**	GalNAca1-3(Fuca1-2)Galb1-4GlcNAcb1-3Galb1-4GlcNAcb1-3Galb1-4GlcNAcb-Sp0	**0.21**	**9.94**	unsat.
**326**	Neu5Aca2-3Galb1-4(Fuca1-3)GlcNAcb1-6(Neu5Aca2-3Galb1-3)GalNAc-Sp14	unsat.	NB	NB
**327**	GlcNAca1-4Galb1-4GlcNAcb1-3Galb1-4GlcNAcb1-3Galb1-4GlcNAcb-Sp0	**0.28**	**8.03**	unsat.
**328**	GlcNAca1-4Galb1-4GlcNAcb-Sp0	NB	NB	NB
**329**	GlcNAca1-4Galb1-3GlcNAcb-Sp0	NB	NB	NB
**330**	GlcNAca1-4Galb1-4GlcNAcb1-3Galb1-4Glcb-Sp0	**0.4**	unsat.	unsat.
**331**	GlcNAca1-4Galb1-4GlcNAcb1-3Galb1-4(Fuca1-3)GlcNAcb1-3Galb1-4(Fuca1-3)GlcNAcb-Sp0	unsat.	NB	NB
**332**	GlcNAca1-4Galb1-4GlcNAcb1-3Galb1-4GlcNAcb-Sp0	**0.3**	unsat.	unsat.

**333**	GlcNAca1-4Galb1-3GalNAc-Sp14	NB	NB	NB
**334**	Neu5Aca2-6Galb1-4GlcNAcb1-2Mana1-6(Mana1-3)Manb1-4GlcNAcb1-4GlcNAc-Sp12	NB	NB	NB
**335**	Mana1-6(Neu5Aca2-6Galb1-4GlcNAcb1-2Mana1-3)Manb1-4GlcNAcb1-4GlcNAc-Sp12	unsat.	NB	NB
**336**	Neu5Aca2-6Galb1-4GlcNAcb1-2Mana1-6Manb1-4GlcNAcb1-4GlcNAc-Sp12	NB	NB	NB
**337**	Neu5Aca2-6Galb1-4GlcNAcb1-2Mana1-3Manb1-4GlcNAcb1-4GlcNAc-Sp12	NB	NB	NB
**338**	Galb1-4GlcNAcb1-2Mana1-3Manb1-4GlcNAcb1-4GlcNAc-Sp12	**1.36**	NB	NB
**339**	Galb1-4GlcNAcb1-2Mana1-6Manb1-4GlcNAcb1-4GlcNAc-Sp12	**2.58**	NB	NB
**340**	Mana1-6(Galb1-4GlcNAcb1-2Mana1-3)Manb1-4GlcNAcb1-4GlcNAcb-Sp12	**1.55**	NB	NB
**341**	GlcNAcb1-2Mana1-6(GlcNAcb1-2Mana1-3)Manb1-4GlcNAcb1-4(Fuca1-6)GlcNAcb-Sp22	NB	NB	NB
**342**	Galb1-4GlcNAcb1-2Mana1-6(Galb1-4GlcNAcb1-2Mana1-3)Manb1-4GlcNAcb1-4(Fuca1-6)GlcNAcb-Sp22	**1.09**	unsat.	NB
**343**	Galb1-3GlcNAcb1-2Mana1-6(Galb1-3GlcNAcb1-2Mana1-3)Manb1-4GlcNAcb1-4(Fuca1-6)GlcNAcb-Sp22	**0.95**	unsat.	NB
**344**	(6S)GlcNAcb1-3Galb1-4GlcNAcb-Sp0	unsat.	NB	NB
**345**	KDNa2-3Galb1-4(Fuca1-3)GlcNAc-Sp0	NB	NB	NB
**346**	KDNa2-6Galb1-4GlcNAc-Sp0	unsat.	NB	NB
**347**	KDNa2-3Galb1-4Glc-Sp0	NB	NB	NB
**348**	KDNa2-3Galb1-3GalNAca-Sp14	unsat.	NB	NB
**349**	Fuca1-2Galb1-3GlcNAcb1-2Mana1-6(Fuca1-2Galb1-3GlcNAcb1-2Mana1-3)Manb1-4GlcNAcb1-4GlcNAcb-Sp20	**1.39**	unsat.	unsat.
**350**	Fuca1-2Galb1-4GlcNAcb1-2Mana1-6(Fuca1-2Galb1-4GlcNAcb1-2Mana1-3)Manb1-4GlcNAcb1-4GlcNAcb-Sp20	**0.91**	unsat.	unsat.
**351**	Fuca1-2Galb1-4(Fuca1-3)GlcNAcb1-2Mana1-6(Fuca1-2Galb1-4(Fuca1-3)GlcNAcb1-2Mana1-3)Manb1-4GlcNAcb1-4GlcNAb-Sp20	unsat.	NB	NB
**352**	Gala1-3Galb1-4GlcNAcb1-2Mana1-6(Gala1-3Galb1-4GlcNAcb1-2Mana1-3)Manb1-4GlcNAcb1-4GlcNAcb-Sp20	**0.24**	**8.99**	unsat.
**353**	Galb1-4GlcNAcb1-2Mana1-6(Mana1-3)Manb1-4GlcNAcb1-4GlcNAcb-Sp12	**2.65**	NB	NB
**354**	Fuca1-4(Galb1-3)GlcNAcb1-2Mana1-6(Fuca1-4(Galb1-3)GlcNAcb1-2Mana1-3)Manb1-4GlcNAcb1-4(Fuca1-6)GlcNAcb-Sp22	NB	unsat.	unsat.
**355**	Neu5Aca2-6GlcNAcb1-4GlcNAc-Sp21	NB	NB	NB
**356**	Neu5Aca2-6GlcNAcb1-4GlcNAcb1-4GlcNAc-Sp21	NB	NB	NB

**357**	Galb1-4(Fuca1-3)GlcNAcb1-6(Fuca1-2Galb1-4GlcNAcb1-3)Galb1-4Glc-Sp21	**0.7**	unsat.	NB
**358**	Galb1-4GlcNAcb1-2Mana1-6(Galb1-4GlcNAcb1-4(Galb1-4GlcNAcb1-2)Mana1-3)Manb1-4GlcNAcb1-4GlcNAc-Sp21	**0.3**	unsat.	NB
**359**	GalNAca1-3(Fuca1-2)Galb1-4GlcNAcb1-2Mana1-6(GalNAca1-3(Fuca1-2)Galb1-4GlcNAcb1-2Mana1-3)Manb1-4GlcNAcb1-4GlcNAcb-Sp20	**0.2**	**4.55**	**1.92**
**360**	Gala1-3(Fuca1-2)Galb1-4GlcNAcb1-2Mana1-6(Gala1-3(Fuca1-2)Galb1-4GlcNAcb1-2Mana1-3)Manb1-4GlcNAcb1-4GlcNAcb-Sp20	**0.21**	**2.3**	unsat.
**361**	Gala1-3Galb1-4(Fuca1-3)GlcNAcb1-2Mana1-6(Gala1-3Galb1-4(Fuca1-3)GlcNAcb1-2Mana1-3)Manb1-4GlcNAcb1-4GlcNAcb-Sp20	**2.92**	NB	NB
**362**	GalNAca1-3(Fuca1-2)Galb1-3GlcNAcb1-2Mana1-6(GalNAca1-3(Fuca1-2)Galb1-3GlcNAcb1-2Mana1-3)Manb1-4GlcNAcb1-4GlcNAcb-Sp20	**0.27**	unsat.	**0.29**
**363**	Gala1-3(Fuca1-2)Galb1-3GlcNAcb1-2Mana1-6(Gala1-3(Fuca1-2)Galb1-3GlcNAcb1-2Mana1-3)Manb1-4GlcNAcb1-4GlcNAcb-Sp20	**0.27**	**3.22**	**1.68**
**364**	Fuca1-4(Fuca1-2Galb1-3)GlcNAcb1-2Mana1-3(Fuca1-4(Fuca1-2Galb1-3)GlcNAcb1-2Mana1-3)Manb1-4GlcNAcb1-4GlcNAcb-Sp19	NB	NB	NB
**365**	Neu5Aca2-3Galb1-4GlcNAcb1-3GalNAc-Sp14	unsat.	NB	NB
**366**	Neu5Aca2-6Galb1-4GlcNAcb1-3GalNAc-Sp14	NB	NB	NB
**367**	Neu5Aca2-3Galb1-4(Fuca1-3)GlcNAcb1-3GalNAca-Sp14	unsat.	NB	NB
**368**	GalNAcb1-4GlcNAcb1-2Mana1-6(GalNAcb1-4GlcNAcb1-2Mana1-3)Manb1-4GlcNAcb1-4GlcNAc-Sp12	**2.4**	unsat.	unsat.
**369**	Galb1-3GalNAca1-3(Fuca1-2)Galb1-4Glc-Sp0	**0.26**	unsat.	**3.73**
**370**	Galb1-3GalNAca1-3(Fuca1-2)Galb1-4GlcNAc-Sp0	**0.25**	unsat.	unsat.
**371**	Galb1-3GlcNAcb1-3Galb1-4GlcNAcb1-6(Galb1-3GlcNAcb1-3)Galb1-4Glcb-Sp21	**0.27**	unsat.	unsat.
**372**	Galb1-4(Fuca1-3)GlcNAcb1-6(Galb1-3GlcNAcb1-3)Galb1-4Glc-Sp21	**1.69**	NB	NB
**373**	Galb1-4GlcNAcb1-6(Fuca1-4(Fuca1-2Galb1-3)GlcNAcb1-3)Galb1-4Glc-Sp21	**2.01**	NB	NB
**374**	Galb1-4(Fuca1-3)GlcNAcb1-6(Fuca1-4(Fuca1-2Galb1-3)GlcNAcb1-3)Galb1-4Glc-Sp21	**2.1**	NB	NB
**375**	Galb1-3GlcNAcb1-3Galb1-4(Fuca1-3)GlcNAcb1-6(Galb1-3GlcNAcb1-3)Galb1-4Glc-Sp21	**0.38**	unsat.	NB
**376**	Galb1-4GlcNAcb1-6(Galb1-4GlcNAcb1-2)Mana1-6(Galb1-4GlcNAcb1-4(Galb1-4GlcNAcb1-2)Mana1-3)Manb1-4GlcNAcb1-4GlcNAcb-Sp21	**0.29**	unsat.	NB

**377**	GlcNAcb1-2Mana1-6(GlcNAcb1-4(GlcNAcb1-2)Mana1-3)Manb1-4GlcNAcb1-4GlcNAc-Sp21	unsat.	NB	NB
**378**	Fuca1-2Galb1-3GalNAca1-3(Fuca1-2)Galb1-4Glcb-Sp0	**0.88**	NB	NB
**379**	Fuca1-2Galb1-3GalNAca1-3(Fuca1-2)Galb1-4GlcNAcb-Sp0	**0.43**	unsat.	NB
**380**	Galb1-3GlcNAcb1-3GalNAca-Sp14	**2.96**	NB	NB
**381**	GalNAcb1-4(Neu5Aca2-3)Galb1-4GlcNAcb1-3GalNAca-Sp14		NB	NB
**382**	GalNAca1-3(Fuca1-2)Galb1-3GalNAca1-3(Fuca1-2)Galb1-4GlcNAcb-Sp0	**0.33**	unsat.	NB
**383**	Gala1-3Galb1-3GlcNAcb1-2Mana1-6(Gala1-3Galb1-3GlcNAcb1-2Mana1-3)Manb1-4GlcNAcb1-4GlcNAc-Sp19	unsat.	NB	NB
**384**	Gala1-3Galb1-3(Fuca1-4)GlcNAcb1-2Mana1-6(Gala1-3Galb1-3(Fuca1-4)GlcNAcb1-2Mana1-3)Manb1-4GlcNAcb1-4GlcNAc-Sp19	NB	unsat.	unsat.
**385**	GlcNAcb1-2Mana1-6(Galb1-4GlcNAcb1-2Mana1-3)Manb1-4GlcNAcb1-4GlcNAc-Sp12	**1.77**	NB	NB
**386**	Galb1-4GlcNAcb1-2Mana1-6(GlcNAcb1-2Mana1-3)Manb1-4GlcNAcb1-4GlcNAc-Sp12	**2.26**	NB	NB
**387**	Neu5Aca2-3Galb1-3GlcNAcb1-3GalNAca-Sp14	**1.93**	NB	NB
**388**	Fuca1-2Galb1-4GlcNAcb1-3GalNAca-Sp14	unsat.	NB	NB
**389**	Galb1-4(Fuca1-3)GlcNAcb1-3GalNAca-Sp14	NB	NB	NB
**390**	GalNAca1-3GalNAcb1-3Gala1-4Galb1-4GlcNAcb-Sp0	**0.34**	NB	**0.43**
**391**	Gala1-4Galb1-3GlcNAcb1-2Mana1-6(Gala1-4Galb1-3GlcNAcb1-2Mana1-3)Manb1-4GlcNAcb1-4GlcNAcb-Sp19	NB	NB	NB
**392**	Gala1-4Galb1-4GlcNAcb1-2Mana1-6(Gala1-4Galb1-4GlcNAcb1-2Mana1-3)Manb1-4GlcNAcb1-4GlcNAcb-Sp24	unsat.	NB	NB
**393**	Gala1-3Galb1-4GlcNAcb1-3GalNAca-Sp14	NB	NB	NB
**394**	Galb1-3GlcNAcb1-6Galb1-4GlcNAcb-Sp0	**2.15**	NB	NB
**395**	Galb1-3GlcNAca1-6Galb1-4GlcNAcb-Sp0	unsat.	NB	NB
**396**	GalNAcb1-3Gala1-6Galb1-4Glcb-Sp8	**2.75**	NB	NB
**397**	Gala1-3(Fuca1-2)Galb1-4(Fuca1-3)Glcb-Sp21	NB	NB	NB
**398**	Galb1-4GlcNAcb1-6(Neu5Aca2-6Galb1-3GlcNAcb1-3)Galb1-4Glc-Sp21	**2.65**	NB	NB
**399**	Galb1-3GalNAcb1-4(Neu5Aca2-8Neu5Aca2-3)Galb1-4Glcb-Sp0	unsat.	NB	NB
**400**	Neu5Aca2-3Galb1-3GalNAcb1-4(Neu5Aca2-8Neu5Aca2-3)Galb1-4Glcb-Sp0	NB	NB	NB
**401**	Gala1-3(Fuca1-2)Galb1-4GlcNAcb1-3GalNAca-Sp14	**0.28**	unsat.	NB
**402**	GalNAca1-3(Fuca1-2)Galb1-4GlcNAcb1-3GalNAca-Sp14	**0.42**	unsat.	unsat.
**403**	GalNAca1-3GalNAcb1-3Gala1-4Galb1-4Glcb-Sp0	**0.31**	NB	**0.46**
**404**	Fuca1-2Galb1-4(Fuca1-3)GlcNAcb1-3GalNAca-Sp14	unsat.	NB	NB
**405**	Gala1-3(Fuca1-2)Galb1-4(Fuca1-3)GlcNAcb1-3GalNAc-Sp14	unsat.	NB	NB

**406**	GalNAca1-3(Fuca1-2)Galb1-4(Fuca1-3)GlcNAcb1-3GalNAc-Sp14	unsat.	NB	NB
**407**	Galb1-4(Fuca1-3)GlcNAcb1-2Mana1-6(Galb1-4(Fuca1-3)GlcNAcb1-2Mana1-3)Manb1-4GlcNAcb1-4(Fuca1-6)GlcNAcb-Sp22	unsat.	NB	NB
**408**	Fuca1-2Galb1-4GlcNAcb1-2Mana1-6(Fuca1-2Galb1-4GlcNAcb1-2Mana1-3)Manb1-4GlcNAcb1-4(Fuca1-6)GlcNAcb-Sp22	**2.06**	unsat.	unsat.
**409**	GlcNAcb1-2(GlcNAcb1-6)Mana1-6(GlcNAcb1-2Mana1-3)Manb1-4GlcNAcb1-4GlcNAcb-Sp19	NB	NB	NB
**410**	Fuca1-2Galb1-3GlcNAcb1-3GalNAc-Sp14	**1.41**	NB	NB
**411**	Gala1-3(Fuca1-2)Galb1-3GlcNAcb1-3GalNAc-Sp14	**0.35**	unsat.	unsat.
**412**	GalNAca1-3(Fuca1-2)Galb1-3GlcNAcb1-3GalNAc-Sp14	**0.32**	NB	unsat.
**413**	Gala1-3Galb1-3GlcNAcb1-3GalNAc-Sp14	**1.1**	NB	NB
**414**	Fuca1-2Galb1-3GlcNAcb1-2Mana1-6(Fuca1-2Galb1-3GlcNAcb1-2Mana1-3)Manb1-4GlcNAcb1-4(Fuca1-6)GlcNAcb-Sp22	**2.84**	unsat.	unsat.
**415**	Gala1-3(Fuca1-2)Galb1-4GlcNAcb1-2Mana1-6(Gala1-3(Fuca1-2)Galb1-4GlcNAcb1-2Mana1-3)Manb1-4GlcNAcb1-4(Fuca1-6)GlcNAcb-Sp22	**0.28**	**7.68**	unsat.
**416**	Galb1-3GlcNAcb1-6(Galb1-3GlcNAcb1-2)Mana1-6(Galb1-3GlcNAcb1-2Mana1-3)Manb1-4GlcNAcb1-4GlcNAcb-Sp19	**0.47**	unsat.	NB
**417**	Galb1-4GlcNAcb1-6(Fuca1-2Galb1-3GlcNAcb1-3)Galb1-4Glc-Sp21	**0.46**	unsat.	NB
**418**	Fuca1-3GlcNAcb1-6(Galb1-4GlcNAcb1-3)Galb1-4Glc-Sp21	**2.92**	NB	NB
**419**	GlcNAcb1-2Mana1-6(GlcNAcb1-4)(GlcNAcb1-2Mana1-3)Manb1-4GlcNAcb1-4GlcNAc-Sp21	NB	NB	NB
**420**	GlcNAcb1-2Mana1-6(GlcNAcb1-4)(GlcNAcb1-4(GlcNAcb1-2)Mana1-3)Manb1-4GlcNAcb1-4GlcNAc-Sp21	NB	NB	NB
**421**	GlcNAcb1-6(GlcNAcb1-2)Mana1-6(GlcNAcb1-4)(GlcNAcb1-2Mana1-3)Manb1-4GlcNAcb1-4GlcNAc-Sp21	NB	NB	NB
**422**	GlcNAcb1-6(GlcNAcb1-2)Mana1-6(GlcNAcb1-4)(GlcNAcb1-4(GlcNAcb1-2)Mana1-3)Manb1-4GlcNAcb1-4GlcNAc-Sp21	NB	NB	NB
**423**	Galb1-4GlcNAcb1-2Mana1-6(GlcNAcb1-4)(Galb1-4GlcNAcb1-2Mana1-3)Manb1-4GlcNAcb1-4GlcNAc-Sp21	unsat.	NB	NB
**424**	Galb1-4GlcNAcb1-2Mana1-6(GlcNAcb1-4)(Galb1-4GlcNAcb1-4(Galb1-4GlcNAcb1-2)Mana1-3)Manb1-4GlcNAcb1-4GlcNAc-Sp21	**0.99**	unsat.	NB
**425**	Galb1-4GlcNAcb1-6(Galb1-4GlcNAcb1-2)Mana1-6(GlcNAcb1-4)(Galb1-4GlcNAcb1-2Mana1-3)Manb1-4GlcNAcb1-4GlcNAc-Sp21	**1.48**	NB	NB

**426**	Galb1-4GlcNAcb1-6(Galb1-4GlcNAcb1-2)Mana1-6(GlcNAcb1-4)(Galb1-4GlcNAcb1-4(Galb1-4GlcNAcb1-2)Mana1-3)Manb1-4GlcNAcb1-4GlcNAc-Sp21	**0.52**	unsat.	NB
**427**	Galb1-4Galb-Sp10	NB	NB	NB
**428**	Galb1-6Galb-Sp10	NB	NB	NB
**429**	Neu5Aca2-3Galb1-4GlcNAcb1-3Galb-Sp8	unsat.	NB	NB
**430**	GalNAcb1-6GalNAcb-Sp8	NB	NB	NB
**431**	(6S)Galb1-3GlcNAcb-Sp0	unsat.	NB	NB
**432**	(6S)Galb1-3(6S)GlcNAc-Sp0	NB	NB	NB
**433**	Fuca1-2Galb1-4 GlcNAcb1-2Mana1-6(Fuca1-2Galb1-4GlcNAcb1-2(Fuca1-2Galb1-4GlcNAcb1-4)Mana1-3)Manb1-4GlcNAcb1-4GlcNAcb-Sp12	unsat.	unsat.	unsat.
**434**	Fuca1-2Galb1-4(Fuca1-3)GlcNAcb1-2Mana1-6(Fuca1-2Galb1-4(Fuca1-3)GlcNAcb1-4(Fuca1-2Galb1-4(Fuca1-3)GlcNAcb1-2)Mana1-3)Manb1-4GlcNAcb1-4GlcNAcb-Sp12	NB	NB	NB
**435**	Galb1-4(Fuca1-3)GlcNAcb1-6GalNAc-Sp14	NB	NB	NB
**436**	Galb1-4GlcNAcb1-2Mana-Sp0	NB	NB	NB
**437**	Fuca1-2Galb1-4GlcNAcb1-6(Fuca1-2Galb1-4GlcNAcb1-3)GalNAc-Sp14	**2.26**	NB	NB
**438**	Gala1-3(Fuca1-2)Galb1-4GlcNAcb1-6(Gala1-3(Fuca1-2)Galb1-4GlcNAcb1-3)GalNAc-Sp14	**0.27**	unsat.	NB
**439**	GalNAca1-3(Fuca1-2)Galb1-4GlcNAcb1-6(GalNAca1-3(Fuca1-2)Galb1-4GlcNAcb1-3)GalNAc-Sp14	**0.26**	unsat.	unsat.
**440**	Neu5Aca2-8Neu5Aca2-3Galb1-3GalNAcb1-4(Neu5Aca2-8Neu5Aca2-3)Galb1-4Glcb-Sp0	**2.96**	NB	NB
**441**	GalNAcb1-4Galb1-4Glcb-Sp0	NB	NB	NB
**442**	GalNAca1-3(Fuca1-2)Galb1-4GlcNAcb1-2Mana1-6(GalNAca1-3(Fuca1-2)Galb1-4GlcNAcb1-2Mana1-3)Manb1-4GlcNAcb1-4(Fuca1-6)GlcNAcb-Sp22	**0.26**	unsat.	unsat.
**443**	Gala1-3(Fuca1-2)Galb1-3GlcNAcb1-2Mana1-6(Gala1-3(Fuca1-2)Galb1-3GlcNAcb1-2Mana1-3)Manb1-4GlcNAcb1-4(Fuca1-6)GlcNAcb-Sp22	**0.34**	unsat.	**4.97**
**444**	Neu5Aca2-6Galb1-4GlcNAcb1-6(Fuca1-2Galb1-3GlcNAcb1-3)Galb1-4Glc-Sp21	**1.73**	NB	NB
**445**	GalNAca1-3(Fuca1-2)Galb1-3GlcNAcb1-2Mana1-6(GalNAca1-3(Fuca1-2)Galb1-3GlcNAcb1-2Mana1-3)Manb1-4GlcNAcb1-4(Fuca1-6)GlcNAcb-Sp22	**0.31**	NB	**0.69**
**446**	Galb1-4GlcNAcb1-6(Galb1-4GlcNAcb1-2)Mana1-6(Galb1-4GlcNAcb1-2Mana1-3)Manb1-4GlcNAcb1-4GlcNAcb-Sp19	**0.5**	unsat.	NB
**447**	Neu5Aca2-3Galb1-4GlcNAcb1-2Mana1-6(GlcNAcb1-4)(Neu5Aca2-3Galb1-4GlcNAcb1-2Mana1-3)Manb1-4GlcNAcb1-4GlcNAcb-Sp21	**1.93**	NB	NB

**448**	Neu5Aca2-3Galb1-4GlcNAcb1-4Mana1-6(GlcNAcb1-4)(Neu5Aca2-3Galb1-4GlcNAcb1-4(Neu5Aca2-3Galb1-4GlcNAcb1-2)Mana1-3)Manb1-4GlcNAcb1-4GlcNAcb-Sp21	**1.91**	NB	NB
**449**	Neu5Aca2-3Galb1-4GlcNAcb1-6(Neu5Aca2-3Galb1-4GlcNAcb1-2)Mana1-6(GlcNAcb1-4)(Neu5Aca2-3Galb1-4GlcNAcb1-2Mana1-3)Manb1-4GlcNAcb1-4GlcNAcb-Sp21	**2.65**	NB	NB
**450**	Neu5Aca2-3Galb1-4GlcNAcb1-6(Neu5Aca2-3Galb1-4GlcNAcb1-2)Mana1-6(GlcNAcb1-4)(Neu5Aca2-3Galb1-4GlcNAcb1-4(Neu5Aca2-3Galb1-4GlcNAcb1-2)Mana1-3)Manb1-4GlcNAcb1-4GlcNAcb-Sp21	**2.16**	NB	NB
**451**	Neu5Aca2-6Galb1-4GlcNAcb1-2Mana1-6(GlcNAcb1-4)(Neu5Aca2-6Galb1-4GlcNAcb1-2Mana1-3)Manb1-4GlcNAcb1-4GlcNAcb-Sp21	NB	NB	NB
**452**	Neu5Aca2-6Galb1-4GlcNAcb1-4Mana1-6(GlcNAcb1-4)(Neu5Aca2-6Galb1-4GlcNAcb1-4(Neu5Aca2-6Galb1-4GlcNAcb1-2)Mana1-3)Manb1-4GlcNAcb1-4GlcNAcb-Sp21	NB	NB	NB
**453**	Neu5Aca2-6Galb1-4GlcNAcb1-6(Neu5Aca2-6Galb1-4GlcNAcb1-2)Mana1-6(GlcNAcb1-4)(Neu5Aca2-6Galb1-4GlcNAcb1-2Mana1-3)Manb1-4GlcNAcb1-4GlcNAcb-Sp21	NB	NB	NB
**454**	Neu5Aca2-6Galb1-4GlcNAcb1-6(Neu5Aca2-6Galb1-4GlcNAcb1-2)Mana1-6(GlcNAcb1-4)(Neu5Aca2-6Galb1-4GlcNAcb1-4(Neu5Aca2-6Galb1-4GlcNAcb1-2)Mana1-3)Manb1-4GlcNAcb1-4GlcNAcb-Sp21	NB	NB	NB
**455**	Gala1-3(Fuca1-2)Galb1-3GalNAca-Sp8	unsat.	NB	NB
**456**	Gala1-3(Fuca1-2)Galb1-3GalNAcb-Sp8	unsat.	NB	NB
**457**	Glca1-6Glca1-6Glca1-6Glcb-Sp10	NB	NB	NB
**458**	Glca1-4Glca1-4Glca1-4Glcb-Sp10	NB	NB	NB
**459**	Neu5Aca2-3Galb1-4GlcNAcb1-6(Neu5Aca2-3Galb1-4GlcNAcb1-3)GalNAca-Sp14	NB	NB	NB
**460**	Fuca1-2Galb1-4(Fuca1-3)GlcNAcb1-2Mana1-6(Fuca1-2Galb1-4(Fuca1-3)GlcNAcb1-2Mana1-3)Manb1-4GlcNAcb1-4(Fuca1-6)GlcNAcb-Sp24	unsat.	NB	NB
**461**	Fuca1-2Galb1-3(Fuca1-4)GlcNAcb1-2Mana1-6(Fuca1-2Galb1-3(Fuca1-4)GlcNAcb1-2Mana1-3)Manb1-4GlcNAcb1-4(Fuca1-6)GlcNAcb1-4(Fuca1-6)GlcNAcb-Sp19	NB	NB	NB
**462**	GlcNAcb1-6(GlcNAcb1-2)Mana1-6(GlcNAcb1-2Mana1-3)Manb1-4GlcNAcb1-4(Fuca1-6)GlcNAcb-Sp24	unsat.	NB	NB
**463**	Galb1-3GlcNAcb1-2Mana1-6(GlcNAcb1-4)(Galb1-3GlcNAcb1-2Mana1-3)Manb1-4GlcNAcb1-4GlcNAcb-Sp21	**1.42**	NB	NB
**464**	Neu5Aca2-6Galb1-4GlcNAcb1-6(Galb1-3GlcNAcb1-3)Galb1-4Glcb-Sp21	**2.72**	NB	NB
**465**	Neu5Aca2-3Galb1-4GlcNAcb1-2Mana-Sp0	NB	NB	NB

**466**	Neu5Aca2-3Galb1-4GlcNAcb1-6GalNAca-Sp14	NB	NB	NB
**467**	Neu5Aca2-6Galb1-4GlcNAcb1-6GalNAca-Sp14	NB	NB	NB
**468**	Neu5Aca2-6Galb1-4 GlcNAcb1-6(Neu5Aca2-6Galb1-4GlcNAcb1-3)GalNAca-Sp14	NB	NB	NB
**469**	Neu5Aca2-6Galb1-4GlcNAcb1-2Mana1-6(Neu5Aca2-6Galb1-4GlcNAcb1-2Mana1-3)Manb1-4GlcNAcb1-4(Fuca1-6)GlcNAcb-Sp24	NB	NB	NB
**470**	Neu5Aca2-3Galb1-4GlcNAcb1-2Mana1-6(Neu5Aca2-3Galb1-4GlcNAcb1-2Mana1-3)Manb1-4GlcNAcb1-4(Fuca1-6)GlcNAcb-Sp24	**2.48**	NB	NB
**471**	Mana1-6(Mana1-3)Manb1-4GlcNAcb1-4(Fuca1-6)GlcNAcb-Sp19	NB	NB	NB
**472**	Galb1-4GlcNAcb1-6(Galb1-4GlcNAcb1-2)Mana1-6(Galb1-4GlcNAcb1-2Mana1-3)Manb1-4GlcNAcb1-4(Fuca1-6)GlcNAcb-Sp24	**0.43**	unsat.	NB
**473**	Neu5Aca2-3Galb1-3GlcNAcb1-2Mana1-6(GlcNAcb1-4)(Neu5Aca2-3Galb1-3GlcNAcb1-2Mana1-3)Manb1-4GlcNAcb1-4GlcNAc-Sp21	NB	NB	NB
**474**	Neu5Aca2-6Galb1-4GlcNAcb1-6(Fuca1-2Galb1-4(Fuca1-3)GlcNAcb1-3)Galb1-4Glc-Sp21	NB	NB	NB
**475**	Galb1-3GlcNAcb1-6GalNAca-Sp14	**1.77**	NB	NB
**476**	Gala1-3Galb1-3GlcNAcb1-6GalNAca-Sp14	**0.53**	NB	NB
**477**	Galb1-3(Fuca1-4)GlcNAcb1-6GalNAca-Sp14	unsat.	NB	NB
**478**	Neu5Aca2-3Galb1-3GlcNAcb1-6GalNAca-Sp14	unsat.	NB	NB
**479**	(3S)Galb1-3(Fuca1-4)GlcNAcb-Sp0	NB	NB	NB
**480**	Galb1-4(Fuca1-3)GlcNAcb1-6(Neu5Aca2-6(Neu5Aca2-3Galb1-3)GlcNAcb1-3)Galb1-4Glc-Sp21	unsat.	NB	NB
**481**	Fuca1-2Galb1-4GlcNAcb1-6GalNAca-Sp14	unsat.	NB	NB
**482**	Gala1-3Galb1-4GlcNAcb1-6GalNAca-Sp14	**1.29**	NB	NB
**483**	Galb1-4(Fuca1-3)GlcNAcb1-2Mana-Sp0	NB	NB	NB
**484**	Fuca1-2(6S)Galb1-3GlcNAcb-Sp0	NB	NB	NB
**485**	Gala1-3(Fuca1-2)Galb1-4GlcNAcb1-6GalNAca-Sp14	NB	NB	NB
**486**	Fuca1-2Galb1-4GlcNAcb1-2Mana-Sp0	NB	NB	NB
**487**	Fuca1-2Galb1-3(6S)GlcNAcb-Sp0	**2.15**	NB	NB
**488**	Fuca1-2(6S)Galb1-3(6S)GlcNAcb-Sp0	unsat.	NB	NB
**489**	Neu5Aca2-6GalNAcb1-4(6S)GlcNAcb-Sp8	unsat.	NB	NB
**490**	GalNAcb1-4(Fuca1-3)(6S)GlcNAcb-Sp8	NB	NB	NB
**491**	(3S)GalNAcb1-4(Fuca1-3)GlcNAcb-Sp8	NB	NB	NB
**492**	Fuca1-2Galb1-3GlcNAcb1-6(Fuca1-2Galb1-3GlcNAcb1-3)GalNAca-Sp14	**1.83**	NB	NB
**493**	GalNAca1-3(Fuca1-2)Galb1-3GlcNAcb1-6GalNAca-Sp14	**0.27**	unsat.	**2.93**

**494**	GlcNAcb1-6(GlcNAcb1-2)Mana1-6(GlcNAcb1-4)(GlcNAcb1-4(GlcNAcb1-2)Mana1-3)Manb1-4GlcNAcb1-4(Fuca1-6)GlcNAc-Sp21	NB	NB	NB
**495**	Galb1-4GlcNAcb1-6(Galb1-4GlcNAcb1-2)Mana1-6(GlcNAcb1-4)Galb1-4GlcNAcb1-4(Gal b1-4GlcNAcb1-2)Mana1-3)Manb1-4GlcNAcb1-4(Fuca1-6)GlcNAc-Sp21	**0.32**	unsat.	NB
**496**	Galb1-3GlcNAca1-3Galb1-4GlcNAcb-Sp8	**0.33**	unsat.	unsat.
**497**	Galb1-3(6S)GlcNAcb-Sp8	NB	NB	NB
**498**	(6S)(4S)GalNAcb1-4GlcNAc-Sp8	NB	NB	NB
**499**	(6S)GalNAcb1-4GlcNAc-Sp8	NB	NB	NB
**500**	(3S)GalNAcb1-4(3S)GlcNAc-Sp8	unsat.	NB	NB
**501**	GalNAcb1-4(6S)GlcNAc-Sp8	**1.82**	NB	NB
**502**	(3S)GalNAcb1-4GlcNAc-Sp8	**1.14**	unsat.	NB
**503**	(4S)GalNAcb-Sp10	NB	NB	NB
**504**	Galb1-4(6P)GlcNAcb-Sp0	NB	NB	NB
**505**	(6P)Galb1-4GlcNAcb-SP0	NB	NB	NB
**506**	GalNAca1-3(Fuca1-2)Galb1-4GlcNAcb1-6GalNAc-Sp14	**0.43**	unsat.	unsat.
**507**	Neu5Aca2-6Galb1-4GlcNAcb1-2Man-Sp0	NB	NB	NB
**508**	Gala1-3Galb1-4GlcNAcb1-2Mana-Sp0	**0.45**	unsat.	NB
**509**	Gala1-3(Fuca1-2)Galb1-4GlcNAcb1-2Mana-Sp0	**0.29**	**8.81**	NB
**510**	GalNAca1-3(Fuca1-2)Galb1-4 GlcNAcb1-2Mana-Sp0	**0.24**	unsat.	**2.64**
**511**	Galb1-3GlcNAcb1-2Mana-Sp0	**3.01**	NB	NB
**512**	Gala1-3(Fuca1-2)Galb1-3GlcNAcb1-6GalNAc-Sp14	**0.3**	unsat.	unsat.
**513**	Neu5Aca2-3Galb1-3GlcNAcb1-2Mana-Sp0	unsat.	NB	NB
**514**	Gala1-3Galb1-3GlcNAcb1-2Mana-Sp0	**0.47**	unsat.	NB
**515**	GalNAcb1-4GlcNAcb1-2Mana-Sp0	**1.97**	NB	NB
**516**	Neu5Aca2-3Galb1-3GlcNAcb1-4Galb1-4Glcb-Sp0	NB	NB	NB
**517**	GlcNAcb1-2 Mana1-6(GlcNAcb1-4)(GlcNAcb1-2Mana1-3)Manb1-4GlcNAcb1-4(Fuca1-6)GlcNAc-Sp21	NB	NB	NB
**518**	Galb1-4GlcNAcb1-2 Mana1-6(GlcNAcb1-4)(Galb1-4GlcNAcb1-2Mana1-3)Manb1-4GlcNAcb1-4(Fuca1-6)GlcNAc-Sp21	**0.4**	unsat.	NB
**519**	Galb1-4GlcNAcb1-2 Mana1-6(Galb1-4GlcNAcb1-4)(Galb1-4GlcNAcb1-2Mana1-3)Manb1-4GlcNAcb1-4(Fuca1-6)GlcNAc-Sp21	**0.52**	unsat.	NB
**520**	Fuca1-4(Galb1-3)GlcNAcb1-2 Mana-Sp0	NB	NB	NB
**521**	Neu5Aca2-3Galb1-4(Fuca1-3)GlcNAcb1-2Mana-Sp0	NB	NB	NB
**522**	GlcNAcb1-3Galb1-4GlcNAcb1-6(GlcNAcb1-3)Galb1-4GlcNAc-Sp0	**0.32**	unsat.	unsat.
**523**	GalNAca1-3(Fuca1-2)Galb1-3GalNAcb1-3Gala1-4Galb1-4Glc-Sp21	**1.97**	NB	unsat.

**524**	Gala1-3(Fuca1-2)Galb1-3GalNAcb1-3Gala1-4Galb1-4Glc-Sp21	unsat.	NB	NB
**525**	Galb1-3GalNAcb1-3Gal-Sp21	**1.44**	NB	NB
**526**	GlcNAcb1-3Galb1-4GlcNAcb1-2Mana1-6(GlcNAcb1-3Galb1-4GlcNAcb1-2Mana1-3)Manb1-4GlcNAcb1-4GlcNAcb-Sp12	**0.28**	unsat.	unsat.
**527**	GlcNAcb1-3Galb1-4GlcNAcb1-2Mana1-6(GlcNAcb1-3Galb1-4GlcNAcb1-2Mana1-3)Manb1-4GlcNAcb1-4GlcNAcb-Sp25	**0.23**	unsat.	unsat.
**528**	Galb1-4GlcNAcb1-3Galb1-4GlcNAcb1-2Mana1-6(Galb1-4GlcNAcb1-3Galb1-4GlcNAcb1-2Mana1-3)Manb1-4GlcNAcb1-4GlcNAcb-Sp12	**2.26**	unsat.	unsat.
**529**	Fuca1-2Galb1-4GlcNAcb1-3Galb1-4GlcNAcb1-2Mana1-6(Fuca1-2Galb1-4GlcNAcb1-3Galb1-4GlcNAcb1-2Mana1-3)Manb1-4GlcNAcb1-4GlcNAcb-Sp24	**0.27**	unsat.	unsat.
**530**	GlcNAcb1-3Galb1-4GlcNAcb1-3Galb1-4GlcNAcb1-2Mana1-6(GlcNAcb1-3Galb1-4GlcNAcb1-3Galb1-4GlcNAcb1-2Mana1-3)Manb1-4GlcNAcb1-4GlcNAcb-Sp12	**0.42**	unsat.	unsat.
**531**	Galb1-4GlcNAcb1-3Galb1-4GlcNAcb1-3Galb1-4GlcNAcb1-2Mana1-6(Galb1-4GlcNAcb1-3Galb1-4GlcNAcb1-3Galb1-4GlcNAcb1-2Mana1-3)Manb1-4GlcNAcb1-4GlcNAcb-Sp12	**0.25**	**5.18**	**7.64**
**532**	Galb1-3GlcNAcb1-3Galb1-4GlcNAcb1-2Mana1-6(Galb1-3GlcNAcb1-3Galb1-4GlcNAcb1-2Mana1-3)Manb1-4GlcNAcb1-4GlcNAc-Sp25	**0.27**	unsat.	unsat.
**533**	Neu5Gca2-8Neu5Gca2-3Galb1-4GlcNAc-Sp0	unsat.	NB	NB
**534**	Neu5Aca2-8Neu5Gca2-3Galb1-4GlcNAc-Sp0	**2.36**	NB	NB
**535**	Neu5Gca2-8Neu5Aca2-3Galb1-4GlcNAc-Sp0	unsat.	NB	NB
**536**	Neu5Gca2-8Neu5Gca2-3Galb1-4GlcNAcb1-3Galb1-4GlcNAc-Sp0	**2.41**	NB	NB
**537**	Neu5Gca2-8Neu5Gca2-6Galb1-4GlcNAc-Sp0	NB	NB	NB
**538**	Neu5Aca2-8Neu5Aca2-3Galb1-4GlcNAc-Sp0	NB	NB	NB
**539**	GlcNAcb1-3Galb1-4GlcNAcb1-6(GlcNAcb1-3Galb1-4GlcNAcb1-2)Mana1-6(GlcNAcb1-3Galb1-4GlcNAcb1-2Man a1-3)Manb1-4GlcNAcb1-4GlcNAc-Sp24	**0.27**	**7.25**	unsat.
**540**	Galb1-4GlcNAcb1-3Galb1-4GlcNAcb1-6(Galb1-4GlcNAcb1-3Galb1-4GlcNAcb1-2)Mana1-6(Galb1-4GlcNAcb1-3Galb1-4GlcNAcb1-2Mana1-3)Manb1-4GlcNAcb1-4GlcNAc-Sp24	**0.36**	**7.48**	**7.61**
**541**	Gala1-3Galb1-4GlcNAcb1-2Mana1-6(Gala1-3Galb1-4GlcNAcb1-2Mana1-3)Manb1-4GlcNAcb1-4GlcNAc-Sp24	**0.29**	unsat.	unsat.
**542**	GlcNAcb1-3Galb1-4GlcNAcb1-6(GlcNAcb1-3Galb1-3)GalNAca-Sp14	**0.69**	NB	unsat.
**543**	GalNAcb1-3GlcNAcb-Sp0	**1.01**	unsat.	NB
**544**	GalNAcb1-4GlcNAcb1-3GalNAcb1-4GlcNAcb-Sp0	**0.92**	unsat.	NB
**545**	GlcNAb1-3Galb1-3GalNAc-Sp14	unsat.	NB	NB

**546**	Galb1-3GlcNAcb1-6(Galb1-3)GalNAc-Sp14	**0.24**	**8.69**	unsat.
**547**	(3S)GlcAb1-3Galb1-4GlcNAcb1-3Galb1-4Glc-Sp0	**1.46**	NB	unsat.
**548**	(3S)GlcAb1-3Galb1-4GlcNAcb1-2Mana-Sp0	unsat.	NB	NB
**549**	Galb1-3GlcNAcb1-3Galb1-4GlcNAcb1-3Galb1-4GlcNAcb1-6(Galb1-3GlcNAcb1-3Galb1-4GlcNAcb1-3Galb1-4GlcNAb1-2)Mana1-6(Galb1-3GlcNAcb1-3Galb1-4GlcNAcb1-3Galb1-4GlcNAcb1-2Mana1-3)Manb1-4GlcNAcb1-4(Fuca1-6)GlcNAcb-Sp24	**0.29**	**4.03**	unsat.
**550**	Galb1-3GlcNAcb1-3Galb1-4GlcNAcb1-6(Galb1-3GlcNAcb1-3Galb1-4GlcNAb1-2)Mana1-6(Galb1-3GlcNAcb1-3Galb1-4GlcNAcb1-2Mana1-3)Manb1-4GlcNAcb1-4(Fuca1-6)GlcNAcb-Sp24	**0.27**	**7.51**	unsat.
**551**	Galb1-4GlcNAcb1-3Galb1-4GlcNAcb1-2Mana1-6(Galb1-4GlcNAcb1-3Galb1-4GlcNAcb1-2Mana1-3)Manb1-4GlcNAcb1-4(Fuca1-6)GlcNAcb-Sp24	**0.23**	**4.65**	**2.57**
**552**	Galb1-4GlcNAcb1-3Galb1-4GlcNAcb1-3GalNAca-Sp14	**0.39**	unsat.	unsat.
**553**	Galb1-4GlcNAcb1-3Galb1-4GlcNAcb1-6(Galb1-3)GalNAca-Sp14	**0.44**	unsat.	unsat.
**554**	Galb1-4GlcNAcb1-3Galb1-4GlcNAcb1-6(Galb1-4GlcNAcb1-3Galb1-4GlcNAcb1-3)GalNAca-Sp14	**0.41**	unsat.	unsat.
**555**	GlcNAcb1-3Galb1-4GlcNAcb1-3GalNAca-Sp14	**0.74**	NB	NB
**556**	GlcNAcb1-3Galb1-4GlcNAcb1-6(Galb1-3)GalNAca-Sp14	**1.13**	NB	NB
**557**	GlcNAcb1-3Galb1-4GlcNAcb1-6(GlcNAcb1-3Galb1-4GlcNAcb1-3)GalNAca-Sp14	**0.45**	unsat.	NB
**558**	GlcNAcb1-3Galb1-4GlcNAcb1-3Galb1-4GlcNAcb1-3GalNAca-Sp14	**0.29**	unsat.	unsat.
**559**	Galb1-4GlcNAcb1-3Galb1-3GalNAca-Sp14	**0.42**	NB	unsat.
**560**	Neu5Aca2-6Galb1-4GlcNAcb1-6(Galb1-3)GalNAca-Sp14	unsat.	NB	NB
**561**	GlcNAcb1-6(Neu5Aca2-3Galb1-3)GalNAca-Sp14	unsat.	NB	NB
**562**	Galb1-3GalNAcb1-4(Neu5Aca2-8Neu5Aca2-8Neu5Aca2-3)Galb1-4Glcb-Sp21	NB	NB	NB
