# Supplementary material for: Galectin-9 recognizes and exhibits antimicrobial activity toward microbes expressing blood group–like antigens
Source: J Biol Chem. 2022 Feb 9;298(4):101704. doi: 10.1016/j.jbc.2022.101704 (PMC9019251; doi:10.1016/j.jbc.2022.101704)
Supplement: Supplemental Table S2 [file mmc3.pdf]

Table S2. Table of apparent Kd values of Gal-9, Gal-9C and Gal-9N binding to glycans on MGM array

NB = no binding   unsat. = unsaturated binding (Kd cannot be estimated)

Glycan #	BACTERIA STRAIN	Gal-9 Kd	Gal-9C Kd	Gal-9N Kd
1	Providencia stuartii O49	NB	NB	NB
2	Providencia stuartii O52	Unsat.	NB	NB
3	Pseudomonas aerguinosa O4 (Habs serotype 4)	NB	NB	NB
4	Pseudomonas aerguinosa O1 (Fisher immunotype 4)	NB	NB	NB
5	Pseudomonas aerguinosa O2 (Fisher immunotype 3)	Unsat.	NB	NB
6	Pseudomonas aerguinosa O13 (Sandvik serotype II)	NB	NB	NB
7	Pseudomonas aerguinosa O9 (9a, 9b, 9d)	Unsat.	unsat.	NB
8	Pseudomonas aerguinosa O6a (Habs serotype6, fraction IIa)	NB	NB	NB
9	Pseudomonas aerguinosa O6a (Habs serotype6, fraction IIb)	NB	NB	NB
10	Salmonella typhimurium SL 11881 (Re mut)	Unsat.	Unsat.	NB
11	Salmonella typhimurium TV 119 (Ra mut)	NB	NB	NB
12	Salmonella typhimurium SL 684 (Rc mut)	NB	NB	NB
13	Pseudomonas aerguinosa O10	Unsat.	NB	NB
14	Salmonella typhimurium dodeca saccharide	NB	NB	NB
15	Salmonella enteritidis dodeca saccharide	NB	NB	NB
16	Salmonella typhimurium LPS	**3.4**	**8.76**	NB
17	Serratia marcescens LPS	NB	NB	NB
18	Escherichia coli K235 LPS	NB	unsat	NB
19	Escherichia coli O128-B12 LPS	**2.31**	unsat.	NB
20	Salmonella enterica abortus equi LPS	Unsat.	unsat.	unsat.
21	Salmonella typhosa LPS	Unsat.	unsat.	NB
22	Salmonella enteritidis LPS	Unsat.	unsat.	NB
23	Shigella bodyii type2	Unsat.	unsat.	NB
24	Shigella bodyii type4	Unsat.	NB	NB
25	Shigella bodyii type10	NB	NB	NB
26	Shigella dysennteriae type 3	Unsat.	NB	NB
27	Shigella dysennteriae type 8 (batch 12)	NB	NB	NB
28	Shigella dysennteriae type 11	Unsat.	NB	NB
29	Shigella dysennteriae type 13	NB	NB	NB
30	Escherichia coli O29	NB	NB	NB
31	Escherichia coli O40	**1.23**	unsat.	NB
32	Escherichia coli O106	Unsat.	NB	NB
33	Escherichia coli O130	NB	NB	NB
34	Escherichia coli O148	NB	NB	NB
35	Escherichia coli O150	NB	NB	NB
36	Escherichia coli O180	**3.65**	NB	NB
37	Proteus mirabilis O3a, 3c (G1)	NB	NB	NB
38	Proteus mirabilis O8 (TG326)	Unsat.	NB	NB
39	Proteus mirabilis O10 (HJ4320)	Unsat.	NB	NB
40	Proteus mirabilis O29a, 29b (2002)	NB	NB	NB
41	Proteus mirabilis O50 (TG332)	NB	NB	NB
42	Proteus mirabilis O54a, 54b (10704)	Unsat.	NB	NB
43	Proteus mirabilis O57 (TG319)	NB	NB	NB
44	Proteus penneri O8 (106)	NB	NB	NB
45	Proteus penneri O64a, 64b, 64d (39)	NB	NB	NB
46	Proteus penneri O66 (2)	NB	NB	NB
47	Proteus penneri O69 (25)	Unsat.	NB	NB

**48**	Proteus penneri O71 (42)	Unsat.	NB	NB
**49**	Proteus penneri O72a, 72b (4)	Unsat.	NB	NB
**50**	Pseudomonas aerguinosa O2 (2a),2d,2f	Unsat.	NB	NB
**51**	Pseudomonas aerguinosa O2 2a,2b	Unsat.	NB	NB
**52**	Pseudomonas aerguinosa O2 2a,2b,2e	Unsat.	NB	NB
**53**	Pseudomonas aerguinosa O2 2a,2d	Unsat.	NB	NB
**54**	Pseudomonas aerguinosa O2 Immuno 7	NB	NB	NB
**55**	Pseudomonas aerguinosa O3 3a,3b	Unsat.	NB	NB
**56**	Pseudomonas aerguinosa O3 3a,3b,3c	Unsat.	NB	Unsat.
**57**	Pseudomonas aerguinosa O3 3a,3d	NB	NB	NB
**58**	Pseudomonas aerguinosa O4 4a,4c	NB	NB	NB
**59**	Pseudomonas aerguinosa O6 6a	Unsat.	NB	NB
**60**	Pseudomonas aerguinosa O6 6a,6c	NB	NB	NB
**61**	Pseudomonas aerguinosa O6 Immuno 1	Unsat.	NB	NB
**62**	Pseudomonas aerguinosa O7 7a,7b,7c	Unsat.	NB	NB
**63**	Pseudomonas aerguinosa O7 7a,7b,7d	NB	NB	NB
**64**	Pseudomonas aerguinosa O7 7a,7d	Unsat.	NB	NB
**65**	Pseudomonas aerguinosa O10 10a,10b	NB	NB	NB
**66**	Pseudomonas aerguinosa O10 10a,10c	Unsat.	NB	NB
**67**	Pseudomonas aerguinosa O11 11a,11b	NB	NB	NB
**68**	Pseudomonas aerguinosa O12 12	Unsat.	NB	NB
**69**	Pseudomonas aerguinosa O13 13a,13c	NB	NB	NB
**70**	Pseudomonas aerguinosa O14 14	Unsat.	NB	NB
**71**	Pseudomonas aerguinosa O15 15	Unsat.	NB	NB
**72**	Proteus vulgaris O1 (18984)*	Unsat.	unsat.	NB
**73**	Proteus vulgaris O4 (PrK 9/57)	NB	NB	NB
**74**	Proteus vulgaris O12 (PrK 25/57)	NB	NB	NB
**75**	Proteus vulgaris O13 (8344)	NB	NB	NB
**76**	Proteus vulgaris O15 (PrK 30/57)	NB	NB	NB
**77**	Proteus vulgaris O17 (PrK 33/57)	NB	NB	NB
**78**	Proteus vulgaris O19a (PrK 37/57)	Unsat.	NB	NB
**79**	Proteus vulgaris O21 (PrK 39/57)*	Unsat.	unsat.	unsat.
**80**	Proteus vulgaris O25 (PrK 48/57)	Unsat.	NB	NB
**81**	Proteus vulgaris O34 (4669)*	Unsat.	NB	NB
**82**	Proteus vulgaris O37a,b (PrK 63/57)	Unsat.	NB	unsat.
**83**	Proteus vulgaris O37a,c (PrK 72/57)	Unsat.	NB	NB
**84**	Proteus vulgaris O44 (PrK 67/57)	Unsat.	NB	NB
**85**	Proteus vulgaris O45 (4680)	**0.34**	NB	**0.15**
**86**	Proteus vulgaris O53 (TG 276-10)	Unsat.	NB	NB
**87**	Proteus vulgaris O54a,54c (TG 103)	Unsat.	NB	unsat.
**88**	Proteus vulgaris O55 (TG 155)	**3.28**	unsat.	NB
**89**	Proteus vulgaris O65 (TG 251)	**2.96**	NB	unsat.
**90**	Proteus mirabilis O6 (PrK 14/57)	NB	NB	NB
**91**	Proteus mirabilis O11 (PrK 24/57)	NB	unsat.	NB
**92**	Proteus mirabilis O13 (PrK 26/57)	NB	unsat.	NB
**93**	Proteus mirabilis O14a,14b (PrK 29/57)	NB	NB	NB
**94**	Proteus mirabilis O16 (4652)	Unsat.	NB	NB
**95**	Proteus mirabilis O17 (PrK 32/57)	Unsat.	NB	NB

**96**	Proteus mirabilis O23a,b,d (PrK 42/57)	NB	NB	NB
**97**	Proteus mirabilis O26 (PrK 49/57)	Unsat.	NB	NB
**98**	Proteus mirabilis O27 (PrK 50/57)	NB	NB	NB
**99**	Proteus mirabilis O28 (PrK 51/57)	Unsat.	NB	NB
**100**	Proteus mirabilis O29a (PrK 52/57)	Unsat.	NB	unsat.
**101**	Proteus mirabilis O40 (10703)	NB	NB	NB
**102**	Proteus mirabilis O41 (PrK 67/57)	NB	NB	NB
**103**	Proteus mirabilis O51 (19011)*	Unsat.	NB	NB
**104**	Proteus mirabilis O74 (10705, OF)	Unsat.	Unsat.	NB
**105**	Proteus mirabilis O75 (10702, OC)	NB	NB	NB
**106**	Proteus mirabilis O77 (3 B-m)	NB	NB	NB
**107**	Proteus penneri O31a (26)	NB	NB	NB
**108**	Proteus penneri O52 (15)	NB	NB	NB
**109**	Proteus penneri O58 (12)	NB	NB	NB
**110**	Proteus penneri O59 (9)	Unsat.	unsat.	NB
**111**	Proteus penneri O61 (21)	NB	NB	NB
**112**	Proteus penneri O62 (41)	NB	NB	NB
**113**	Proteus penneri O63 (22)	Unsat.	NB	NB
**114**	Proteus penneri O64a,b,c (27)	NB	NB	NB
**115**	Proteus penneri O65 (34)	**1.48**	NB	unsat.
**116**	Proteus penneri O67 (8)	Unsat.	NB	NB
**117**	Proteus penneri O68 (63)	Unsat.	NB	NB
**118**	Proteus penneri O70 (60)	Unsat.	NB	NB
**119**	Proteus penneri O73a,b (103)	**1.24**	NB	unsat.
**120**	Proteus myxofaciens O60	Unsat.	NB	NB
**121**	Proteus O56 (genomospecies 4)	Unsat.	NB	NB
**122**	Providencia stuartii O4	Unsat.	NB	NB
**123**	Providencia stuartii O18	NB	NB	NB
**124**	Providencia stuartii O20*	Unsat.	NB	NB
**125**	Providencia stuartii O43	NB	NB	NB
**126**	Providencia stuartii O44	Unsat.	unsat.	NB
**127**	Providencia stuartii O47	Unsat.	NB	NB
**128**	Providencia stuartii O47, Core 9	Unsat.	NB	NB
**129**	Providencia stuartii O49, Core 1	Unsat.	NB	NB
**130**	Providencia stuartii O57	Unsat.	NB	NB
**131**	Providencia alcalifaciens O5	**1.03**	**2.5**	**1.88**
**132**	Providencia alcalifaciens O6*	**0.48**	**10.4**	**1.22**
**133**	Providencia alcalifaciens O19	Unsat.	NB	NB
**134**	Providencia alcalifaciens O19	Unsat.	NB	NB
**135**	Providencia alcalifaciens O19	NB	NB	NB
**136**	Providencia alcalifaciens O21	NB	NB	NB
**137**	Providencia alcalifaciens O23	Unsat.	NB	unsat.
**138**	Providencia alcalifaciens O27	NB	unsat.	NB
**139**	Providencia alcalifaciens O29	Unsat.	NB	NB
**140**	Providencia alcalifaciens O30	Unsat.	unsat.	NB
**141**	Providencia alcalifaciens O32	NB	NB	NB
**142**	Providencia alcalifaciens O36*	Unsat.	NB	NB
**143**	Providencia alcalifaciens O39	Unsat.	NB	NB

**144**	Providencia rustigianii O14	NB	NB	NB
**145**	Providencia rustigianii O16	Unsat.	NB	NB
**146**	Providencia rustigianii O34	Unsat.	NB	NB
**147**	Yersinia pestis, KM260(11)-Δ0187	Unsat.	Unsat.	NB
**148**	Yersinia pestis, KM260(11)-Δ0187	NB	NB	NB
**149**	Yersinia pestis, KM260(11)-Δrfe	Unsat.	NB	NB
**150**	Yersinia pestis, KM260(11)-Δrfe	Unsat.	NB	NB
**151**	Yersinia pestis, 1146-25	Unsat.	NB	NB
**152**	Yersinia pestis 1146-25	NB	NB	NB
**153**	Yersinia pestis, 1146-37	NB	NB	NB
**154**	Yersinia pestis, 1146-37	NB	NB	NB
**155**	Yersinia pestis, 0KM218-37	Unsat.	NB	NB
**156**	Yersinia pestis, KM218-37	NB	NB	NB
**157**	Yersinia pestis, KM218-25	Unsat.	NB	NB
**158**	Yersinia pestis, KM218-25	Unsat.	NB	NB
**159**	Yersinia pestis, KM260(11)-ΔpmrF	Unsat.	NB	NB
**160**	Yersinia pestis, KM260(11)-ΔpmrF	NB	NB	NB
**161**	Yersinia pestis, KM260(11)-Δ0186	Unsat.	NB	NB
**162**	Yersinia pestis, KM260(11)-Δ0186	Unsat.	NB	NB
**163**	Yersinia pestis, KM260(11)-ΔwaaQ	Unsat.	NB	NB
**164**	Yersinia pestis, KM260(11)-ΔwaaQ	Unsat.	NB	NB
**165**	Yersinia pestis, KM260(11)-ΔwaaL	Unsat.	NB	NB
**166**	Yersinia pestis, KM260(11)-25	Unsat.	NB	NB
**167**	Yersinia pestis, KM260(11)-25	NB	NB	NB
**168**	Yersinia pestis, KM260(11)-37	NB	NB	NB
**169**	Yersinia pestis, KIMD1-37	NB	NB	NB
**170**	Yersinia pestis, KIMD1-25	NB	NB	NB
**171**	Yersinia pestis, 11M-25	Unsat.	NB	NB
**172**	Yersinia pestis, 11M-37	Unsat.	NB	NB
**173**	Proteus mirabilis O23a, 23b, 23c (CCUG 10701)	NB	NB	NB
**174**	Proteus vulgaris O24 (PrK 47/57)	NB	NB	NB
**175**	Yersinia pestis KM260(11)-6C	Unsat.	unsat.	NB
**176**	Yersinia pestis 260(11)-37C-186	**1.79**	NB	NB
**177**	Yersinia pestis 260(11)-37C-187	Unsat.	NB	NB
**178**	Yersinia pestis 260(11)-37C-416	Unsat.	NB	NB
**179**	Yersinia pestis 260(11)-37C-417	Unsat.	NB	NB
**180**	Yersinia pestis P-1680-25C	NB	NB	NB
**181**	Yersinia pestis P-1680-37C	Unsat.	NB	NB
**182**	Yersinia pestis I-2377-25C	NB	NB	NB
**183**	Yersinia pestis I-2377-37C	Unsat.	NB	NB
**184**	Francisella novicida OPS	NB	NB	NB
**185**	Francisella tularensis OPS	NB	NB	NB
**186**	Klebsiella O1 OPS	**0.26**	unsat.	unsat.
**187**	Klebsiella O2a OPS	**3.42**	**7.11**	unsat.
**188**	Klebsiella O2ac OPS	**5.65**	NB	NB
**189**	Klebsiella O3 OPS	NB	NB	NB
**190**	Klebsiella O4 OPS	NB	NB	NB
**191**	Klebsiella O5 OPS	Unsat.	NB	Unsat.

**192**	Klebsiella O8 OPS	**0.23**	**5.3**	**2.2**
**193**	Klebsiella O12 OPS	Unsat.	NB	NB
**194**	Shigella boydii type 1	Unsat.	Unsat.	NB
**195**	Shigella boydii type 3	NB	NB	NB
**196**	Shigella boydii type 5	Unsat.	unsat.	NB
**197**	Shigella boydii type 9	**3.24**	unsat.	unsat.
**198**	Shigella boydii type 11	Unsat.	NB	NB
**199**	Shigella boydii type 12	**2.28**	unsat.	unsat.
**200**	Shigella boydii type 15	NB	NB	NB
**201**	Shigella boydii type 16	Unsat.	NB	NB
**202**	Shigella boydii type 17	Unsat.	NB	unsat.
**203**	Shigella boydii type 18	Unsat.	NB	NB
**204**	Escherichia coli O49	NB	NB	NB
**205**	Escherichia coli O52	Unsat.	NB	NB
**206**	Escherichia coli O58	Unsat.	NB	NB
**207**	Escherichia coli O61	NB	NB	NB
**208**	Escherichia coli O73	NB	NB	NB
**209**	Escherichia coli O112ab	Unsat.	NB	NB
**210**	Escherichia coli O118	Unsat.	NB	NB
**211**	Escherichia coli O125	Unsat.	NB	NB
**212**	Escherichia coli O151	Unsat.	NB	NB
**213**	Escherichia coli O168	Unsat.	NB	NB
**214**	Shigella dysenteriae type 2	Unsat.	NB	NB
**215**	Shigella dysenteriae type 4	NB	NB	NB
**216**	Shigella dysenteriae type 5	Unsat.	NB	NB
**217**	Shigella dysenteriae type 6 SR-strain	Unsat.	NB	NB
**218**	Shigella dysenteriae type 7	Unsat.	NB	NB
**219**	Shigella dysenteriae type 8 (Russian)	NB	NB	NB
**220**	Shigella dysenteriae type 9	Unsat.	unsat.	NB
**221**	Escherichia coli O111:B4 LPS- solution at 1 mg/mL	Unsat.	NB	unsat.
**222**	Escherichia coli O26:B6 LPS- solution at 1 mg/mL	NB	NB	NB
**223**	Escherichia coli O55:B5 LPS- solution at 1 mg/mL	**0.56**	**3.66**	NB
**224**	Escherichia coli O127:B8 LPS- solution at 1 mg/mL	Unsat.	unsat.	NB
**225**	Streptococcus pneumoniae type 1 (Danish type 1)	Unsat.	NB	NB
**226**	Streptococcus pneumoniae type 2 (Danish type 2)	Unsat.	NB	NB
**227**	Streptococcus pneumoniae type 3 (Danish type 3)	Unsat.	NB	NB
**228**	Streptococcus pneumoniae type 4 (Danish type 4)	Unsat.	NB	NB
**229**	Streptococcus pneumoniae type 5 (Danish type 5)	NB	NB	NB
**230**	Streptococcus pneumoniae type 8 (Danish type 8)	Unsat.	NB	NB
**231**	Streptococcus pneumoniae type 9 (Danish type 9N)	Unsat.	NB	NB
**232**	Streptococcus pneumoniae type 12 (Danish type 12F)	Unsat.	NB	NB
**233**	Streptococcus pneumoniae type 14 (Danish type 14)	**0.31**	**5.37**	NB
**234**	Streptococcus pneumoniae type 17 (Danish type 17F)	Unsat.	NB	NB
**235**	Streptococcus pneumoniae type 19 (Danish type 19F)	Unsat.	NB	NB
**236**	Streptococcus pneumoniae type 20 (Danish type 20)	Unsat.	NB	NB
**237**	Streptococcus pneumoniae type 22 (Danish type 22F)	Unsat.	NB	NB
**238**	Streptococcus pneumoniae type 23 (Danish type 23F)	Unsat.	NB	NB
**239**	Streptococcus pneumoniae type 26 (Danish type 6B)	Unsat.	unsat.	NB

**240**	Streptococcus pneumoniae type 34 (Danish type 10A)	Unsat.	NB	NB
**241**	Streptococcus pneumoniae type 43 (Danish type 11A)	**0.56**	unsat.	**0.89**
**242**	Streptococcus pneumoniae type 51 (Danish type 7F)	Unsat.	NB	NB
**243**	Streptococcus pneumoniae type 54 (Danish type 15B)	**1.16**	unsat.	unsat.
**244**	Streptococcus pneumoniae type 56 (Danish type 18C)	Unsat.	NB	NB
**245**	Streptococcus pneumoniae type 57 (Danish type 19A)	NB	NB	NB
**246**	Streptococcus pneumoniae type 68 (Danish type 9V)	Unsat.	NB	NB
**247**	Streptococcus pneumoniae type 70 (Danish type 33F)	**1.31**	unsat.	NB
**248**	Yersinia pestis KM218-6C	NB	NB	NB
**249**	Yersinia pestis KM260(11)-yjhW-6C	NB	NB	NB
**250**	Yersinia pestis KM260(11)-wabD/waaL	NB	NB	NB
**251**	Yersinia pestis KM260(11)-wabC/waaL	Unsat.	Unsat.	NB
**252**	Yersinia pseudotuberculosis 85pCad-37C	NB	NB	NB
**253**	Yersinia pseudotuberculosis 85pCad-20C	NB	NB	NB
**254**	Yersinia pseudotuberculosis O:2a	NB	NB	NB
**255**	Yersinia pseudotuberculosis O:2a-dhmA	NB	NB	NB
**256**	Yersinia pseudotuberculosis O:2c	Unsat.	NB	NB
**257**	Yersinia pseudotuberculosis O:3	NB	NB	NB
**258**	Yersinia pseudotuberculosis O:4b	Unsat.	NB	NB
**259**	Proteus vulgaris O2 (OX2)	NB	NB	NB
**260**	Proteus mirabilis O3ab (S1959)	Unsat.	NB	NB
**261**	Proteus mirabilis O5 (PrK 12/57)	NB	NB	NB
**262**	Proteus mirabilis O9 (PrK 18/57)	Unsat.	NB	NB
**263**	Proteus mirabilis O11 (9B-m)	NB	Unsat.	NB
**264**	Proteus penneri O17 (16)	Unsat.	NB	NB
**265**	Proteus mirabilis O18 (PrK 34/57)	Unsat.	NB	NB
**266**	Proteus mirabilis O20 (PrK 38/57)	Unsat.	Unsat.	NB
**267**	Proteus penneri O31ab (28)	NB	NB	NB
**268**	Proteus mirabilis O33 (D52)	NB	NB	NB
**269**	Proteus mirabilis O43 (PrK 69/57)	NB	NB	NB
**270**	Proteus vulgaris O47 (PrK 73/57)	**0.34**	unsat.	**0.11**
**271**	Proteus mirabilis O49 (PrK 75/57)	Unsat.	NB	NB
**272**	Proteus mirabilis O54ab (OE)	Unsat.	unsat.	unsat.
**273**	Proteus penneri O73ac (75)	Unsat.	unsat.	unsat.
**274**	Proteus vulgaris O76 (HSC438)	Unsat.	NB	NB
**275**	Shigella flexneri type 1a	Unsat.	NB	NB
**276**	Shigella flexneri type 1b	Unsat.	NB	NB
**277**	Shigella flexneri type 2a	Unsat.	NB	NB
**278**	Shigella flexneri type 2b	Unsat.	NB	NB
**279**	Shigella flexneri type 3a	Unsat.	unsat.	NB
**280**	Shigella flexneri type 3b	NB	NB	NB
**281**	Shigella flexneri type 4a	Unsat.	unsat.	NB
**282**	Shigella flexneri type 4b	NB	NB	NB
**283**	Shigella flexneri type 5b	NB	unsat.	NB
**284**	Shigella flexneri type 6a	NB	NB	NB
**285**	Shigella flexneri type 6	Unsat.	unsat.	NB
**286**	Shigella flexneri type X	NB	NB	NB
**287**	Shigella dysenteriae type 1	Unsat.	NB	NB

**288**	Shigella boydii type 6	NB	NB	NB
**289**	Shigella boydii type 7	Unsat.	NB	NB
**290**	Shigella boydii type 8	Unsat.	NB	NB
**291**	Shigella boydii type 13	Unsat.	Unsat.	NB
**292**	Shigella boydii type 14	Unsat.	NB	NB
**293**	Escherichia coli O71	NB	NB	NB
**294**	Escherichia coli O85	Unsat.	NB	NB
**295**	Escherichia coli O99	NB	NB	NB
**296**	Escherichia coli O145	Unsat.	Unsat.	NB
**297**	Escherichia coli O107	Unsat.	NB	NB
**298**	Salmonella enterica O17	Unsat.	NB	NB
**299**	Salmonella enterica O28	Unsat.	NB	NB
**300**	Salmonella enterica O47	Unsat.	NB	NB
**301**	Salmonella enterica O55	NB	NB	NB
**302**	Escherichia coli K92	NB	NB	NB
**303**	Escherichia coli K5	NB	NB	NB
**304**	Escherichia coli K13	Unsat.	NB	NB
**305**	Neisseria meningitidis Group C	Unsat.	Unsat.	NB
**306**	Escherichia coli O86	**0.85**	NB	**9.48**
