# Supplementary material for: Galectin-9 recognizes and exhibits antimicrobial activity toward microbes expressing blood group–like antigens
Source: J Biol Chem. 2022 Feb 9;298(4):101704. doi: 10.1016/j.jbc.2022.101704 (PMC9019251; doi:10.1016/j.jbc.2022.101704)
Supplement: Supplemental Table S3 [file mmc4.pdf]

Table S3. MGM array legend of microbial glycans

Chart#		BACTERIA / STRAIN	NAME / STRUCTURE /Cat.No.	STRUCTURE
1		Providencia stuartii O49	PO49 Core-linked	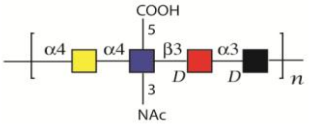
2		Providencia stuartii O52	PO52 Core-linked	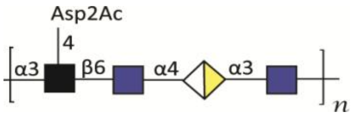
3		Pseudomonas aerguinosa O4 (Habs serotype 4)	PO4 Core-linked	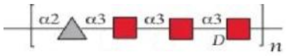
4		Pseudomonas aerguinosa O1 (Fisher immunotype 4)	PO1 Core-linked	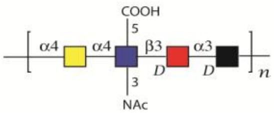
5		Pseudomonas aerguinosa O2 (Fisher immunotype 3)	PO2 Core-linked	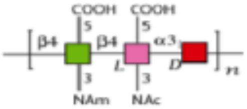
6		Pseudomonas aerguinosa O13 (Sandvik serotype II)	PO13 Core-linked	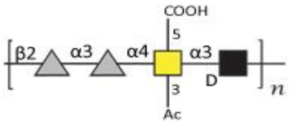
7		Pseudomonas aerguinosa O9 (9a, 9b, 9d)	PO9 Core-linked	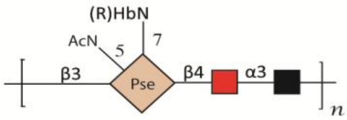
8		Pseudomonas aerguinosa O6a (Habs serotype6, fraction IIa)	PO6a Core-linked-O-unit	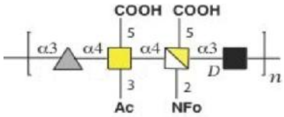
9		Pseudomonas aerguinosa O6a (Habs serotype6, fraction IIb)	PO6a unsubstituted core	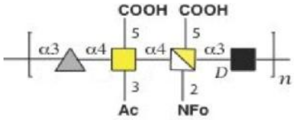
10		Salmonella typhimurium SL 11881 (Re mut)	LPS-L9516	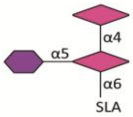
11		Salmonella typhimurium TV 119 (Ra mut)	LPS-L6016	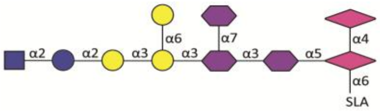
12		Salmonella typhimurium SL 684 (Rc mut)	LPS-L5891	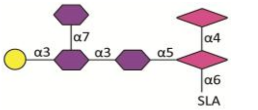

13		Pseudomonas aerguinosa O10	L8643	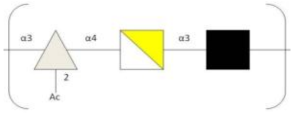
14		Salmonella typhimurium dodeca saccharide	4809	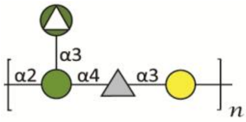
15		Salmonella enteritidis dodeca saccharide	1262	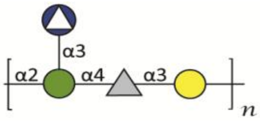
16		Salmonella typhimurium LPS	L2262	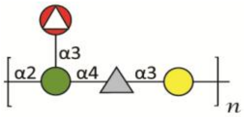
17		Serratia marcescens LPS	L6136	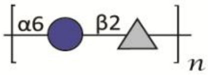
18		Escherichia coli K235 LPS	L2143	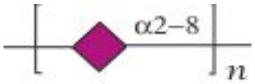
19		Escherichia coli O128-B12 LPS	L2755	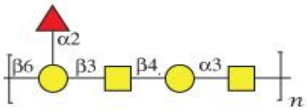
20		Salmonella enterica abortus equi LPS	L5886	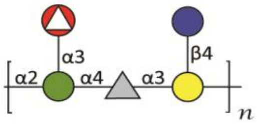
21		Salmonella typhosa LPS	L2387	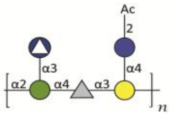
22		Salmonella enteritidis LPS	L2012	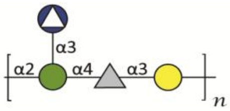
23		Shigella bodyii type2		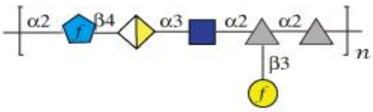
24		Shigella bodyii type4		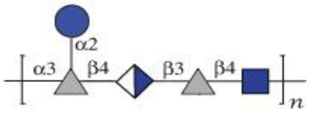
25		Shigella bodyii type10		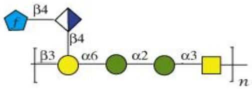

26		Shigella dysennteriae type 3		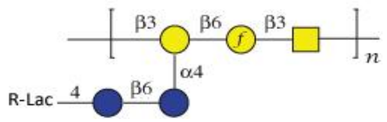
27		Shigella dysennteriae type 8 (batch 12)		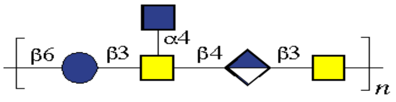
28		Shigella dysennteriae type 11		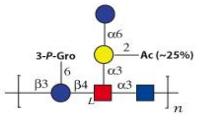
29		Shigella dysennteriae type 13		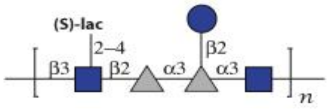
30		Escherichia coli O29		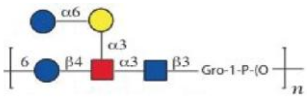
31		Escherichia coli O40		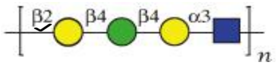
32		Escherichia coli O106		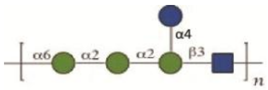
33		Escherichia coli O130		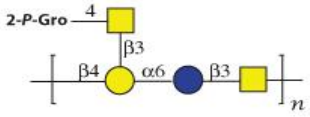
34		Escherichia coli O148		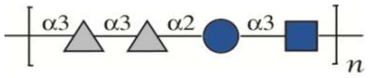
35		Escherichia coli O150		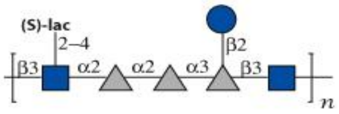
36		Escherichia coli O180		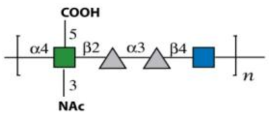
37		Proteus mirabilis O3a, 3c (G1)		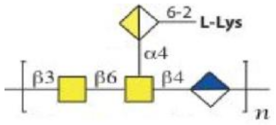
38		Proteus mirabilis O8 (TG326)		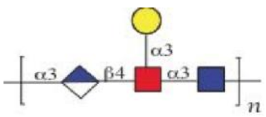

39		Proteus mirabilis O10 (HJ4320)		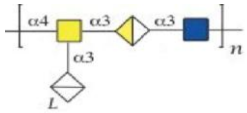
40		Proteus mirabilis O29a, 29b (2002)		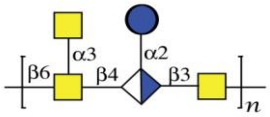
41		Proteus mirabilis O50 (TG332)		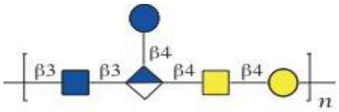
42		Proteus mirabilis O54a, 54b (10704)		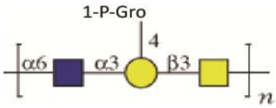
43		Proteus mirabilis O57 (TG319)		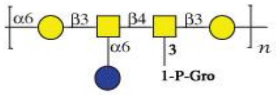
44		Proteus penneri O8 (106)		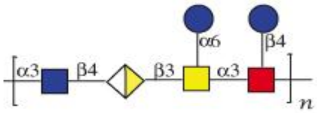
45		Proteus penneri O64a, 64b, 64d (39)		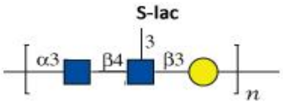
46		Proteus penneri O66 (2)		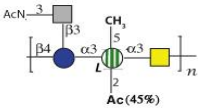
47		Proteus penneri O69 (25)		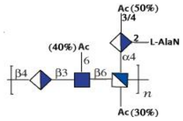
48		Proteus penneri O71 (42)		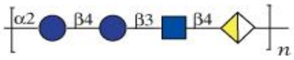
49		Proteus penneri O72a, 72b (4)		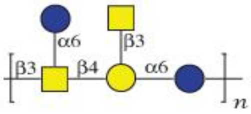
50		Pseudomonas aerguinosa O2 (2a),2d,2f	IATS 10 , OPS	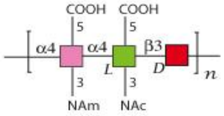
51		Pseudomonas aerguinosa O2 2a,2b	IATS 16 OPS	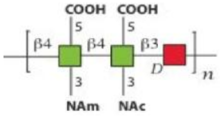

52		Pseudomonas aerguinosa O2 2a,2b,2e	IATS NO, OPS	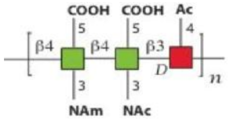
53		Pseudomonas aerguinosa O2 2a,2d	IATS 5 OPS	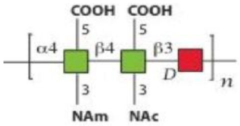
54		Pseudomonas aerguinosa O2 Immuno 7	IATS 18, OPS	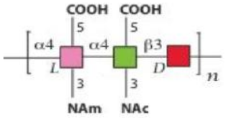
55		Pseudomonas aerguinosa O3 3a,3b	IATS NO, OPS	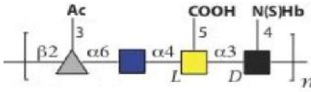
56		Pseudomonas aerguinosa O3 3a,3b,3c	IATS 3, OPS	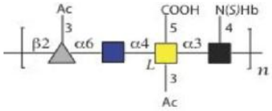
57		Pseudomonas aerguinosa O3 3a,3d	IATS NO, OPS	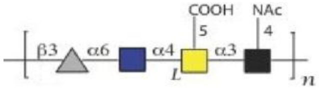
58		Pseudomonas aerguinosa O4 4a,4c	IATS NO, OPS	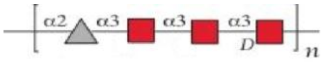
59		Pseudomonas aerguinosa O6 6a	IATS 6, OPS	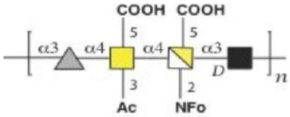
60		Pseudomonas aerguinosa O6 6a,6c	IATS NO, OPS	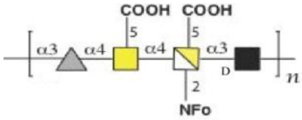
61		Pseudomonas aerguinosa O6 Immuno 1	IATS NO, OPS	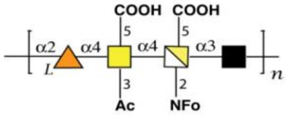
62		Pseudomonas aerguinosa O7 7a,7b,7c	IATS 7,LPS	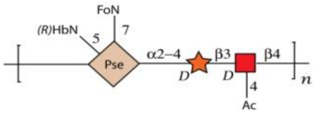
63		Pseudomonas aerguinosa O7 7a,7b,7d	IATS 8,LPS	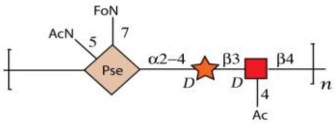
64		Pseudomonas aerguinosa O7 7a,7d	IATS NO, LPS	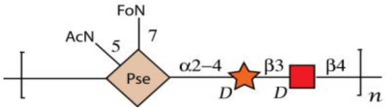

65		Pseudomonas aerguinosa O10 10a,10b	IATS 10, OPS	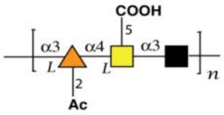
66		Pseudomonas aerguinosa O10 10a,10c	IATS 19, OPS	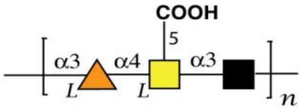
67		Pseudomonas aerguinosa O11 11a,11b	IATS 11, OPS	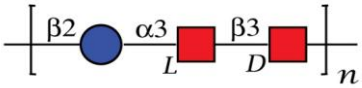
68		Pseudomonas aerguinosa O12 12	IATS 12, OPS Habs 12	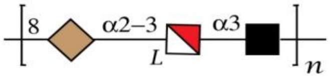
69		Pseudomonas aerguinosa O13 13a,13c	IATS 14, OPS	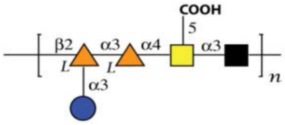
70		Pseudomonas aerguinosa O14 14	IATS 17,OPS Meitert X	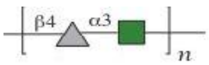
71		Pseudomonas aerguinosa O15 15	IATS 15, OPS	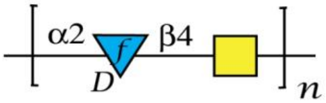
72		Proteus vulgaris O1 (18984)*	LPS	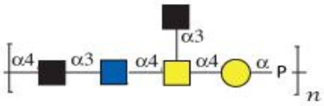
73		Proteus vulgaris O4 (PrK 9/57)	OPS	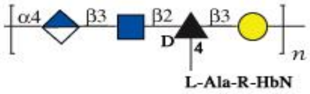
74		Proteus vulgaris O12 (PrK 25/57)	OPS	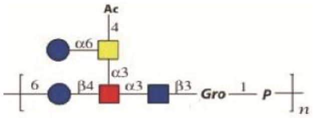
75		Proteus vulgaris O13 (8344)	OPS	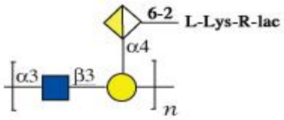
76		Proteus vulgaris O15 (PrK 30/57)	OPS	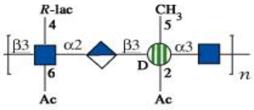
77		Proteus vulgaris O17 (PrK 33/57)	OPS	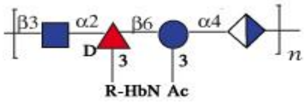

78		Proteus vulgaris O19a (PrK 37/57)	OPS	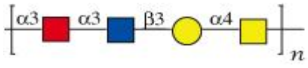
79		Proteus vulgaris O21 (PrK 39/57)*	LPS	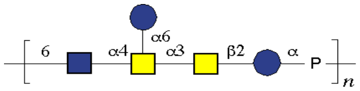
80		Proteus vulgaris O25 (PrK 48/57)	OPS	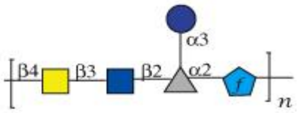
81		Proteus vulgaris O34 (4669)*	LPS	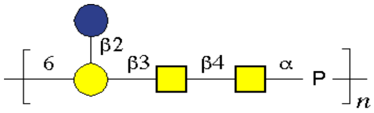
82		Proteus vulgaris O37a,b (PrK 63/57)	OPS	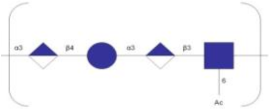
83		Proteus vulgaris O37a,c (PrK 72/57)	OPS	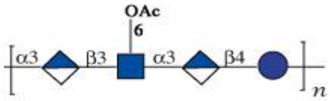
84		Proteus vulgaris O44 (PrK 67/57)	OPS	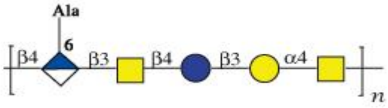
85		Proteus vulgaris O45 (4680)	OPS	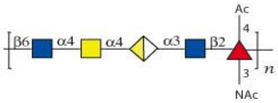
86		Proteus vulgaris O53 (TG 276-10)	OPS	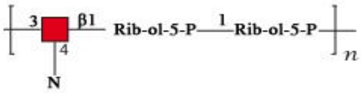
87		Proteus vulgaris O54a,54c (TG 103)	OPS	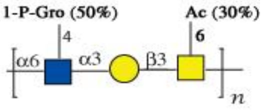
88		Proteus vulgaris O55 (TG 155)	OPS	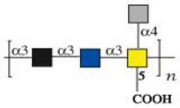
89		Proteus vulgaris O65 (TG 251)	OPS	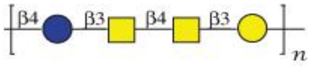

90		Proteus mirabilis O6 (PrK 14/57)	OPS	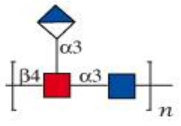
91		Proteus mirabilis O11 (PrK 24/57)	OPS	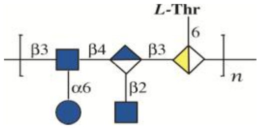
92		Proteus mirabilis O13 (PrK 26/57)	OPS	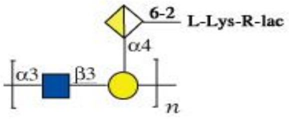
93		Proteus mirabilis O14a,14b (PrK 29/57)	OPS	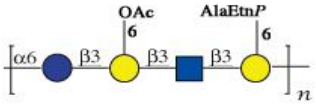
94		Proteus mirabilis O16 (4652)	OPS	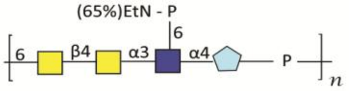
95		Proteus mirabilis O17 (PrK 32/57)	OPS	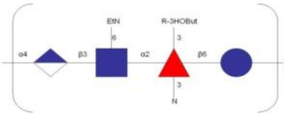
96		Proteus mirabilis O23a,b,d (PrK 42/57)	OPS	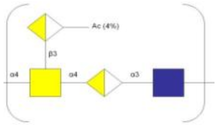
97		Proteus mirabilis O26 (PrK 49/57)	OPS	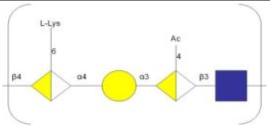
98		Proteus mirabilis O27 (PrK 50/57)	OPS	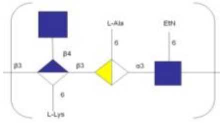
99		Proteus mirabilis O28 (PrK 51/57)	OPS	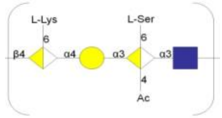
100		Proteus mirabilis O29a (PrK 52/57)	OPS	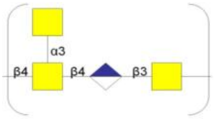
101		Proteus mirabilis O40 (10703)	OPS	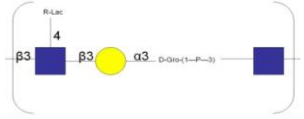
102		Proteus mirabilis O41 (PrK 67/57)	OPS	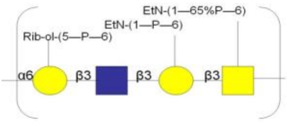

103		Proteus mirabilis O51 (19011)*	LPS	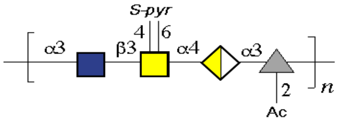
104		Proteus mirabilis O74 (10705, OF)	OPS	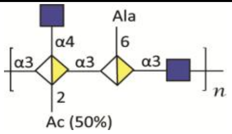
105		Proteus mirabilis O75 (10702, OC)	OPS	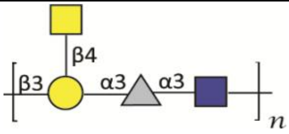
106		Proteus mirabilis O77 (3 B-m)	OPS	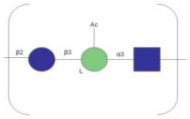
107		Proteus penneri O31a (26)	OPS	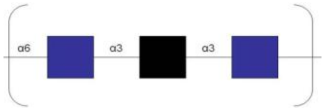
108		Proteus penneri O52 (15)	OPS	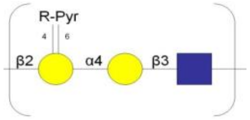
109		Proteus penneri O58 (12)	OPS	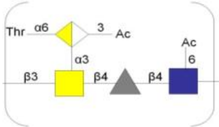
110		Proteus penneri O59 (9)	OPS	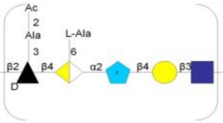
111		Proteus penneri O61 (21)	OPS	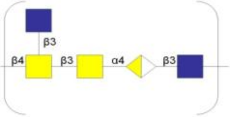
112		Proteus penneri O62 (41)	OPS	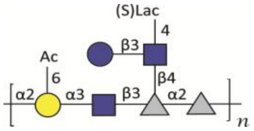
113		Proteus penneri O63 (22)	OPS	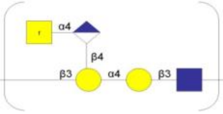
114		Proteus penneri O64a,b,c (27)	OPS	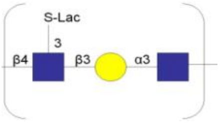
115		Proteus penneri O65 (34)	OPS	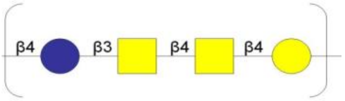

116		Proteus penneri O67 (8)	OPS	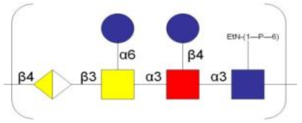
117		Proteus penneri O68 (63)	OPS	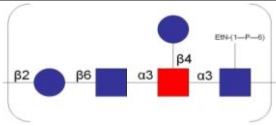
118		Proteus penneri O70 (60)	OPS	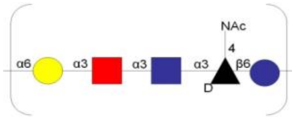
119		Proteus penneri O73a,b (103)	OPS	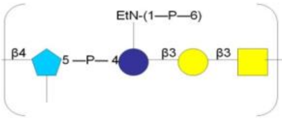
120		Proteus myxofaciens O60	OPS	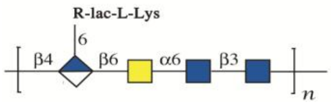
121		Proteus O56 (genomospecies 4)	OPS	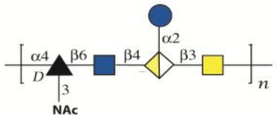
122		Providencia stuartii O4	OPS	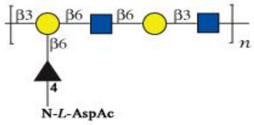
123		Providencia stuartii O18	OPS	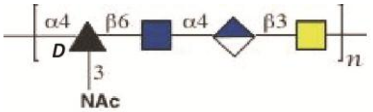
124		Providencia stuartii O20*	LPS	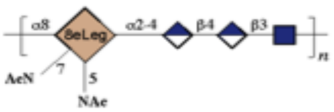
125		Providencia stuartii O43	OPS	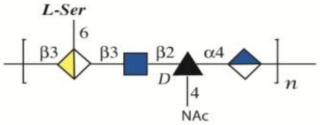
126		Providencia stuartii O44	OPS	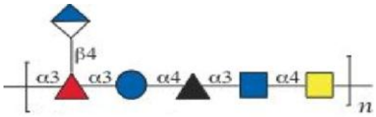
127		Providencia stuartii O47	OPS	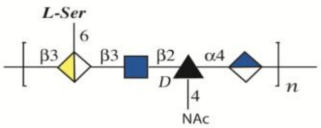
128		Providencia stuartii O47, Core 9	OPS	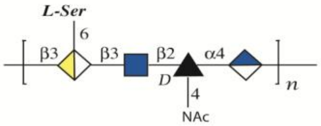

129		Providencia stuartii O49, Core 1	OPS	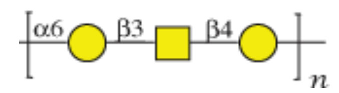
130		Providencia stuartii O57	OPS	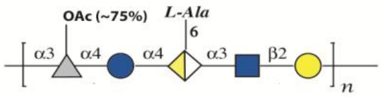
131		Providencia alcalifaciens O5	OPS	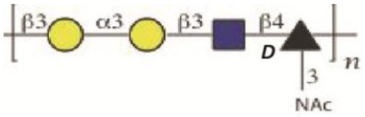
132		Providencia alcalifaciens O6*	LPS	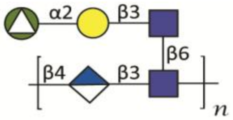
133		Providencia alcalifaciens O19	OPS	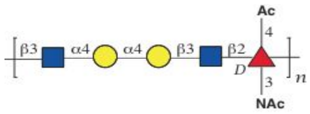
134		Providencia alcalifaciens O19	LPS	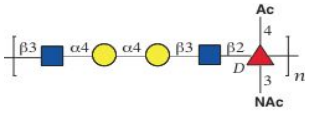
135		Providencia alcalifaciens O19	LPS/NaOH	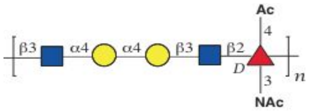
136		Providencia alcalifaciens O21	OPS	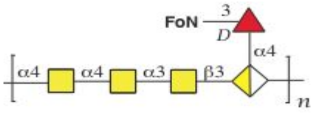
137		Providencia alcalifaciens O23	OPS	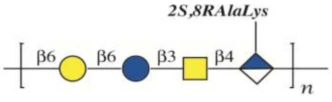
138		Providencia alcalifaciens O27	OPS	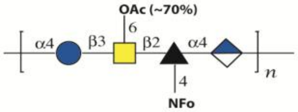
139		Providencia alcalifaciens O29	OPS	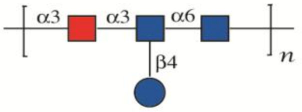
140		Providencia alcalifaciens O30	OPS	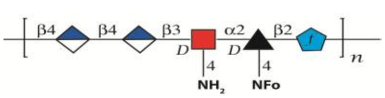
141		Providencia alcalifaciens O32	OPS	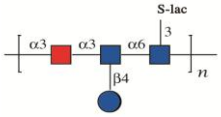

142		Providencia alcalifaciens O36*	LPS-NH4OH	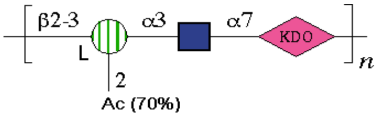
143		Providencia alcalifaciens O39	OPS	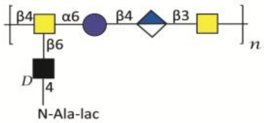
144		Providencia rustigianii O14	OPS	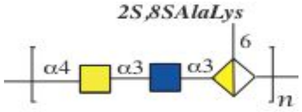
145		Providencia rustigianii O16	OPS	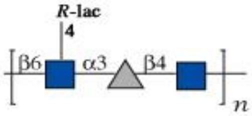
146		Providencia rustigianii O34	OPS	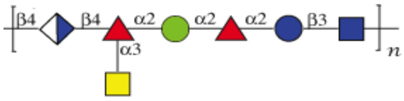
147		Yersinia pestis, KM260(11)-∆0187	LPS	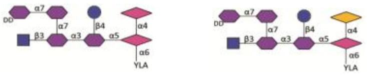
148		Yersinia pestis, KM260(11)-∆0187	Core oligo saccharide	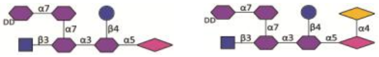
149		Yersinia pestis, KM260(11)-∆rfe	LPS	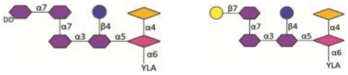
150		Yersinia pestis, KM260(11)-∆rfe	Core oligo saccharide	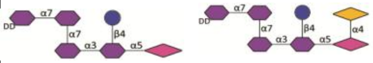
151		Yersinia pestis, 1146-25	LPS	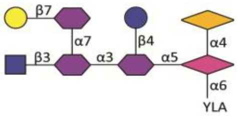
152		Yersinia pestis 1146-25	Core oligo saccharide	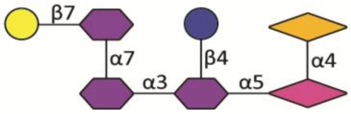
153		Yersinia pestis, 1146-37	LPS	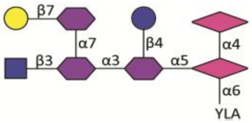
154		Yersinia pestis, 1146-37	Core oligo saccharide	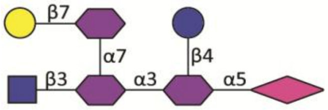

155		Yersinia pestis, KM218-37	LPS	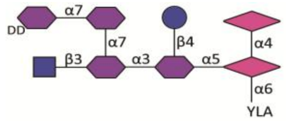
156		Yersinia pestis, KM218-37	Core oligo saccharide	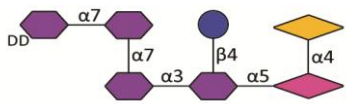
157		Yersinia pestis, KM218-25	LPS	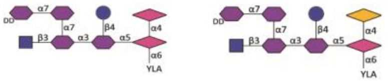
158		Yersinia pestis, KM218-25	Core oligo saccharide	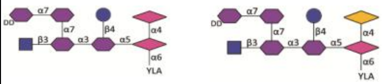
159		Yersinia pestis, KM260(11)-ΔpmrF	LPS	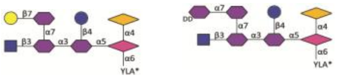
160		Yersinia pestis, KM260(11)-ΔpmrF	Core oligo saccharide	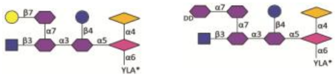
161		Yersinia pestis, KM260(11)-Δ0186	LPS	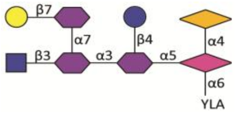
162		Yersinia pestis, KM260(11)-Δ0186	Core oligo saccharide	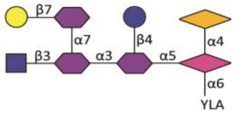
163		Yersinia pestis, KM260(11)-ΔwaaQ	LPS	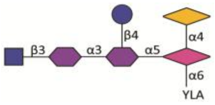
164		Yersinia pestis, KM260(11)-ΔwaaQ	Core oligo saccharide	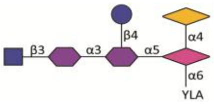
165		Yersinia pestis, KM260(11)-ΔwaaL	LPS	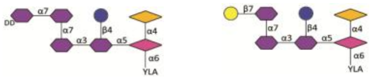
166		Yersinia pestis, KM260(11)-25	LPS	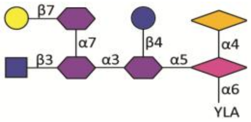
167		Yersinia pestis, KM260(11)-25	Core oligo saccharide	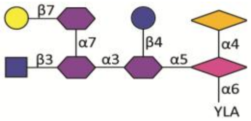

168		Yersinia pestis, KM260(11)-37	Core oligo saccharide	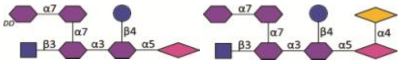
169		Yersinia pestis, KIMD1-37	Core oligo saccharide	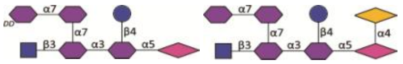
170		Yersinia pestis, KIMD1-25	Core oligo saccharide	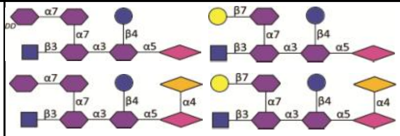
171		Yersinia pestis, 11M-25	LPS	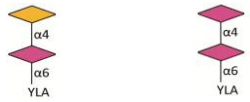
172		Yersinia pestis, 11M-37	LPS	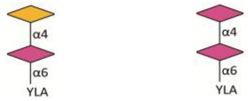
173		Proteus vulgaris O23a, 23b, 23c (CCUG 10701)	OPS	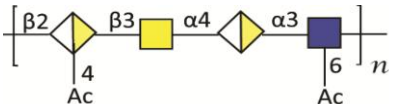
174		Proteus vulgaris O24 (PrK 47/57)	LPSOH	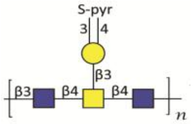
175		Yersinia pestis KM260(11)-6C	LPS	
176		Yersinia pestis 260(11)-37C-186	LPS	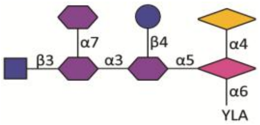
177		Yersinia pestis 260(11)-37C-187	LPS	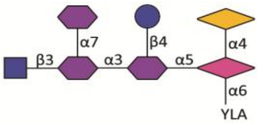
178		Yersinia pestis 260(11)-37C-416	LPS	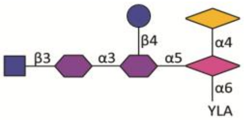
179		Yersinia pestis 260(11)-37C-417	LPS	
180		Yersinia pestis P-1680-25C	OS	

181		Yersinia pestis P-1680-37C	LPS	
182		Yersinia pestis I-2377-25C	OS	
183		Yersinia pestis I-2377-37C	LPS	
184		Francisella novicida OPS	OPS	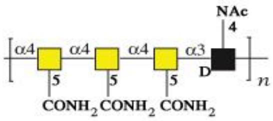
185		Francisella tularensis OPS	OPS	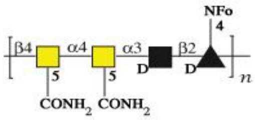
186		Klebsiella O1 OPS	OPS	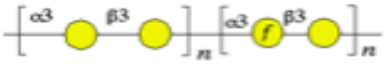
187		Klebsiella O2a OPS	OPS	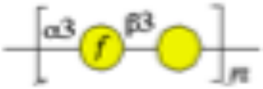
188		Klebsiella O2ac OPS	OPS	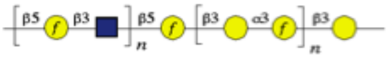
189		Klebsiella O3 OPS	OPS	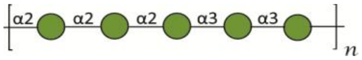
190		Klebsiella O4 OPS	OPS	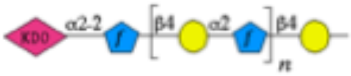
191		Klebsiella O5 OPS	OPS	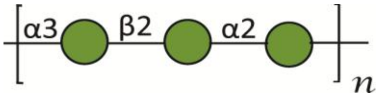
192		Klebsiella O8 OPS	OPS	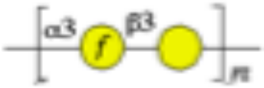
193		Klebsiella O12 OPS	OPS	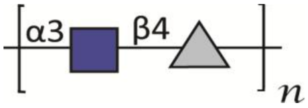

194		Shigella boydii type 1	LPSOH	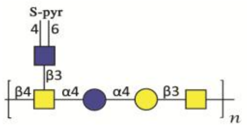
195		Shigella boydii type 3	OPS	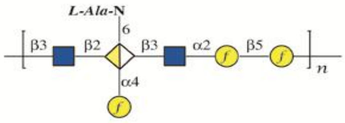
196		Shigella boydii type 5	OPS	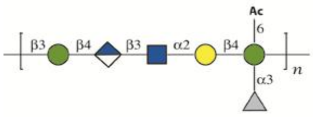
197		Shigella boydii type 9	OPS	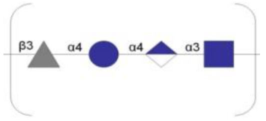
198		Shigella boydii type 11	OPS	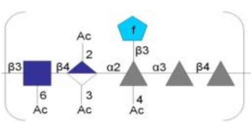
199		Shigella boydii type 12	OPS	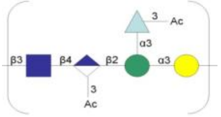
200		Shigella boydii type 15	OPS	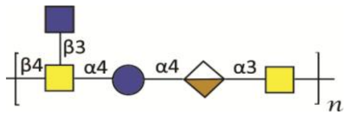
201		Shigella boydii type 16	OPS	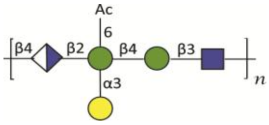
202		Shigella boydii type 17	OPS	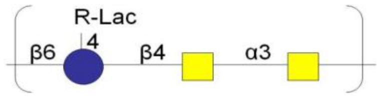
203		Shigella boydii type 18	OPS	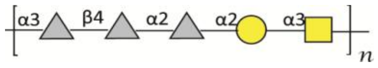
204		Escherichia coli O49	OPS	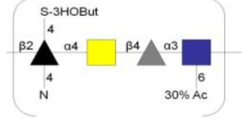
205		Escherichia coli O52	OPS	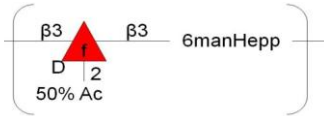
206		Escherichia coli O58	OPS	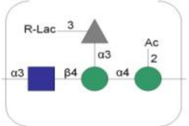

207		Escherichia coli O61	LPSOH	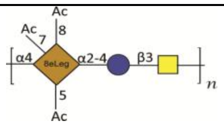
208		Escherichia coli O73	OPS	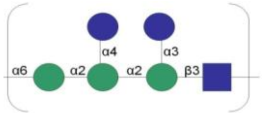
209		Escherichia coli O112ab	OPS	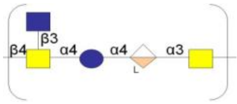
210		Escherichia coli O118	OPS	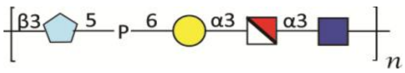
211		Escherichia coli O125	OPS	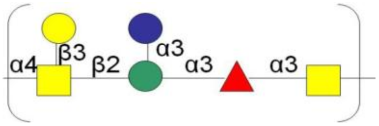
212		Escherichia coli O151	OPS	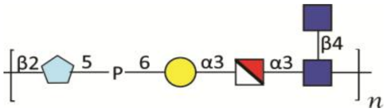
213		Escherichia coli O168	OPS	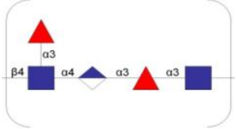
214		Shigella dysenteriae type 2	LPSOH	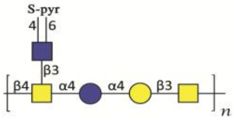
215		Shigella dysenteriae type 4	OPS	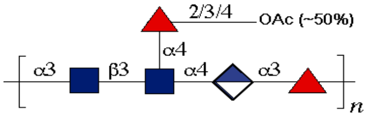
216		Shigella dysenteriae type 5	OPS	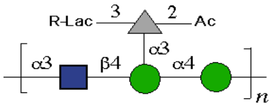
217		Shigella dysenteriae type 6 SR-strain	SR-strain	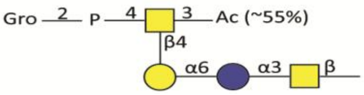
218		Shigella dysenteriae type 7	OPS	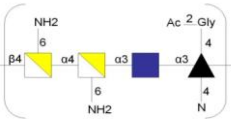
219		Shigella dysenteriae type 8 (Russian)	OPS	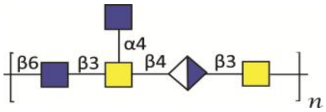

220		Shigella dysenteriae type 9	OPS	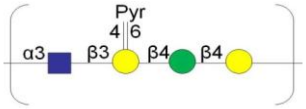
221		Escherichia coli O111:B4 LPS-solution at 1 mg/mL	L5293-2ML (LPS) (Sigma)	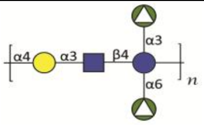
222		Escherichia coli O26:B6 LPS-solution at 1 mg/mL	L5543-2ML (LPS) (Sigma)	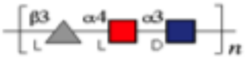
223		Escherichia coli O55:B5 LPS-solution at 1 mg/mL	L5418-2ML (LPS) (Sigma)	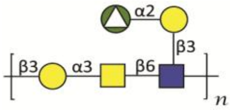
224		Escherichia coli O127:B8 LPS-solution at 1 mg/mL	L5668-2ML (LPS) (Sigma)	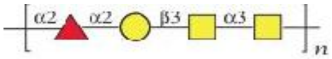
225		Streptococcus pneumoniae type 1 (Danish type 1)	161-X // Capsular PS	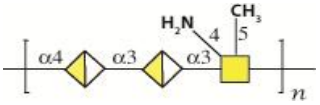
226		Streptococcus pneumoniae type 2 (Danish type 2)	165-X// Capsular PS	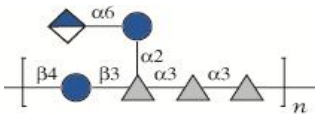
227		Streptococcus pneumoniae type 3 (Danish type 3)	169-X// Capsular PS	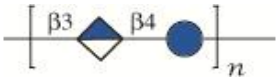
228		Streptococcus pneumoniae type 4 (Danish type 4)	173-X// Capsular PS	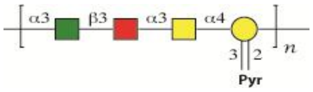
229		Streptococcus pneumoniae type 5 (Danish type 5)	177-X// Capsular PS	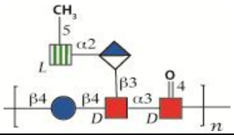
230		Streptococcus pneumoniae type 8 (Danish type 8)	185-X// Capsular PS	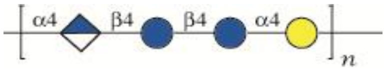
231		Streptococcus pneumoniae type 9 (Danish type 9N)	189-X// Capsular PS	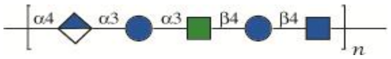
232		Streptococcus pneumoniae type 12 (Danish type 12F)	193-X// Capsular PS	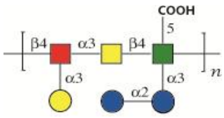

233		Streptococcus pneumoniae type 14 (Danish type 14)	197-X// Capsular PS	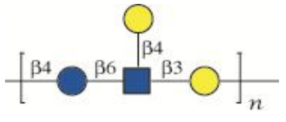
234		Streptococcus pneumoniae type 17 (Danish type 17F)	201-X// Capsular PS	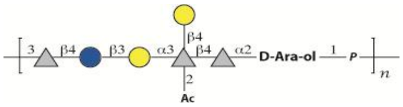
235		Streptococcus pneumoniae type 19 (Danish type 19F)	205-X// Capsular PS	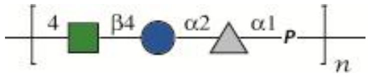
236		Streptococcus pneumoniae type 20 (Danish type 20)	209-X// Capsular PS	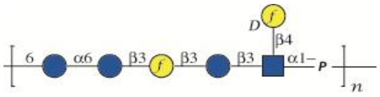
237		Streptococcus pneumoniae type 22 (Danish type 22F)	213-X// Capsular PS	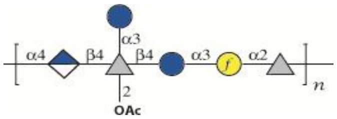
238		Streptococcus pneumoniae type 23 (Danish type 23F)	217-X// Capsular PS	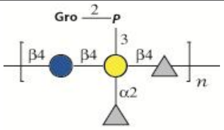
239		Streptococcus pneumoniae type 26 (Danish type 6B)	225-X// Capsular PS	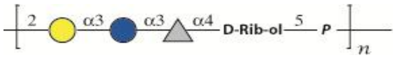
240		Streptococcus pneumoniae type 34 (Danish type 10A)	229-X// Capsular PS	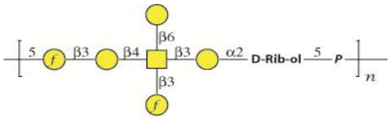
241		Streptococcus pneumoniae type 43 (Danish type 11A)	233-X// Capsular PS	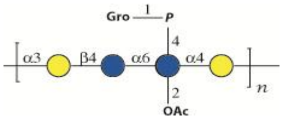
242		Streptococcus pneumoniae type 51 (Danish type 7F)	237-X// Capsular PS	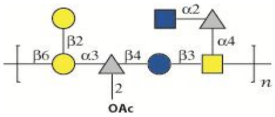
243		Streptococcus pneumoniae type 54 (Danish type 15B)	241-X// Capsular PS	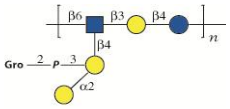
244		Streptococcus pneumoniae type 56 (Danish type 18C)	245-X// Capsular PS	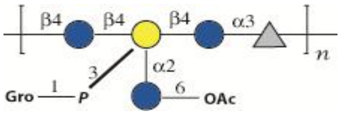
245		Streptococcus pneumoniae type 57 (Danish type 19A)	249-X// Capsular PS	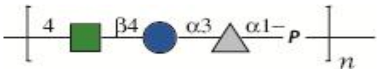

246		Streptococcus pneumoniae type 68 (Danish type 9V)	253-X// Capsular PS	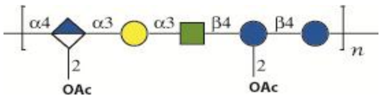
247		Streptococcus pneumoniae type 70 (Danish type 33F)	257-X// Capsular PS	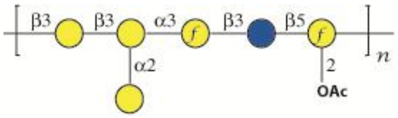
248		Yersinia pestis KM218-6C	OS	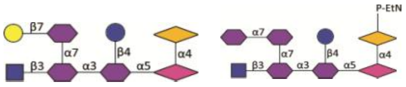
249		Yersinia pestis KM260(11)-yjhW-6C	OS	
250		Yersinia pestis KM260(11)-wabD/waaL	OS	
251		Yersinia pestis KM260(11)-wabC/waaL	OS	
252		Yersinia pseudotuberculosis 85pCad-37C	OS	
253		Yersinia pseudotuberculosis 85pCad-20C	OS	
254		Yersinia pseudotuberculosis O:2a	PS	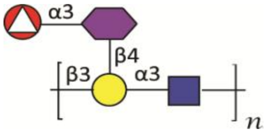
255		Yersinia pseudotuberculosis O:2a-dhmA	PS	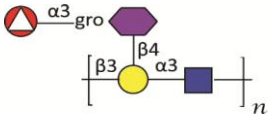
256		Yersinia pseudotuberculosis O:2c	PS	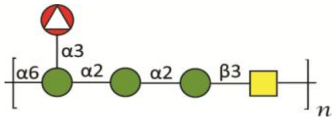
257		Yersinia pseudotuberculosis O:3	PS	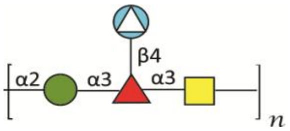
258		Yersinia pseudotuberculosis O:4b	PS	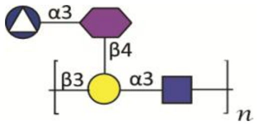

259		Proteus vulgaris O2 (OX2)	PS	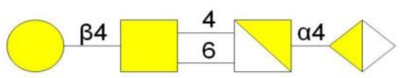
260		Proteus mirabilis O3ab (S1959)	PS	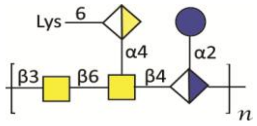
261		Proteus mirabilis O5 (PrK 12/57)	PS	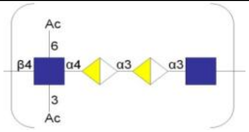
262		Proteus mirabilis O9 (PrK 18/57)	PS	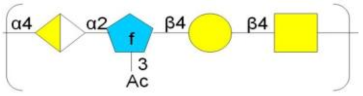
263		Proteus mirabilis O11 (9B-m)	PS	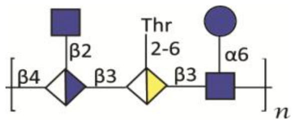
264		Proteus penneri O17 (16)	PS	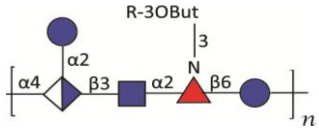
265		Proteus mirabilis O18 (PrK 34/57)	LPSOH	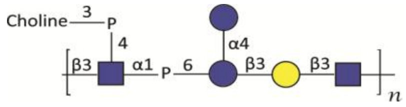
266		Proteus mirabilis O20 (PrK 38/57)	LPSOH	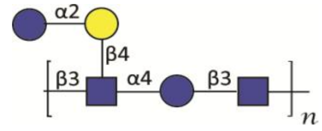
267		Proteus penneri O31ab (28)	PS	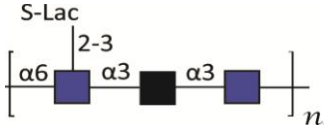
268		Proteus mirabilis O33 (D52)	PS	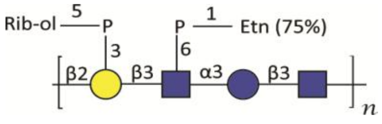
269		Proteus mirabilis O43 (PrK 69/57)	PS	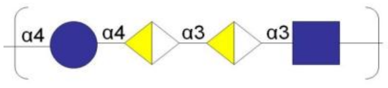
270		Proteus vulgaris O47 (PrK 73/57)	Not stated	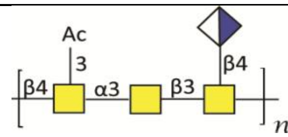
271		Proteus mirabilis O49 (PrK 75/57)	PS	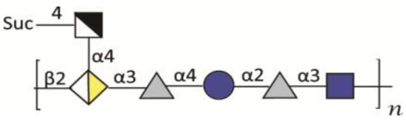

272		Proteus mirabilis O54ab (OE)	PS	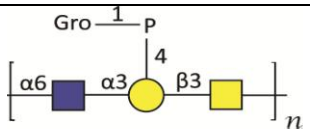
273		Proteus penneri O73ac (75)	PS	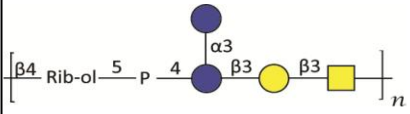
274		Proteus vulgaris O76 (HSC438)	PS	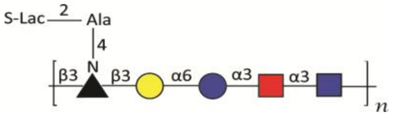
275		Shigella flexneri type 1a	PS	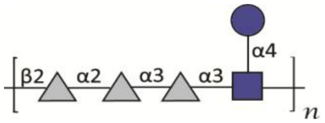
276		Shigella flexneri type 1b	PS	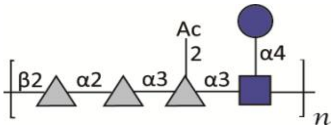
277		Shigella flexneri type 2a	PS	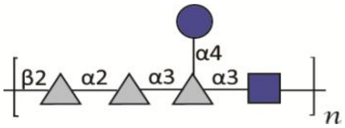
278		Shigella flexneri type 2b	PS	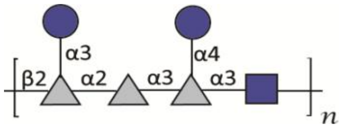
279		Shigella flexneri type 3a	PS	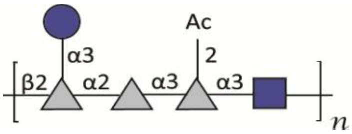
280		Shigella flexneri type 3b	PS	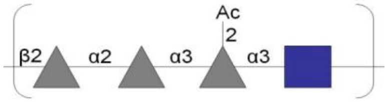
281		Shigella flexneri type 4a	PS	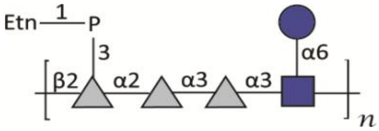
282		Shigella flexneri type 4b	PS	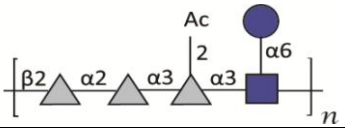
283		Shigella flexneri type 5b	PS	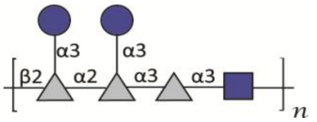
284		Shigella flexneri type 6a	PS	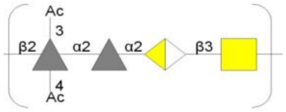

285		Shigella flexneri type 6	PS	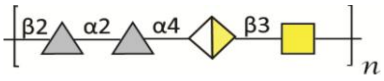
286		Shigella flexneri type X	PS	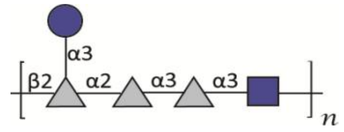
287		Shigella dysenteriae type 1	PS	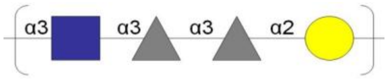
288		Shigella boydii type 6	PS	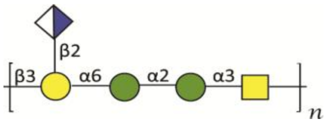
289		Shigella boydii type 7	PS	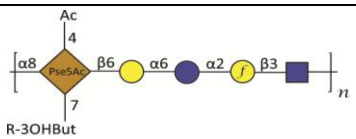
290		Shigella boydii type 8	PS	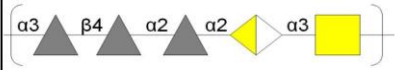
291		Shigella boydii type 13	LPSOH	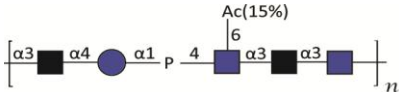
292		Shigella boydii type 14	LPSOH	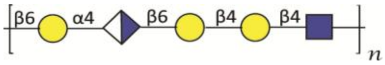
293		Escherichia coli O71	PS	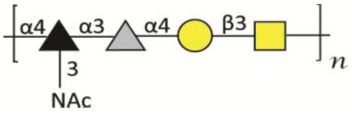
294		Escherichia coli O85	PS	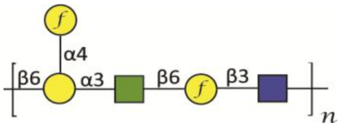
295		Escherichia coli O99	PS	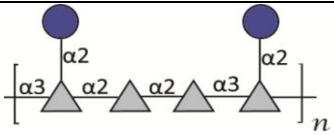
296		Escherichia coli O145	LPSOH	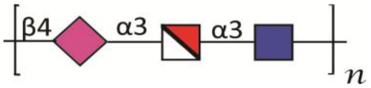
297		Escherichia coli O107	PS	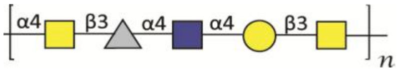

<table>
<tr><td>298</td><td></td><td>Salmonella enterica O17</td><td>PS</td><td>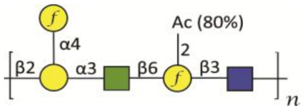
</td></tr>
<tr><td>299</td><td></td><td>Salmonella enterica O28</td><td>PS</td><td>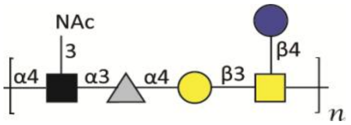
</td></tr>
<tr><td>300</td><td></td><td>Salmonella enterica O47</td><td>PS</td><td>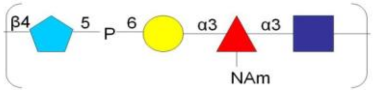
</td></tr>
<tr><td>301</td><td></td><td>Salmonella enterica O55</td><td>PS</td><td>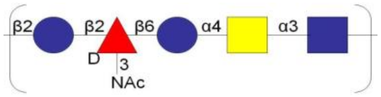
</td></tr>
<tr><td>302</td><td></td><td>Escherichia coli K92</td><td>CPS</td><td>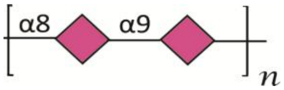
</td></tr>
<tr><td>303</td><td></td><td>Escherichia coli K5</td><td>CPS</td><td>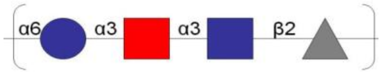
</td></tr>
<tr><td>304</td><td></td><td>Escherichia coli K13</td><td>CPS</td><td>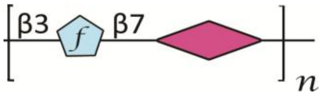
</td></tr>
<tr><td>305</td><td></td><td>Neisseria meningitidis Group C</td><td>CPS</td><td>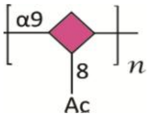
</td></tr>
</table>

<table>
<tr><td>306</td><td></td><td>Escherichia coli O86</td><td></td><td>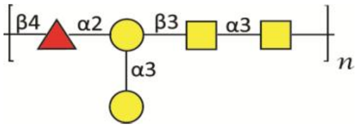
</td></tr>
</table>
